# Implications of past and present genetic connectivity for management of the saltwater crocodile (*Crocodylus porosus*)

**DOI:** 10.1111/eva.13545

**Published:** 2023-03-31

**Authors:** Luke R. Lloyd‐Jones, Matthew L. Brien, Pierre Feutry, Emma Lawrence, Paul Beri, Simon Booth, Steven Coulson, Shane M. Baylis, Kira Villiers, Laurence E. Taplin, David A. Westcott

**Affiliations:** ^1^ Commonwealth Scientific and Industrial Research Organisation Data61 Brisbane Queensland 4072 Australia; ^2^ Department of Environment and Science Queensland Government Cairns Queensland 4870 Australia; ^3^ Commonwealth Scientific and Industrial Research Organisation Oceans and Atmosphere Hobart Tasmania 7000 Australia; ^4^ Commonwealth Scientific and Industrial Research Organisation Land and Water Atherton Queensland 4883 Australia

**Keywords:** *Crocodylus porosus*, kinship, population genetics, population structure, RAD, sequence capture

## Abstract

Effective management of protected species requires information on appropriate evolutionary and geographic population boundaries and knowledge of how the physical environment and life‐history traits combine to shape the population structure and connectivity. Saltwater crocodiles (*Crocodylus porosus*) are the largest and most widely distributed of living crocodilians, extending from Sri Lanka to Southeast Asia and down to northern Australia. Given the long‐distance movement capabilities reported for *C. porosus*, management units are hypothesised to be highly connected by migration. However, the magnitude, scale, and consistency of connection across managed populations are not fully understood. Here we used an efficient genotyping method that combines DArTseq and sequence capture to survey ≈3000 high‐quality genome‐wide single nucleotide polymorphisms from 1176 *C. porosus* sampled across nearly the entire range of the species in Queensland, Australia. We investigated historical and present‐day connectivity patterns using fixation and diversity indices coupled with clustering methods and the spatial distribution of kin pairs. We inferred kinship using forward simulation coupled with a kinship estimation method that is robust to unspecified population structure. The results demonstrated that the *C. porosus* population has substantial genetic structure with six broad populations correlated with geographical location. The rate of gene flow was highly correlated with spatial distance, with greater differentiation along the east coast compared to the west. Kinship analyses revealed evidence of reproductive philopatry and limited dispersal, with approximately 90% of reported first and second‐degree relatives showing a pairwise distance of <50 km between sampling locations. Given the limited dispersal, lack of suitable habitat, low densities of crocodiles and the high proportion of immature animals in the population, future management and conservation interventions should be considered at regional and state‐wide scales.

## 1 INTRODUCTION

Understanding the evolutionary history and present distribution of genetic variation of wild species is fundamental knowledge in which to base informed decisions for sustainable management (Waples et al., 2008). Modern genetic data and analytical advances can help to determine appropriate evolutionary and geographic boundaries for management and identify how the biophysical environment and life‐history traits, such as habitat specificity or dispersal, combine to shape the population genetic structure and connectivity among populations (Hendricks et al., 2018; Hohenlohe et al., 2021). Genetic data can help identify dispersal events, estimate rates of effective migration, enable modelling of the effects of environmental conditions on dispersal within and among populations, and assess abundance (Al‐Asadi et al., 2019; Bravington et al., 2017; Feutry et al., 2017; Manel & Holderegger, 2013). The concepts and technology embedded in modern genetics and genomics offer a great opportunity to efficiently study in nature management‐relevant genetic factors of threatened, protected, or exploited species.

When managing wildlife, understanding population connectivity is particularly important with implications for identifying key habitats and corridors that connect populations, determine the potential impacts of human activities on these connections, inform decisions about reintroduction and translocation efforts, and strategies for maintaining population genetic diversity (Frankham, 2015; Jangjoo et al., 2016; Tewksbury et al., 2002). Management decisions require knowledge of the dependence of discrete populations on local reproduction versus immigration from dispersal, locations of potential sources and sinks across the species' managed range, and predicted population growth rates and size (Lowe & Allendorf, 2010; Mills & Allendorf, 1996; Perkins et al., 2003). Measuring dispersal directly at scales that are relevant to basic and applied questions is challenging, which has motivated the use of genetic methods to provide information on genetic connectivity, which is typically defined by the degree to which gene flow affects evolutionary processes within populations (Kool et al., 2013; Lowe & Allendorf, 2010). Divergence at individual genetic markers among subpopulations is commonly used to measure gene flow between spatially or temporally separated samples and is summarised using metrics such as *F*_ST_ and its analogues (Weir, 2012; Wright, 1949). These summaries require knowledge of population boundaries, which, if not available, can be estimated using unsupervised clustering algorithms for population assignment (Alexander et al., 2009; Pritchard et al., 2000). Multilocus genotype methods can also provide direct estimates of contemporary migration rates (Mussmann et al., 2019; Wilson & Rannala, 2003). An alternative approach to investigating dispersal and connectivity uses the spatial distribution of close relatives (Christie et al., 2010; Feutry et al., 2017). For example, the spatial distribution of half‐sibling pairs provides insight into breeding‐adult movements (Feutry et al., 2017). This approach provides complementary information to other population genetics methods and is at a time scale relevant to management planning and intervention. Kin pairs also form the basis of close‐kin mark‐recapture, which is an emerging method for robust population size estimation (Bravington, Grewe, & Davies, 2016; Bravington, Skaug, & Anderson, 2016; Hillary et al., 2018).

In this study, we apply population genetics techniques to inform population connectivity and management of the saltwater crocodile (*Crocodylus porosus*). The saltwater crocodile is an iconic element of the natural landscape of northern Australia, a remarkable example of resilience and recovery after being hunted to commercial extinction in the 1960s, and a dangerous predator that shares its habitat with humans—not always harmoniously. In Queensland, saltwater crocodiles have recovered slowly since they were protected in 1974 and now number 20–30,000 (Taplin et al., 2020). That recovery, combined with a rapidly increasing human population in its habitats, has seen increasing human‐crocodile conflict (Brien et al., 2017) and persistent calls since the 1980s for increased control measures ranging from egg‐harvesting to ‘culling’. The public discourse around crocodile conservation and management has always assumed that there is a single widespread Queensland crocodile population. However, this assumption is open to challenge. Taplin (1987) drew attention to the great diversity of Queensland's habitat in terms of its climate, physiography and human influences. Taplin (1987) identified 12 distinct ‘bioregions’ and subregions expected to pose quite different conservation and management issues. Those bioregions remain useful today in characterising the distribution and abundance of crocodiles State‐wide (Taplin et al., 2020; Taplin et al., 2021).

What has been lacking, however, is any understanding of the interconnectedness of crocodile sub‐populations occupying Queensland's bioregions. Saltwater crocodiles are capable of long‐distance movements extending over hundreds, even thousands, of kilometres—as evidenced by an individual appearing on a remote Pacific island (Allen, 1974) and satellite tracking observations of individuals moving from eastern to north‐western Cape York Peninsula following translocation (Campbell et al., 2013; Fukuda et al., 2019; Read et al., 2007). Together with long‐standing observations that juvenile and sub‐adult crocodiles disappear in large numbers from their natal areas (Messel et al., 1981, 1984), such observations have supported the idea that large‐scale movement, dispersal and consequent interbreeding are common in saltwater crocodiles. This lack of knowledge has important conservation and management consequences. A practical example arises in the far north of Queensland's populated east coast between Ingham and Cooktown, where a few thousand saltwater crocodiles occupy a narrow coastal strip with a high human population (Taplin et al., 2020). The area is bounded to the north and south by large stretches of quite inhospitable coastline with low crocodile densities. However, some 200 km north of Cooktown lies Lakefield‐Rinyirru National Park which has a substantial crocodile population that has increased quite rapidly in numbers and shows early signs of reaching some sort of saturation density (Taplin et al., 2020, 2021). Meanwhile, the northern populated east coast subpopulation has been subjected since the 1980s to a quite strict management regime that has seen some hundreds of crocodiles removed for public safety (Brien et al., 2017). Nonetheless, the crocodile population has increased since the 1980s at 2%–3% per annum (Taplin et al., 2020). An important question is whether that increase is driven by local recruitment, by immigration from a source population experiencing emigration pressure in Lakefield‐Rinyirru or both. If the northern east coast population is essentially closed with minimal immigration/emigration at its boundaries, then it needs to be managed accordingly. If, however, it experiences a high rate of immigration from Lakefield‐Rinyirru, then the management issues are quite different. The two subpopulations and those in the intermediate zones would have to be managed as a whole. Some targeting of migrating animals moving through habitat bottlenecks in inhospitable coastal areas might make sense, and the design and expected outcomes from local control efforts in the northern populated east coast would need to be modified.

Population genetics methods can assist management in characterising how this movement influences population boundaries, map population sources and sinks, informs the timing and degree of intervention required, and assign the provenance of problem crocodiles (Lowe & Allendorf, 2010; Manier & Arnold, 2005; Runge et al., 2006; Waples & Gaggiotti, 2006). The identification of management units is another central management goal with population analyses of genetic markers providing an indirect means of inferring how subpopulations aggregate (Palsbøll et al., 2007). Fukuda et al. (2022) found that dispersal range for saltwater crocodiles between source and destination in the Northern Territory was typically 150–200 km and up to 700 km, and concluded that regions that combine two or three adjacent catchments are an appropriate scale for population management. The appropriate scales for crocodile management in Queensland are currently unknown as are the relationships between the identified bioregions and the population structure. Taplin et al. (2021) identified that some bioregion boundaries warranted revision following analysis of distribution and abundance data in relation to climate. Genetic analyses can contribute to that revision. In addition, detailed contrasts can also be explored between the major management units of Queensland and the Northern Territory, whose genetic variation has been well characterised (Fukuda et al., 2019, 2022). These comparisons will help inform crocodile management across State boundaries. Genetic data further allow for an assessment of saltwater crocodile genetic diversity in Queensland following the 1960s demographic bottleneck, particularly at the southern extremes of the management range, where crocodiles are few in number, have increased in number very slowly since protection, and are subject to adverse climatic conditions as the world's southernmost saltwater crocodile population.

In this study, we used genome‐wide genetic data to perform a high‐resolution population genetics analysis of the Queensland *C. porosus* population that included individuals sampled from nearly the species' entire 4500 km Queensland range. To generate the genetic data on 1176 individuals, we used an efficient genotyping method that combines DArTseq on a pilot data set and subsequent sequence capture. Based on previous observations from the Northern Territory *C. porosus* population, we explored the hypothesis that the Queensland *C. porosus* population exhibits similar substantial genetic structure consistent with isolation by distance. Historical connectivity patterns were established using fixation and diversity indices coupled with clustering methods. We explored the implications of translocation, a historical component of the *C. porosus* management program, on genetic variation in the Queensland population. We investigated the spatial distribution of kin pairs using a kinship estimation method that is robust to unspecified population structure and validated it using forward simulation. Complementary lines of evidence from the coupled population genetics and kinship analyses were used to investigate hypotheses of dispersal distance, physical barriers, and mating system. The results provide new insights into saltwater crocodile connectivity across Queensland, provide a baseline for future genetic studies and methods, and contribute to the discussion of public safety and sustainable management of this iconic species.

## 2 MATERIALS AND METHODS

### 2.1 Study area

In Queensland, the saltwater crocodile is found throughout coastal areas, from the Fitzroy River near Rockhampton on the east coast, through Cape York Peninsula and the Torres Strait, and around the Gulf of Carpentaria to the Northern Territory border (Read et al., 2004; Taplin, 1987). There are 12 recognised crocodile bioregions and subregions, reflecting the highly variable biogeography and climatic conditions that exist across the state (Taplin, 1987; Taplin et al., 2020). While the species occupies a range of habitats including tidal and non‐tidal creeks, rivers, swamps, and wetlands, as well as beaches and offshore islands, it is predominantly riverine, with over 90% of the population below 20 m elevation (Taplin et al., 2020). The population has recovered and been increasing since protection in 1974 and is currently estimated at 20–30,000 non‐hatchlings at an average density of 1.65 individuals per km of river (Taplin et al., 2020). However, recovery has been highly variable across the bioregions, reflecting in part the availability of high‐quality breeding habitat. North‐western Cape York Peninsula has the most important breeding habitat and contains ≈40% of the population, with densities declining southward into the Gulf of Carpentaria and along Queensland's east coast (Taplin et al., 2020). Princess Charlotte Bay also contains important breeding habitat and may be a source of recruitment for nearby river systems, while the Fitzroy River contains the southernmost breeding population (Taplin et al., 2020). Overall, most of the crocodile habitat in Queensland is considered sub‐optimal.

### 2.2 Sampling and DNA extraction

**FIGURE 1:**
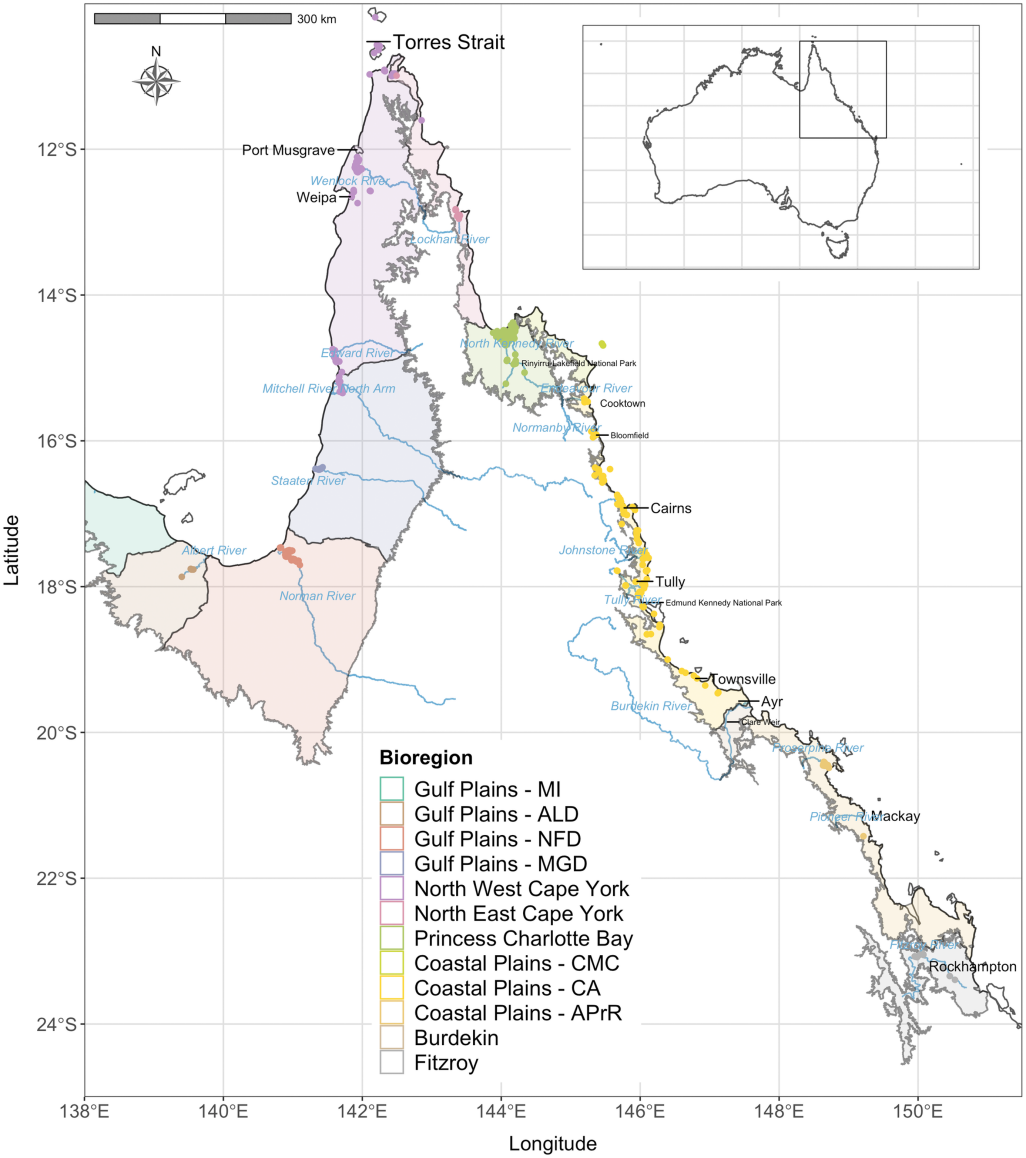
Map of saltwater crocodile study area across Queensland, Australia. Points correspond to sample instances of individual crocodiles and are coloured by saltwater crocodile bioregion (coloured polygons). Blue lines indicate large coastal rivers within each bioregion and are named in italic font. Major cities and places referenced in text are labelled in roman font. Bioregion abbreviations are ALD, Albert‐Leichhardt drainage; APrR, Ayr – Proserpine – Rockhampton; CA, Cooktown – Ayr; CMC, Cape Melville – Cooktown; MI, Massacre Inlet; MGD, Mitchell‐Gilbert drainage; NFD, Norman‐Flinders drainage.

Saltwater crocodile samples (*n* = 1176) were collected between August 1997 and September 2021 across ten of twelve crocodile bioregions in the Queensland study area. Samples collected between August 1997 and October 2005 were sourced from the Queensland Museum (*n* = 482) and the remainder collected by Queensland Department of Science and Environment staff between May 2018 and July 2021 (*n* = 694). The number of samples taken from each bioregion comprises Fitzroy (*n* = 23), Coastal Plains: Ayr – Proserpine – Rockhampton (APrR) (*n* = 105), Coastal Plains: Cooktown – Ayr (CA) (*n* = 255), Coastal Plains: Cape Melville – Cooktown (CMC) (*n* = 2), Princess Charlotte Bay (*n* = 239), North East Cape York (*n* = 12), North West Cape York (*n* = 406), Gulf Plains: Mitchell‐Gilbert drainage (MGD) (*n* = 13), Gulf Plains: Norman‐Flinders drainage (NFD) (*n* = 111), and Gulf Plains: Albert‐Leichardt drainage (ALD) (*n* = 8). Figure 1 and Table S1 detail further the partitioning of samples across these bioregions.

As has been done historically in Australia, sampled crocodiles were assigned to 16 size classes based on total length (TL) (Fukuda et al., 2013) (see Table S2 for mapping between total length and length class). Samples ranged across all size classes with 861 (of 997 with size class information) <1.8 m (Table S3). Sex of 431 captured crocodiles (157 females and 274 males) was determined by cloacal examination. Sex was generally not determined for animals sampled by harpoon biopsy but seven large individuals were assumed male based on size, given females rarely exceed 3.5 m (Fukuda et al., 2013).

Tissue was sampled from free‐swimming crocodiles >600 mm TL using a non‐lethal biopsy punch method (Barrow & Halford, 2019). This involved a needle head (3 mm × 25 mm) modified to fit the end of a 3 m long Rangoon cane pole that was plunged into the base of the crocodile's tail from a moving boat at night. For smaller crocodiles (<600 mm TL) a single piece of tail scute was removed. Genetic material (using both methods) was also collected from problem crocodiles by the Department of Environment and Science (DES) during routine management activities required by departmental guidelines (Queensland Department of Environment and Science, 2008) under the Nature Conservation Act 1992. All genetic material was stored in 70% ethanol. Sampling was conducted under the Department of Agriculture and Fisheries Animal Ethics Committee Reference numbers SA 2018‐07‐644, and SA 2021‐07‐793. All activities were undertaken in accordance with the Code of Practice on the Humane Treatment of Wild and Farmed Australian Crocodiles (Natural Resource Management Ministerial Council, 2009).

### 2.3 SNP genotyping

#### 2.3.1 Pilot sample DArTSeq genotyping and marker selection for DArTcap

To minimise ascertainment bias, 188 crocodiles from within five of the most sample abundant bioregions were included in the SNP discovery phase. Specifically, 41, 39, 38, 31 and 39 samples were taken from Cairns, Lakefield, Norman River, Proserpine and Wenlock regions respectively (Table S1). DNA extraction and quality control was performed by Diversity Arrays Technology (DArT Pty Ltd) and samples were genotyped using DArTseq™ as described by (Grewe et al., 2015). Briefly, DArTseq combines organism‐tailored complexity reduction methods and next‐generation (NGS) sequencing platforms. For crocodiles, SNP calling was performed using DArT P/L's proprietary SNP and SilicoDArT calling algorithms (DArTsoft14). The process used the PstI – SphI restriction enzyme library protocol as used in Fukuda et al. (2019). The DArTseq analysis reported 15,514 pre‐scored markers (0, 1, 2 copies of reference allele and null) from 186 individuals (two failed internal quality control).

To shortlist DArTSeq markers from the pilot study to take forward to DArTcap genotyping, we further analysed DArT's reported clusters of sequences, of which there were 35,344. Using custom software written in the R programming language (R Core Team, 2021) we applied a quality control process to the cluster sequences. Clusters with median total read counts lower than 15 for likely heterozygotes were eliminated, removing 24,968 clusters. We assessed excessive polyploidy in both crocodiles (for contamination) and clusters (potential paralogous loci), which eliminated 288 clusters and no individuals. For each of the remaining clusters, the most numerous allele (in terms of total read counts across individuals) was identified and placed as the reference and counts for clusters with more than two alleles stored for downstream summary. For each of the two most numerous alleles (in terms of read counts) at a locus, the ratio of the median read counts in clear heterozygotes (at least 5% of the total proportion of counts at a locus for an individual were from the alternative allele) was compared for substantial deviation from 0.5. Loci were removed if they had values for this ratio of <0.33 or >0.67 (5004 clusters eliminated). These thresholds were determined from visual inspection of the distribution of the ratios across all loci and were chosen to balance the retention of high‐quality loci with removal of likely unreliable loci. The distribution across the remaining individuals of a composite score based on the total number of heterozygous loci minus the total number of null loci was assessed. A total of 9 crocodiles were removed due to outlier scores on the lower tail of this composite statistic suggesting excessive null rates for these individuals relative to the distribution across individuals. No outliers were removed on the upper tail of this distribution. Each locus for each crocodile was then scored based on whether one or both of the main alleles (labelled A and B) were present. All loci were genotyped to one of the four categories: AB; AA/A0; BB/B0; or 00 (double null), where A0 and B0 are individuals that contain a copy of the A or B allele on one chromosome but returned a null from the other (see Hillary et al. (2018) for further details). This four‐way scoring was applied to 5084 loci across 177 individuals. Post scoring, allele frequencies were computed and a minor allele frequency threshold of 0.02 was applied leaving 4799 loci.

To investigate the potential for population structure within these pilot data we performed a principal components analysis using the dudi.pca function from the ade4 R package (Bougeard & Dray, 2018) with no further quality control. A scatterplot of the first two principal components showed substantial sub‐population differentiation across the five pilot regions sampled (Figure S1). Given this substantial structure, SNP loci were not filtered for deviation from Hardy–Weinberg equilibrium (HWE) (via chi‐square test) because loci differentiated across sub‐populations were of interest in the large‐scale analysis. PCA analysis on a data set with a mild filter on HWE showed a similar pattern of differentiation on the PCA scatterplot (results not shown). As a final check, we separated the original cluster count data sets into bioregion‐specific data sets. We then followed the same quality control procedure as above and intersected the final quality‐controlled marker sets with the 4799 loci selected to assess if there was enough variation present in each bioregion. On average approximately 3200 markers intersected the 4799 in each bioregion‐specific sample, with the smallest number being in the Proserpine (2412 markers). The potential for more than 2000 high‐quality markers in any subpopulation was deemed adequate to perform the subsequent downstream analysis. We note that the results of these intersections may be confounded by the small sample sizes being used to determine quality control thresholds in the quality control pipeline. The set of 4799 short‐listed DArTSeq markers was deemed adequate and taken forward for DArTcap analysis.

#### 2.3.2 DArTcap genotyping

DArTcap genotyping involves adding a hybridisation‐based enrichment step before the DArTseq libraries are sequenced, which dramatically lowers the cost per sample when sample sizes are large. The hybridisation step uses custom synthesised biotinylated RNA MYbaits (Arbor Bioscience) designed based on the chosen DArTseq markers. The 4799 DArTSeq markers short‐listed for the DArTcap panel were put through a selection process using a DArT P/L proprietary algorithm that assesses sequence length and complexity to limit nonspecific capture. Markers with known sequences shorter than 40 bp were removed, as were those with low complexity. Sequence complexity was assessed by calculating a score based on median levels of guanine‐cytosine, the number of sequence variants at the locus, and length of homopolymers. This reduced the number of sequences for enrichment and one bait was designed based on the sequence of the most common allele at each locus. DArTcap hybridisation and washing used the protocols based on Version 3 of the MYBaits manual (https://arborbiosci.com/wpcontent/uploads/2017/10/MYbaits‐manual‐v3.pdf). These DArTcap enriched libraries, one per sample, were sequenced on an Illumina HiSeq. SNP calling for DArT reporting was again performed using DArT P/L's proprietary SNP‐calling algorithms. DArT P/L reported 6754 variants from 1176 individuals (of an original set of 1204 samples, 28 failed the internal quality control protocols at DArT). Of the original set of requested loci from DArTSeq selection approximately 43% were present in the reported DArTcap dataset.

#### 2.3.3 Quality control filtering of individuals and SNPs

Initial quality control of the SNP data was carried out before population genetic and kinship analyses. From the DArTcap procedure, we used DArT's two‐row counts format, which reports the number of sequence tags for the reference and alternate alleles for each SNP and is more informative than the pre‐scored SNP values. We performed quality control using the filter_rad pipeline in the radiator R package (Gosselin, 2020). The process filtered out both poor‐quality SNPs and poor‐quality individual samples that could have low DNA quality/quantity or be contaminated. See Table S4 for a detailed breakdown of individuals and SNPs filtered at each of the following quality control steps.

Quality control filters were set based on thresholds derived by visual inspection of quality control figures, outlier statistics and standard protocols. The SNP filtering steps included: reproducibility via outlier statistics (proportion of repeatable genotype calls estimated using technical replicates); call rate at 0.3 (proportion of samples genotyped); minor allele count 5 (number of times the allele in lowest frequency was observed); minimum and maximum average coverage (10, 200), a high coverage filter was used because this may suggest duplicated genome elements; retaining the SNP on each fragment with the highest minor allele counts (most informative); SNPs out of Hardy Weinberg equilibrium in at least three sampling populations at chi‐squared exact test threshold *p* < 1 × 10^−3^ as a final restricted filter to screen for variants of poor quality (total 42 variants removed).

To filter individuals, the following steps were performed; proportion of missing genotypes for an individual via outlier statistic; individual heterozygosity was filtered for only high outliers (>0.36) initially because some bioregion samples may be subject to inbreeding or other biological reasons for low heterozygosity. Individuals with low heterozygosity from the Fitzroy and Coastal Plains – CA bioregions showed distinctive separation either from the populations within their bioregions for the Coastal Plains – CA and from all other bioregions for the Fitzroy (see Figure S2 for detailed by‐bioregion heterozygosity). Other subpopulations with outlier low heterozygosity individuals (North West Cape York, Princess Charlotte Bay and Coastal Plain – AprR) showed evidence for a correlation between heterozygosity score and missing proportion, indicating poor DNA quality or sequencing effort bias, and were therefore removed (Figure S2). The low heterozygosity individuals (median value of 0.159) from the Coastal Plains – CA bioregion comprised 46 individuals, which contained five large (>2.7 m) male individuals sampled in 2018 from Boar Creek, Tully, and 40 museum samples from 1998/99 containing mostly juveniles recorded as sampled from the Edmund Kennedy National Park. Pilot PCA scatter plots using the quality‐controlled data (to this point in the pipeline) with these individuals included showed clear separation from other members of the Coastal Plains – CA bioregion (Figure S3). For these individuals there was no correlation between heterozygosity score and missing proportion and further checks of sample and laboratory metadata indicated that the DNA for these individuals was of adequate quality. Despite this, we chose to exclude these individuals from the population genetic analyses based on their low heterozygosity values and their sharp divergence from other individuals in the Coastal Plains – CA bioregion. Understanding why these Coastal Plains – CA samples had very low heterozygosity is outside the scope of this study and is difficult to resolve due to the uncertainty in reported sample locations for the museum samples. Individuals with low heterozygosity from the Fitzroy were not excluded from the analysis as they were at the edge of the species' range and could show low heterozygosity for biological reasons. Individuals that showed evidence of duplication were also removed (one individual from each pair discarded with the individual with the lowest rate of missingness kept).

Following these filters, 948 individuals and 2958 variants were available for analysis. Of these 2958 variants approximately 49% were present in the selected loci set from the SNP discovery. For the bioregion Coastal Plains: Cape Melville – Cooktown, only two individuals were sampled. For analyses that summarise at a bioregion scale, these individuals were amalgamated with the closest spatial group, which was Princess Charlotte Bay. Initial pilot principal component analyses showed that these two individuals did group genetically with the Princess Charlotte Bay bioregion.

### 2.4 Population diversity and structure analyses

Initial genetic diversity statistics were computed using the R package diveRsity (Keenan et al., 2013) for each bioregion and included allelic richness (*A*_*r*_), observed heterozygosity (*H*_*O*_), unbiased expected heterozygosity (*H*_*E*_) and inbreeding coefficients (*F*_IS_). We used the fixation index (*F*_ST_) as a measure of population genetic differentiation calculated using the R package StAMPP (Pembleton et al., 2013) for all pairwise combinations of bioregions. The StAMPP bootstrap (1000 replicates) *p*‐values for the pairwise *F*_ST_ values were adjusted for multiple comparisons using a false discovery rate (Benjamini & Hochberg, 1995) at 5%.

We investigated the relationship between genetic and geographic distances by plotting Slatkin's linearised *F*_ST_ (generated from StAMPP and transformed using *F*_ST_/(1−*F*_ST_) (Rousset, 1997)) as a measure of genetic differentiation. Geographic distance was measured as the coastal distance between bioregion centroids. The coastal distance was computed using a spatial layer of the Queensland coastline designed for crocodile reporting elsewhere (Taplin et al., 2020). The bioregion centroids were matched to their closest coastal line position and the distance computed along the coastline. We tested for a correlation between genetic and geographic distances using the standard Mantel test implemented in the R package vegan (Oksanen et al., 2020).

To assess the partitioning of broad‐scale genetic variation, we conducted principal component analyses (PCA) using the R package adegenet (Jombart & Ahmed, 2011) and data from 948 individuals and 2958 variants. We further filtered these data on linkage disequilibrium at *R* ^2^ = 0.1 (using the snpgdsLDpruning function in the SNPRelate R package (Zheng et al., 2012)) and a sliding window of 3000 to include all variants, which left 1708 variants for PCA. We investigated the influence of unequal sample sizes on PCA inference, which could introduce bias in clustering algorithms (Foster et al., 2018), by down‐sampling bioregions with large numbers of individuals to 30 individuals.

We examined the correspondence between the primary axes of genetic variation from the principal components analyses and geography using the Procrustes transformation of the first two principal components using the MCMCpack package in R (Martin et al., 2011). Procrustes transformations scale, stretch, and rotate the PCA axes to minimize the differences between the principal components and the geographic coordinates of each of the saltwater crocodile samples.

To investigate individual genetic ancestry partitioning, we used a model‐based clustering approach implemented in STRUCTURE (Pritchard et al., 2000). We ran STRUCTURE for 10 independent runs across clusters values *K* = (2, …, 10), and investigated model fit for these *K* values across aligned model runs with the Delta K method (Evanno et al., 2005) implemented in the pophelper (Francis, 2017) R package. We used 20,000 burn‐in Markov Chain Monte Carlo iterations followed by 80,000 further iterations for inference. We ensured the adequacy of the run length by checking the likelihood and alpha parameter for stability at longer MCMC chain lengths. We assumed that allele frequencies were correlated between sampled sites and allowed for admixture for all runs. All runs were completed with and without prior location information. The location prior information was set to the sampled bioregion for each individual.

Finally, to complement the principal component analysis, we performed a Discriminant Analysis of Principal Components (DAPC), implemented in the adegenet R package. For DAPC, we again filtered on linkage disequilibrium as per the PCA. In the first DAPC analysis, we used k‐means (percentage variance method – 95%) to identify clusters in the data and selected the cluster number that best describes the variance in the data set via inspection of the Bayesian Information Criterion as a function of the number of clusters. In the second DAPC analysis, a priori grouping based on sampled bioregions was investigated. Cross‐validation, with 30 replicates and a 90/10 ratio for the training/validation sets, was used as an optimization procedure to select the adequate number of principal components to retain in the discriminant analysis.

### 2.5 Kin identification

Identification of close relatives can provide a direct estimate of connectivity over shorter timescales (one or two generations), as opposed to long timescales (hundreds or thousands of generations) with population genetics. The timescale at which inference is made depends on the relatedness degree of the kin observed. Here, we focus on making inferences on recent connectivity using close‐kin, which for our study include parent‐offspring pairs (POPs), full‐ and half‐sibling pairs F/HSP. The integration of classical population genetics with knowledge from close kin provides an approach to study the current and distant connectivity of a species (Feutry et al., 2017, 2020).

### 2.6 PC‐relate

PC‐Relate (Conomos et al., 2016) is a model‐free approach for estimating commonly used measures of recent genetic relatedness, such as kinship coefficients and identity by descent (IBD) sharing probabilities, in the presence of unspecified structure. The PC‐Relate method is very well suited to the problem of inferring kin in structured populations and distinguishes familial relatedness from population structure, which both manifest as genetic similarity through the sharing of alleles. The method was pioneered in humans and applied to data sets with large numbers of SNPs. We explored PC‐Relate's utility for differentiating parent‐offspring pairs (POPs), full‐sibling pairs (FSPs) and 2*nd*‐degree relatives including half‐sibling pairs, uncle/aunt – niece/nephew (referred to as full‐thiatic pairs (FTPs) for brevity) and grandparent/grandchild pairs (GGPs) from unrelated or undetermined pairs (U).

### 2.7 SLiM forward simulation

To calibrate our expectations for inferring kin using PC‐Relate from data sets with approximately a few thousand SNPs, which is typical in ecological applications and far fewer than in human studies, we implemented forward‐time non‐Wright–Fisher simulations using the SLiM 3 software package (Haller & Messer, 2019). This incorporated sub‐populations, adult migration and clutches, which are expected and observed properties in the *C. porosus* population. We simulated two sub‐populations linked by various migration rates at 1%, 0.1% and 0.01% per generation. The sub‐populations each had a carrying capacity of 1500 individuals. The mutation rate was set at 3.5 × 10^−9^ per base pair per generation and the crossover rate at 10^−8^ per base pair per generation, which is equivalent to per one centimorgan per megabase. The mortality rate was set to be proportional to the ratio of the carrying capacity (1500) to the number of individuals in the sub‐population at the current time step. The simulation structure led to the oldest individual in the population being generally between 50 and 80 years old, with most of the population comprising juveniles of *<*10 years old. Reproduction is stipulated by each female 15 years or older randomly mating with a male from the same sub‐population, also 16 years or older (age at sexual maturity). There was no sex‐ratio bias in offspring and individuals within each sub‐population were picked at random to be migrants. The simulations were run for 6000 generations, with pedigree tracking for the last 100 generations.

We repeated these three simulation scenarios with each female having an expected clutch size of *λ* = 15 offspring drawn from a Poisson distribution. Brien et al. (2014) estimated that on average saltwater crocodile clutch sizes were ≈55 eggs with more than half of the individuals surviving to 1 year of age. Alternatively, it has been estimated that only 30% of *C. porosus* eggs yield hatchlings in the wild (Webb & Manolis, 1993). As juveniles in the simulation are not subjected to any other mortality pressure relative to the rest of the population, we chose the expectation to be 15 offspring, which is slightly less than the 30% on‐average estimate making it to 1 year old in the wild.

For each of the six simulation scenarios, we sampled 50 replicates with a sample size of 500 individuals (250 from each subpopulation) and calculated PC‐Relate kinship statistics for each of the sampled populations using the GENESIS R package (Gogarten et al., 2019). To validate the outcomes from PC‐Relate, functions were written in the R programming language to parse the true pedigree information from SLiM output and identify kin pairs that were parent‐offspring, full‐siblings, half‐siblings, grandparent‐grandchild and full‐thiatic pairs. Samples were picked from the living individuals in the last generation of each of the six simulation scenarios. PCA analysis on the generated populations was performed to gauge the level of structure in these simulated populations and implemented using the adegenet R package. For each sample, we performed PC‐Relate‐specific quality control of filtering variants at MAF 5% and linkage disequilibrium at *R* ^2^ at 0.1 as in Conomos et al. (2016).

To cluster individual pairs into kinship categories we explored three methods including a Euclidean distance clustering method and a support vector machine (SVM). Conomos et al. (2016) determined kin by clustering based on the criteria given in Manichaikul et al. (2010) (hereafter referred to as the Manichaikul criteria), which was the third method. The criteria classify pairs of individuals to have a *d*th degree relationship if their estimated kinship coefficient (*φ*) is in the interval (2^−(*d* + 3/2)^, 2^−(*d* + 1/2)^). To distinguish parent‐offspring from full sibling relationships, pairs with a *k*(0) estimate <2^−(9/2)^ ≈ 0.044 are classified as parent‐offspring. Given the greater expected variability in kinship statistics due to close relatives and the small number of variants, relative to Conomos et al. (2016), available for analysis, we chose to compare alternative clustering methods with the Manichaikul criteria.

For the Euclidean distance clustering method, we clustered points in three kinship statistic dimensions including the estimated kinship coefficient (φ), estimated probabilities of sharing zero alleles IBD *k*(0), and the probabilities of sharing two alleles IBD, *k*(2). The clustering assigns each pair to a kinship category that has the closest Euclidean distance to the expected kinship statistic centres. The centres used were POP = (0.25, 0, 0), FSP = (0.25, 0.25, 0.25), 2nd degree = (0.125, 0.5, 0), 3rd degree = (0.063, 0.75, 0) and unrelated (0, 1, 0), which correspond to the expectations of the kinship coefficient, *k*(0) and *k*(2) statistics for each of these kinship classes. For each pair, the Euclidean distance of the pair's point in the three kinship statistic dimensions to all centres is computed and the pair is assigned to the kinship class with the smallest distance. For the SVM classification, we took the kinship statistics from the first five simulation replicates in each of the six simulation scenarios (≈ 250,000 points) and trained an SVM with the svm function and a linear kernel in the e1071 R package. We used the trained SVM to classify the kinship pairs in the three kinship statistic dimensions in the remaining 45 simulation replicates that were not included in the training set.

We used multiple clustering performance measures to compare the methods and gauge expectations for the saltwater crocodile data set. We computed the precision TP/(TP + FP), recall TP/(TP + FN), specificity TN/(TN + FP), and F1_macro_ = 2(P_macro_ ×R_macro_)/(P_macro_ + R_macro_), where TP is true positive, FP is false positive, FN is false negative and P_macro_ and R_macro_ are the macro precision and recall. The F1_macro_ averages over the performance for individual kin classes and if it has a score close to unity indicates that the classifier performs well for each class. These measures were computed and summarised for the three methods across the 45 replicates used for testing from ‘confusion matrices’ computed using the caret R package (Kuhn, 2015).

### 2.8 Saltwater crocodiles

To perform model‐free estimation of genetic relatedness for the Queensland saltwater crocodile population we used the PC‐Relate method implemented in the GENESIS R package. We used the low‐heterozygosity quality‐controlled data that had 948 individuals and 2958 SNPs. We filtered these data on linkage disequilibrium at *R* ^2^ = 0.1 and a sliding window of 3000 to include all variants. A further MAF filter at 5% was then applied, which left 1628 variants for analysis. PC‐AiR analysis was then performed, which estimates principal components from SNP datasets and is not confounded by recent genetic relatedness. PC‐AiR requires pairwise kinship coefficients for every pair of individuals in the sample, which was estimated using the KING‐robust estimators implemented in the SNPRelate R package (Zheng et al., 2012). An adequate set of principal components estimated from the unrelated set of individuals from PC‐AiR was taken forward for PC‐Relate analysis. SNPs were filtered in the PC‐Relate function if an individual's estimated individual‐specific minor allele frequency was <0.01, and the set of unrelated individuals determined from PC‐AiR analysis was used for the training set in the PC‐Relate analysis, as recommended by Conomos et al. (2016). Kinship summaries were performed using the distribution of kinship coefficients (φ), estimated probabilities of sharing zero alleles IBD, *k*(0), and the probabilities of sharing two alleles IBD, *k*(2) returned from PC‐Relate. The distribution of φ was also plotted against coastal distance, where each crocodile's sampling coordinates was matched to the closest coastal position (from the Queensland coastline spatial layer used by (Taplin et al., 2020)) and distance computed between each pair of individuals.

Kinship assignment of pairs to POPs, FSPs and 2nd‐degree relatives was predicted from the PC‐Relate kinship statistics using the Manichaikul criteria and four SVM classifiers, which included the three trained on the simulated data with clutch breeding at the three migration rates and a final all‐scenario model, which was trained on five replicates of data from each of the three clutch scenarios (672,750 total points). The output from these four classifiers was then compared for concordance. Further verification of kinship status for pairs of high interest was performed using available demographic information including sex, body size, sampling date and location.

## 3 RESULTS

To examine genetic variation in the *C. porosus* population, we sampled 1,176 individuals distributed across 10 bioregions in Queensland. We obtained DArT SNP data for these individuals and after stringent filtering ≈3,000 SNPs and 948 individuals were analysed.

### 3.1 Genetic diversity and fixation index

Genetic diversity indices across nine bioregions were summarised and showed broad evidence for lower diversity for the bioregions at the edge of the species' range (Table 1). Monomorphic loci featured prominently in several bioregions, particularly in the Fitzroy population where close to one‐third were monomorphic, the Coastal Plains – APrR (16%) and the Gulf Plains – ALD bioregion (19%) (Table 1). These three regions also showed the lowest allelic richness scores.

**TABLE 1:** Genetic diversity indices by bioregion generated from 948 crocodiles.

	Gulf plains			Cape York		Princess Charlotte Bay	Coastal plains		
	ALD	NFD	MGD	North west	North east		CA	APrR	Fitzroy
*N*	7	104	13	342	11	208	169	77	17
*M*_0_	550	75	312	12	332	39	21	483	799
MAF < 1%	550	128	312	101	332	132	99	651	799
*A*_*r*_	1.570	1.660	1.620	1.660	1.620	1.660	1.650	1.520	1.410
*H*_*O*_	0.242	0.249	0.247	0.241	0.243	0.248	0.234	0.240	0.151
*uH*_*E*_	0.233	0.247	0.242	0.245	0.247	0.249	0.245	0.200	0.146
*F*_IS_	−0.110	−0.006	−0.058	0.021	−0.033	0.005	0.047	−0.147	−0.001
*F*_IS_ – CI	−0.251 to −0.109	−0.019 to −0.002	−0.136 to −0.057	0.013 to 0.024	−0.125 to −0.036	−0.004 to 0.009	0.031 to 0.054	−0.178 to −0.136	−0.201 to 0.034

*Note*: Genetic diversity indices for each of the nine bioregion populations, comprising 948 saltwater crocodiles and 2958 SNPs. Reported are the number of individuals within each bioregion (*N*), number of monomorphic loci (*M*_0_), number of loci with minor allele frequency (MAF) <1%, allelic richness (*A*_r_), observed heterozygosity (*H*_*O*_), unbiased expected heterozygosity (*uH*_*E*_), and inbreeding coefficient *F*_IS_ and 95% bootstrap (200 replicates) confidence intervals (CI).

Bioregion abbreviations: ALD, Albert‐Leichhardt drainage; APrR, Ayr – Proserpine – Rockhampton; CA, Cooktown – Ayr; MGD, Mitchell‐Gilbert drainage; NFD, Norman‐Flinders drainage.

Mean heterozygosity in the Fitzroy was approximately two‐thirds that of other populations, as expected from earlier quality control investigations (Table 1 and Figure S2). The Coastal Plains – APrR bioregion showed the largest difference between observed (0.240) and expected heterozygosity (0.200).

Inbreeding coefficients varied substantially across bioregions. The Gulf Plains – ALD, and Coastal Plains – APrR bioregions showed substantially negative *F*_IS_ values, while small negative values were found for the Gulf Plains – MGD, Gulf Plains – NFD and north‐east Cape York bioregions (Table 1). The Fitzroy bioregion also showed a negative *F*_IS_ but its confidence interval included zero; the CI for Fitzroy was the widest of all bioregions with a substantially negative lower interval (−0.201).

**FIGURE 2:**
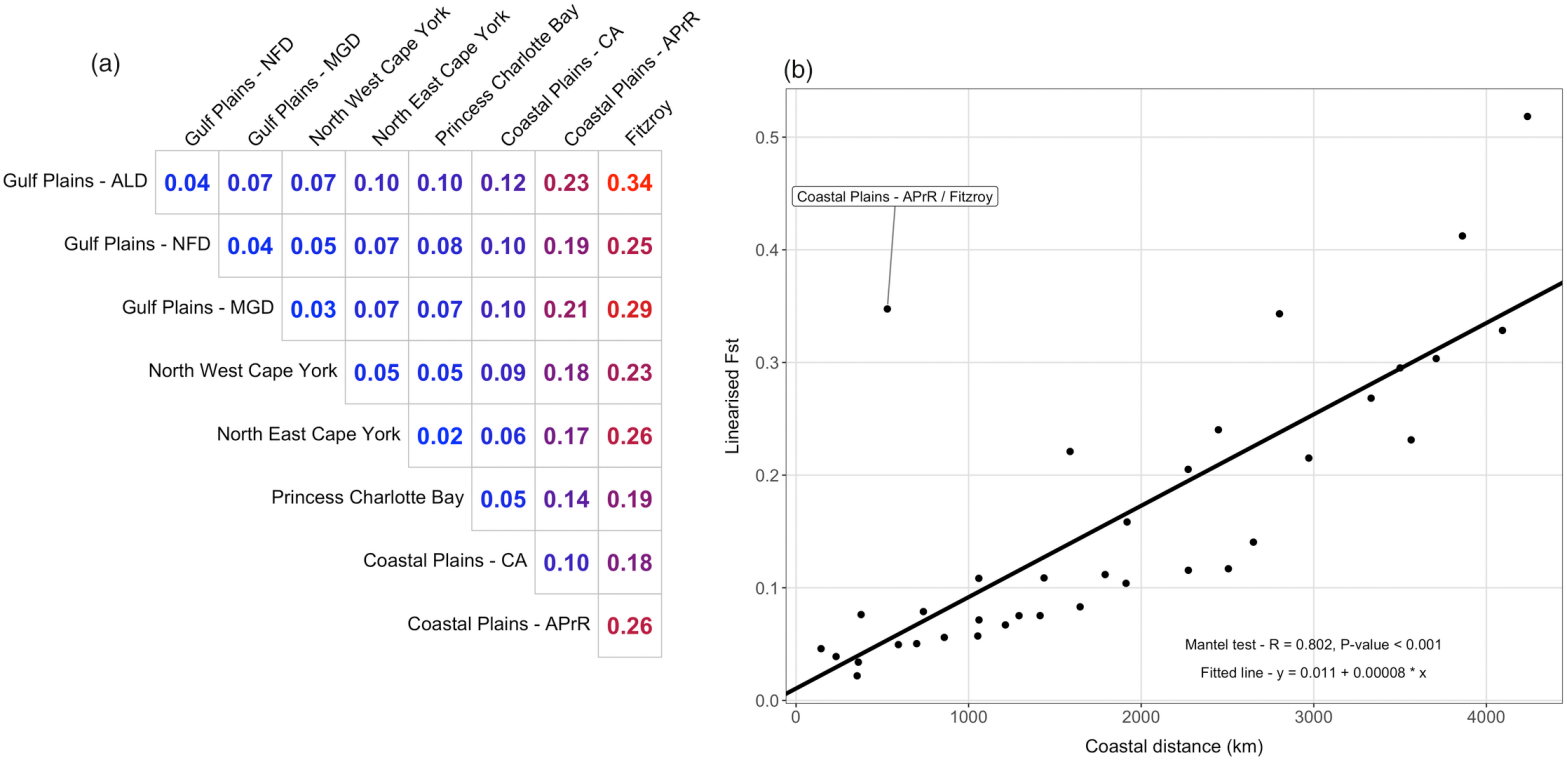
Pairwise *F*_ST_ values and correlation of genetic and geographic distance between all bioregions calculated from 948 crocodiles. All entries in a) are significant with bootstrap (replicates = 1000) *p*‐values less than the FDR adjusted threshold of 0.05. Row and columns in panel (a) are ordered by position along the Queensland coast from west to east. Bioregion abbreviations are ALD, Albert‐Leichhardt drainage; APrR, Ayr – Proserpine – Rockhampton; CA, Cooktown – Ayr; CMC, Cape Melville – Cooktown; MGD, Mitchell‐Gilbert drainage; NFD, Norman‐Flinders drainage. Panel (b) shows the positive correlation between coastal distance and Slatkin's linearized *F*_ST_. The black line indicates the fitted regression line with the coefficients detailed in the figure. The Mantel correlation coefficient and its associated significance are also shown. The point comparing the Coastal Plains – APrR and Fitzroy bioregions is highlighted.

Fixation indices ranged from 0.02 (between north‐east Cape York and Princess Charlotte Bay) to 0.34 (Gulf Plains – ALD and Fitzroy) with all *F*_ST_ values significant at a false discovery rate of 5% (Figure 2a). We observed that *F*_*ST*_ increases linearly with spatial distance along the coastline with the prominent exception of the Coastal Plains – APrR/Fitzroy pair (Figure 2b). Within western bioregion comparisons, *F*_ST_ values were smaller than within eastern bioregions with a maximum value of 0.07 in the west versus 0.26 in the east. The Mantel test between linearized *F*_ST_ and coastal distance revealed a significant strong positive correlation (*R* = 0.802, *p* < 0.001) suggesting an isolation‐by‐distance (IBD) effect for this population.

We investigated the sensitivity of these analyses to the data quality control filtering choices by generating a separate data set with no MAC filter and a HWE filter that requires the SNP to be out of HWE in all populations at a chi‐squared test *p* = 0.01 (1 SNP removed), which left 1052 individuals and 4313 SNPs (Table S5). Genetic diversity indices and *F*_ST_ values patterns were consistent with those reported above (Table S6 and Figure S4).

### 3.2 Population structure analysis

**FIGURE 3:**
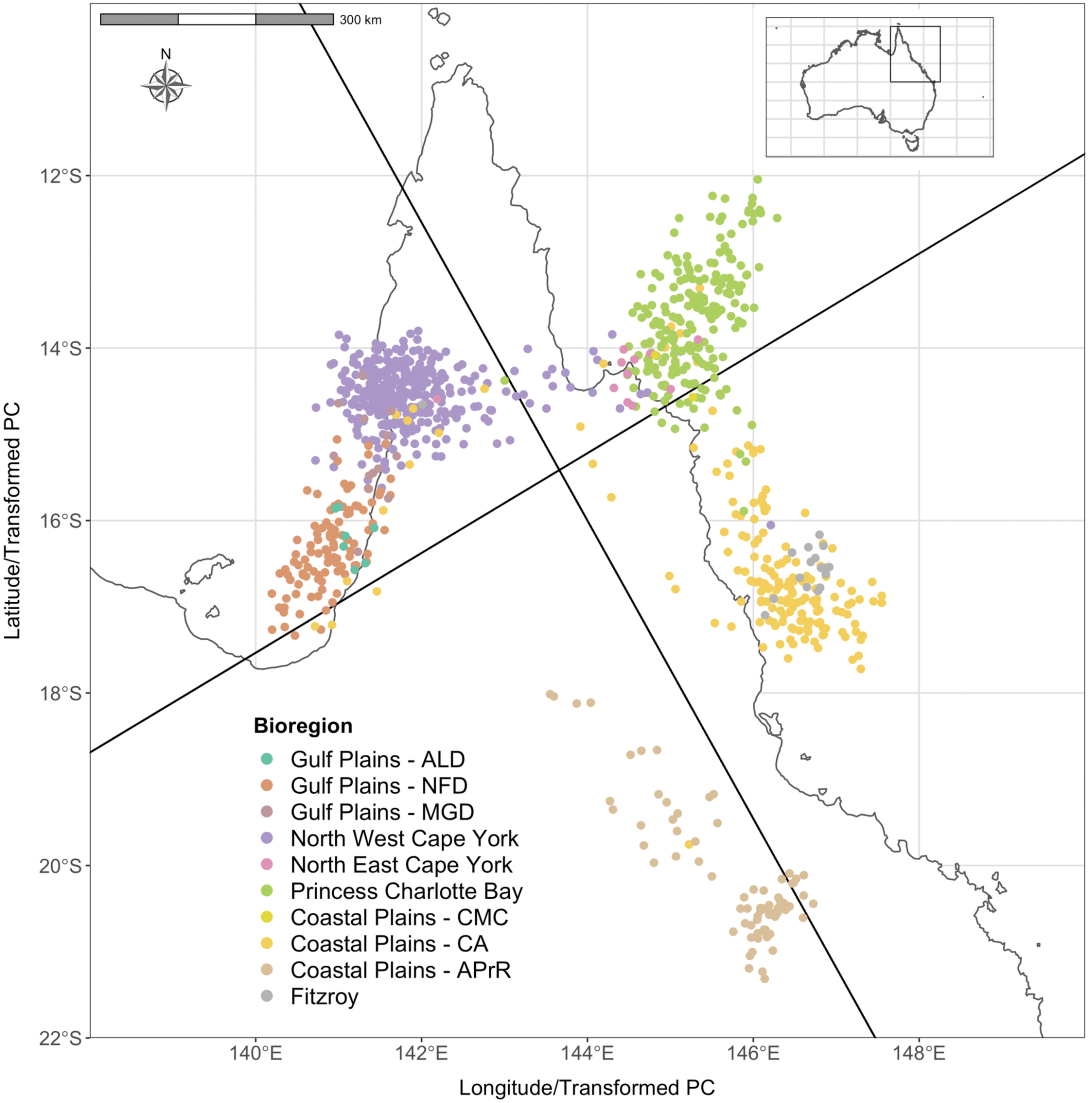
Procrustes transformation of PC 1 versus PC 2 onto geographic coordinates. The transformation indicates a strong correspondence between sampling location and genotype distance in principal component space. Bioregion abbreviations are ALD, Albert‐Leichhardt drainage; APrR, Ayr – Proserpine – Rockhampton; CA, Cooktown – Ayr; CMC, Cape Melville – Cooktown; MGD, Mitchell‐Gilbert drainage; NFD, Norman‐Flinders drainage.

Principal component analysis was performed to investigate structure in the genetic variation of individuals sampled across Queensland. PCA analysis on the 948 individuals and 1708 loci (LD filtered) showed substantial population structure across the bioregions, with PC1, PC2 and PC3 accounting for 4.4%, 2.3%, and 2.03% of the total variation present in the genetic data, respectively (Figures S5 and S6). PC1 delineated the eastern from western bioregions and PC2 the north from the south; the Coastal Plains – APrR bioregion was an exception. The down‐ sampled PCA showed a similar clustering of populations (Figure S7). There was evidence of migrant or translocated individuals (or their close relatives), particularly in the Coastal Plains – CA bioregion with the PCA plot showing individuals sampled from this bioregion that are genetically similar to the North West Cape York bioregion (Figure 3 and Figure S5). The Coastal Plains – APrR population showed a substantial extension on the PCA scatter plot that contained an individual from Coastal Plains – CA (sampled in Boar Creek, Tully in 2018), which may indicate admixture between populations. There were further PC projections between large clusters that are spatially proximal. Broadly, the genetic variation of the Queensland population was partitioned into five groups including Gulf Plains – ALD, MGD and NFD; North West Cape York; North East Cape York and Princess Charlotte Bay; Coastal Plains – CA, CMC, and Fitzroy, and the Coastal Plains – APrR. The PCA places the Fitzroy population with the Coastal Plain – CA bioregion, coinciding with the *F*_ST_ results. The Procrustes transformation of PC1 and PC2 revealed high correspondence between genetic differentiation and geographic distance with the Fitzroy bioregion being the least concordant (Figure 3).

**FIGURE 4:**
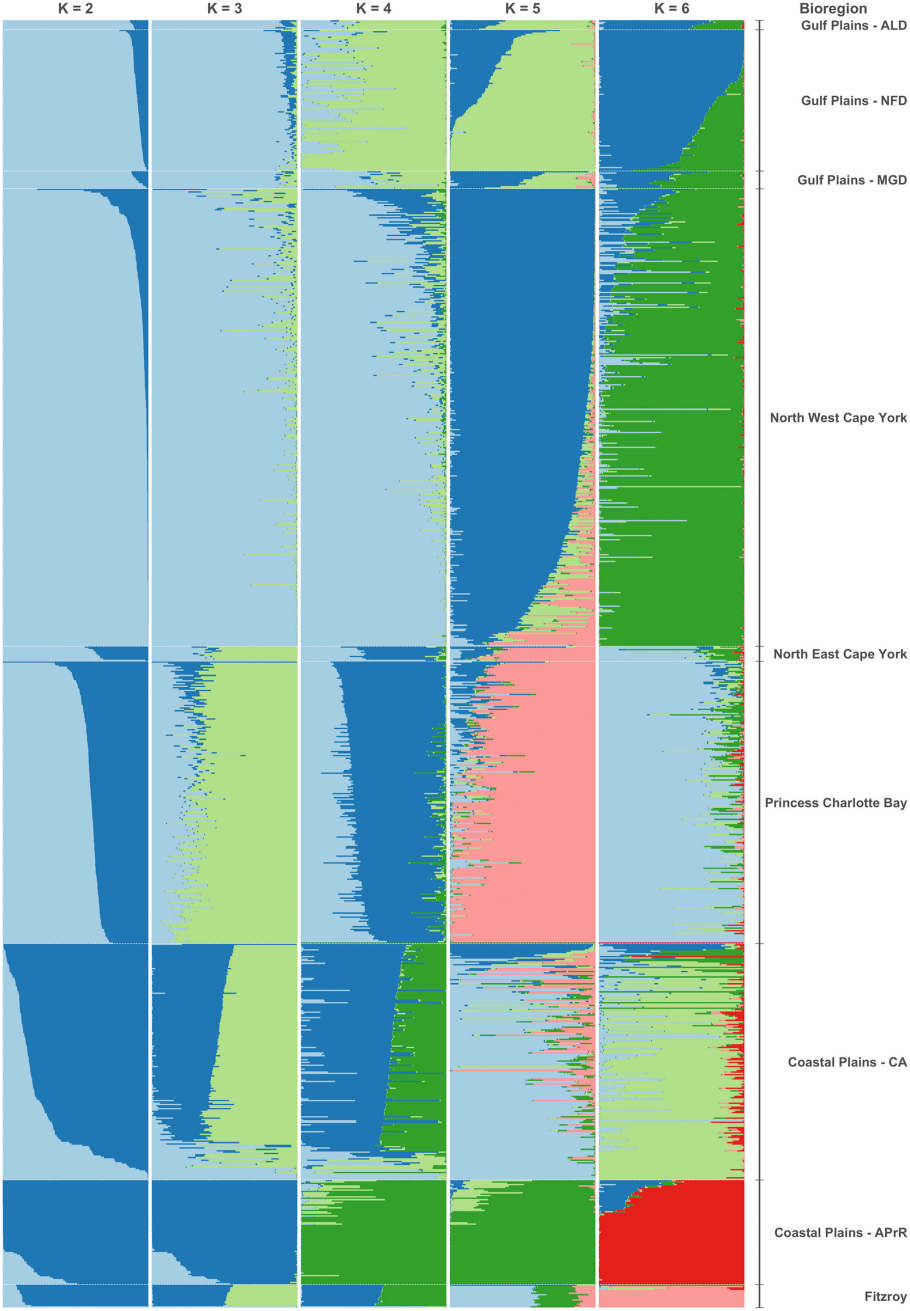
Admixture plots generated from STRUCTURE clustering analyses. Horizontal coloured lines represent an individual sample from the bioregion indicated on the right panel. Results are based on checked convergence from five replicate runs for each K. For each K, individuals are ordered within bioregion by their major ancestry proportion. Bioregion abbreviations are ALD, Albert‐Leichhardt drainage; APrR, Ayr – Proserpine – Rockhampton; CA, Cooktown – Ayr; MGD, Mitchell‐Gilbert drainage; NFD, Norman‐Flinders drainage.

Both STRUCTURE and DAPC analyses of the full data set (without a priori information on sampling location) showed evidence that five populations partition the genetic variance of the population well, corroborating the observations made from the PCA scatterplot. For STRUCTURE, the log probability of the data remained consistent between 20,000 iterations and 80,000 MCMC iterations (Figures S8–S10). We, therefore, reported the results from the 80,000‐iteration run. The ∆K criterion (Evanno et al., 2005) from the analysis using up to 10 groups, showed equal weighting between three to five clusters with a drop in the rate of increase of the log probability of the data and an increase in variance for *K* = 6. When the location prior was used, the ∆K criterion was equal up to *K* = 7 with an elbow in the rate of change of the log probability of the data at *K* = 5. The broad variation in groups and across individuals and regions remained similar between the no‐prior and prior information runs with similar groupings and log‐probability of the data values (Figures S11 and S12). When *K* = 2, western populations showed different ancestral proportion patterns to the eastern bioregion individuals with the Princess Charlotte Bay and North East Cape York populations being intermediate. For higher values of *K*, the North West Cape York group differentiates from the other western populations and, concurrently, the Coastal Plains – APrR and Fitzroy group differentiate from each other in the east. The Fitzroy population showed similar ancestral populations to the Coastal Plains – CA bioregion until *K* = 6, where they differentiated into their own group. Again, there is evidence for a set of individuals in the Coastal Plains CA bioregion originating from the Gulf Plains and North West Cape York sub‐populations (Figure 4).

**FIGURE 5:**
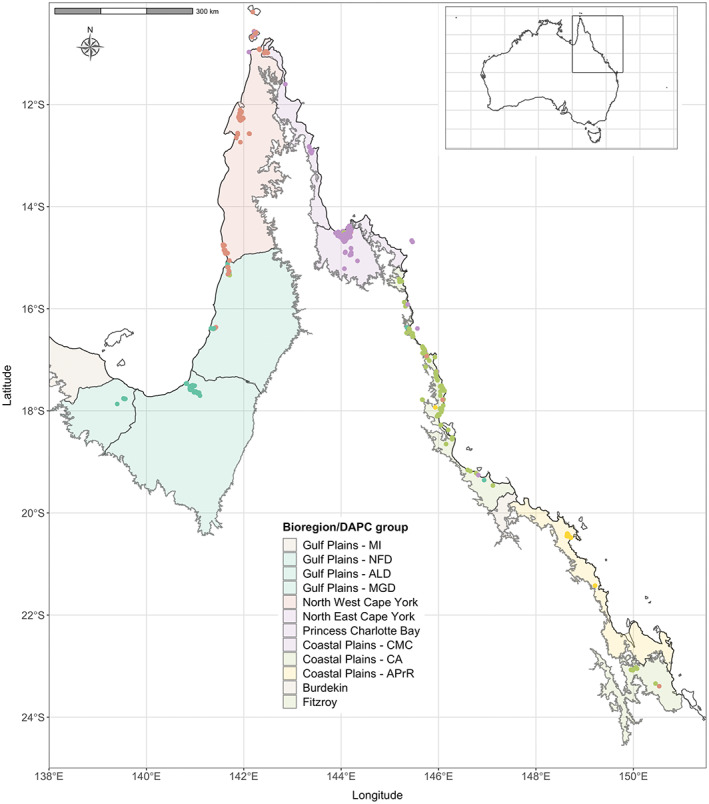
Map of samples differentiated in to five clusters from the DAPC with k‐means clustering analysis of saltwater crocodile genetic data. Points correspond to sample instances of individual crocodiles and are coloured by the five clusters determined from the DAPC analysis (see Figure S14). Bioregions are coloured to reflect the five clusters with the colour of each bioregion corresponding to the group that has the highest number of individuals of a DAPC cluster in that bioregion. The Gulf Plain – MI and Burdekin bioregions are presented for completeness and are coloured but were not included in the analysis. Bioregion abbreviations are ALD, Albert‐Leichhardt drainage; APrR, Ayr – Proserpine – Rockhampton; CA, Cooktown – Ayr; CMC, Cape Melville – Cooktown; MI, Massacre Inlet; MGD, Mitchell‐Gilbert drainage; NFD, Norman‐Flinders drainage.

The DAPC BIC investigation for determining clusters showed the first elbow at *K* = 5 with a minimum at *K* = 8 (Figure S13). Using 50 PCs determined from the cross‐validation analysis (Figure S14), the discriminant analysis indicated five groups with the western bioregions closely grouped and three distinct eastern groups (Figure 5, Figure S15 and Table S7). For the three eastern clusters, the Coastal Plains – APrR bioregion showed the most differentiation with the two other clusters predominantly made up of the Princess Charlotte Bay combined with North East Cape York, and the Coastal Plains – CA bioregion. DAPC discriminant analysis for 8 clusters showed a further partitioning of the North West Cape York, Princess Charlotte Bay and Coastal Plains – CA bioregions (Figures S18 and S19 and Table S8). The DAPC analysis showed the classification of across‐bioregion individuals with an individual sampled in the Fitzroy grouped (with near 1 posterior probability) with individuals predominantly from the North West Cape York. An individual from each of the Princess Charlotte Bay and Coastal Plains – CA bioregions was clustered with individuals from the Coastal Plains – APrR, which is consistent with the PCA observations and may contribute to an explanation of the extension (discussed above) between these bioregions. Individuals were grouped in the Princess Charlotte Bay (*N* = 18) and North West Cape York (*N* = 6) bioregions, but were sampled in the Coastal Plains – CA bioregion (Table S8).

### 3.3 Kin identification

#### 3.3.1 SLiM forward simulation

For each of the six forward simulation scenarios, the PCA analysis showed that the per‐generation migration rates chosen (1%, 0.1%, 0.01%) spanned the full range of no sub‐population separation to near full sub‐population separation (Figures S20 and S21). As expected, the PC‐Relate kinship statistics were stable in terms of means and variances across sub‐population differentiation scenarios (Tables S9 and S10) with some stable inflation in φ over replicates for 2nd‐degree relatives, which included all HSPs, FTPs and GGPs. The biggest qualitative difference between the no‐clutch and clutch simulation scenarios is the dramatic increase in FSPs and FTPs, which is expected. GGPs were more prevalent in the no‐clutch simulations with FSPs appearing rarely. Therefore, we ignored summaries of FSPs clustering in the no‐clutch simulations. Across scenarios, the *F*1_macro_ was 0.861 for the SVM, 0.698 for the Euclidean clustering, and 0.720 using the Manichaikul criteria (Figure S22). The *F*1_macro_ was higher in the clutch simulations than the no‐clutch scenarios owing to the increased performance on FSP clustering (Figure S22). Although the Euclidean clustering performed reasonably well it had poor precision for POP classification, clustering pairs with a *k*(0) away from 0 as POPs (see Figure S23 for a representative example). If we assume a clutch scenario and that the SVM clustering translates to real data then we can expect the false‐discovery rate (1−precision) to be <5% for POPs, ≈10% for FSPs and ≈20% for 2nd‐degree relatives (Figure S22).

#### 3.3.2 Saltwater crocodiles

We estimated recent genetic relatedness in the saltwater crocodile population using the PC‐Relate method. KING‐robust estimators of kinship for all pairs generated from the sample were used for PC‐AiR analysis and showed the distortion in the expected distribution of these statistics due to structure in the crocodile population (Figure S24). The first two PCs from PC‐AiR showed the differentiation of the population and classified the population into 213 unrelated and 736 related individuals (Figure S25). The first 10 PCs exceeded the elbow in the PC proportion of the variance plot and were taken forward for PC‐Relate analysis (Figure S25). PC‐Relate estimates of the kinship coefficient *φ*, *k*(0), and *k*(2) were obtained for 448,878 pairwise comparisons. Initial summaries of the distribution in the kinship statistics show the correction for structure compared to the KING‐robust estimates (Figure S26).

**FIGURE 6:**
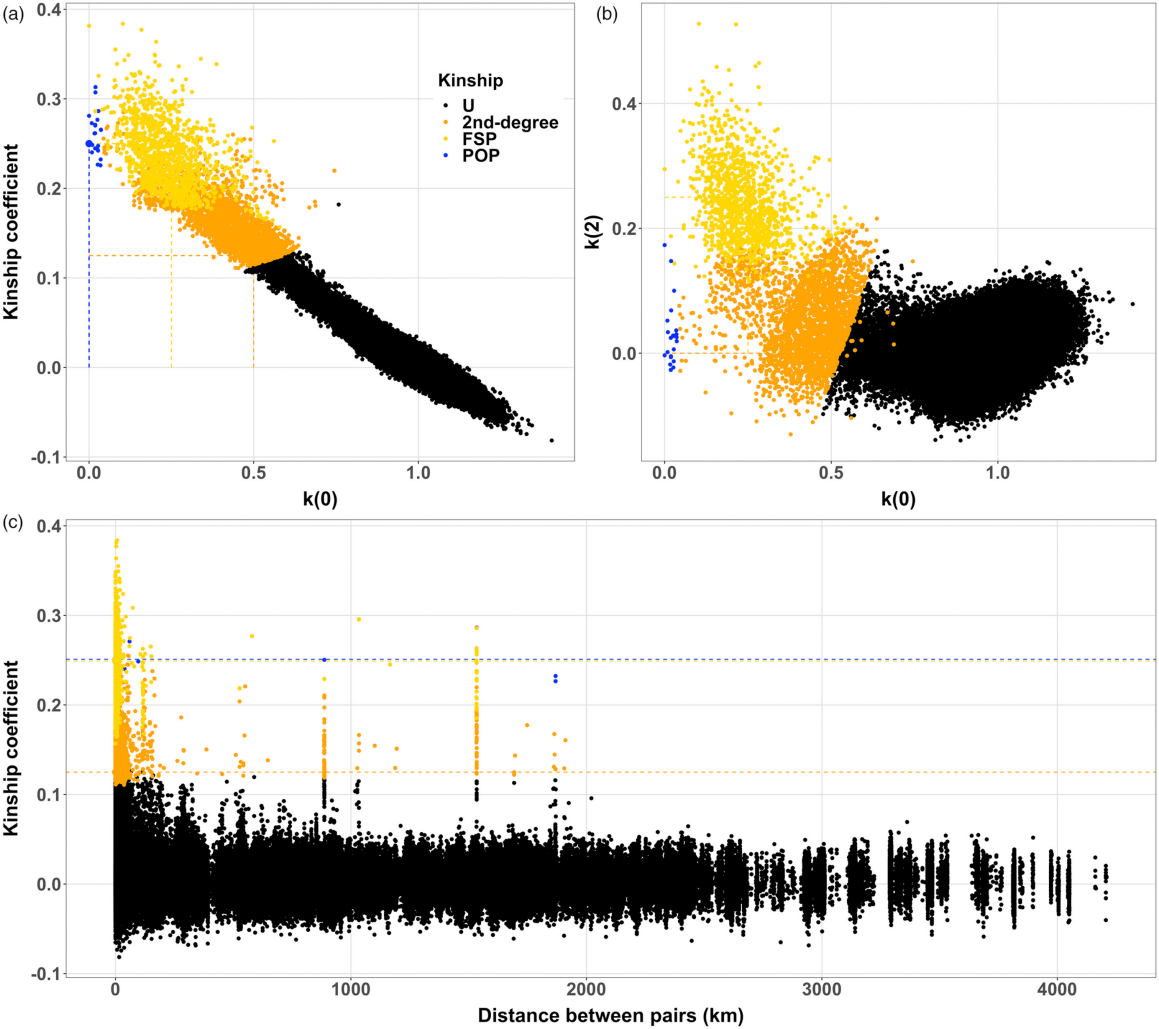
Scatterplots of the kinship coefficients and statistics from PC‐Relate analysis of saltwater crocodile population. Panels (a, b) show the kinship coefficient, *k*(0) and *k*(2) distribution estimate for all pairs from the PC‐Relate analysis and the classification into parent‐offspring (POP), full‐sibling (FSP) and 2nd‐degree relatives including potentially half‐sibling, grandparent/grandchild and full‐thiatic pairs. The category U encompasses all pairs that were not classified as first‐ or second‐degree relatives. Dashed lines show the expected values for each of the statistics. Panel (c) displays the scatterplot of the kinship coefficient versus the geographic distance between each pair along with the classification for each pair. The geographic distance is the distance along the coastline between pairs in different bioregions. For pairs within a bioregion, the distance is the straight‐line distance between their sampling locations. The horizontal lines show the expectation with the POP and FSP lines slightly jitter of 0.25 so both can be shown.

We summarised the kin classified using the three clustering methods. For the SVM, we used the classifiers trained on the simulated data sets at three migration rates 1%, 0.1%, 0.01% and an all‐scenario SVM from the clutch offspring simulations. The clutch offspring scenario was chosen as an initial inspection of the results indicated many pairs in the FSP range of the kinship‐statistics plots. The total number of kin for the Euclidean clustering and the Manichaikul criteria were 5122 and 5615 respectively. For the SVM classifiers, the number of total kin between classifiers trained at different migration rates was qualitatively similar with 3550 total kin for 0.01%, 4222 at 0.1%, 4286 total kin for 1% migration rate and 3999 total kin for the all‐model SVM. As the qualitative outcomes from all models were similar, we chose the all‐scenario results as they were the most conservative for POPs, returned similar and intermediate (in total number) numbers of FSPs and 2*nd*‐degree relatives, and were trained on the larger data set. In total the all‐model results returned POPs = 22, FSPs = 1205 and 2nd‐degree = 2772, (≈1% of the total number of comparisons) with kin separating reasonably compared to expectation across the kinship statistics (Figure 6). For comparison, the kin classification using the Manichaikul criteria returned similar results with POPs = 26, FSPs = 1861 and 2nd‐degree = 3728. Summarising statistics from the all‐scenario SVM classification, the POPs classified had a mean vector of (*φ*, *k*(0), *k*(2)) = (0.261, 0.021, 0.031) and maximum values of (0.213, 0.036, 0.172). Similarly classified FSP had a mean vector across pairs of (0.223, 0.265, 0.183) and a minimum vector of (0.177, 0.046, −0.104). For 2nd‐degree relatives, the mean vector across pairs was (0.158, 0.427, 0.052) and a minimum vector of (0.111, 0.044, −0.130).

Overall, the dominant proportion of kin pairs (all‐scenario SVM classification) was observed within bioregions with sparse across bioregion kin (Table 2). We observed strong agreement in the pattern of kin across bioregions between the SVM results and the kin determined by the Manichaikul criteria (Table 2 and Table S11). The all‐scenario SVM was more conservative than the Manichaikul criteria with many fewer FSPs and 2nd‐degree relatives reported, which is expected given the lower false‐positive rates observed in the simulation study. The kinship statistics for across‐bioregion kin pairs were well distributed throughout the kinship statistic range suggesting that these pairs are not just false positives at the threshold boundary of kin‐pair types (Figure S27). We expect the qualitative conclusions for across‐bioregion kin pairs to be the same given any of the used kin clustering methods.

**TABLE 2:** Summary of reported PC‐Relate kin‐pairs versus number of pairwise comparisons between and within bioregions.–

	Gulf plains			Cape York		Princess‐Charlotte Bay	Coastal plains		
	ALD	NFD	MGD	North west	North east		CA	APrR	Fitzroy
Gulf Plains – ALD	$\frac{\left(0,1,3\right)}{21}$	$\frac{\left(0,0,0\right)}{728}$	$\frac{\left(0,0,0\right)}{91}$	$\frac{\left(0,0,0\right)}{2394}$	$\frac{\left(0,0,0\right)}{77}$	$\frac{\left(0,0,0\right)}{1456}$	$\frac{\left(0,0,0\right)}{1183}$	$\frac{\left(0,0,0\right)}{539}$	$\frac{\left(0,0,0\right)}{119}$
Gulf Plains – NFD		$\frac{\left(0,137,144\right)}{5356}$	$\frac{\left(0,2,2\right)}{1352}$	$\frac{\left(0,0,1\right)}{35568}$	$\frac{\left(0,0,0\right)}{1144}$	$\frac{\left(0,0,0\right)}{21632}$	$\frac{\left(0,0,0\right)}{17576}$	$\frac{\left(0,0,0\right)}{8008}$	$\frac{\left(0,0,0\right)}{1768}$
Gulf Plains – MGD			$\frac{\left(0,4,6\right)}{78}$	$\frac{\left(0,0,0\right)}{4446}$	$\frac{\left(0,0,0\right)}{143}$	$\frac{\left(0,0,0\right)}{2704}$	$\frac{\left(0,0,0\right)}{2197}$	$\frac{\left(0,0,0\right)}{1001}$	$\frac{\left(0,0,0\right)}{\;221}$
North West Cape York				$\frac{\left(10,176,468\right)}{58311}$	$\frac{\left(0,2,2\right)}{3762}$	$\frac{\left(0,0,0\right)}{71136}$	$\frac{\left(2,1,11\right)}{57798}$	$\frac{\left(0,0,0\right)}{26334}$	$\frac{\left(0,0,0\right)}{5814}$
North East Cape York					$\frac{\left(0,0,0\right)}{55}$	$\frac{\left(0,0,1\right)}{2288}$	$\frac{\left(0,0,0\right)}{1859}$	$\frac{\left(0,0,0\right)}{847}$	$\frac{\left(0,0,0\right)}{187}$
Princess Charlotte Bay						$\frac{\left(1,84,285\right)}{21528}$	$\frac{\left(0,4,16\right)}{35152}$	$\frac{\left(1,26,36\right)}{16016}$	$\frac{\left(0,0,0\right)}{3536}$
Coastal Plains – CA							$\frac{\left(5,104,321\right)}{14196}$	$\frac{\left(1,1,50\right)}{13013}$	$\frac{\left(0,0,0\right)}{2873}$
Coastal Plains – APrR								$\frac{\left(2,638,1357\right)}{2926}$	$\frac{\left(0,0,0\right)}{1309}$
Fitzroy									$\frac{\left(0,25,69\right)}{136}$

*Note*: Each numerator in the cell has a vector of three numbers, which correspond to the number of POPs, FSPs and 2nd‐degree relatives detected within or between bioregions respectively. The denominator corresponds to the number of comparisons performed to detect this number of kin. Grey cells indicate where kin were detected. Individuals (*N* = 2) from the Coastal Plains – CMC bioregion are integrated into the Princess Charlotte Bay bioregion as the results were uninformative.

Bioregion abbreviations: ALD, Albert‐Leichhardt drainage; APrR, Ayr – Proserpine – Rockhampton; CA, Cooktown – Ayr; MGD, Mitchell‐Gilbert drainage; NFD, Norman‐Flinders drainage.

Reported FSPs and 2nd‐degree relative pairs were observed at a maximum coastal distance of 1900 km and were between several individuals from the North West Cape York and Coastal‐Plains – CA bioregions, which correspond to the cross‐bioregion set in Table 2. Parent‐offspring pairs were predominantly observed at 10 km from each other with four long‐range pairs at a coastal distance >400 km (Table S12). All POPs showed length and birth class differences consistent with this kin type. All long‐distance across bioregion kin pairs were investigated as potentially a result of translocation (see below).

Out of all the kin pairs reported, 91% were between individuals sampled less than 50 km from each other (Figure 6). Kin pairs were detected in all bioregions sampled with some across‐ bioregion kin pairs found. Substantial cross‐bioregion kin pairs were reported between the Coastal Plains – APrR and the Coastal Plains – CA, Princess Charlotte Bay (Table 2). Kin pairs were also reported between the North West Cape York and Coastal Plains – CA bioregions; many comparisons (58,638) were performed between these bioregions. Again, most kin pairs reported were found within bioregions, with the Coastal Plains – APrR and Fitzroy bioregions showing the most extreme numbers of kin pairs (≈70%) relative to the number of comparisons performed (Table 2). We visualised the geographic region of the Proserpine River and the spatial distribution of kin pairs to provide insight into the range of kin in this bioregion. The Proserpine region appears densely populated by highly related crocodiles within 10 km of the river's entrance (Figure S28).

### 3.4 Removal of possible translocated individuals

We explored the implications of potentially translocated individuals on genetic variation because translocation was a historical component of the *C. porosus* management program in Queensland between 1995 and 1999 (Brien et al., 2017). We removed individuals that showed unexpected across‐bioregion DAPC results and had high likelihood based on cross‐checked translocation records. Table 3 shows the summary of the DAPC analysis (lowest BIC, *K* = 8) with sets of individuals labelled as possible or almost certainly translocated individuals based on this process.

**TABLE 3:** Summary of possible and likely translocated individuals partitioned by DAPC lowest BIC grouping.

DAPC cluster	2	4	6	5	3	1	8	7	Total T	Total P
Gulf Plains – ALD	7	0	0	0	0	0	0	0	0	0
Gulf Plains – NFD	101	3	0	0	0	0	0	0	0	0
Gulf Plains – MGD	6	7	0	0	0	0	0	0	0	0
North West Cape York	8	263	54	16	0	1 (T)	0	0	1	0
North East Cape York	0	1	0	10	0	0	0	0	0	0
Princess Charlotte Bay	0	1 (P)	0	117	89	0	1 (T)	0	1	1
Coastal Plains – CA	5 (T)	6 (T)	0	18 (P)	3 (P)	136	1 (P)	0	11	22
Coastal Plains ‐ APrR	0	0	0	0	0	0	77	0	0	0
Fitzroy	0	1 (T)	0	0	0	1 (T)	0	15	2	0

*Note*: Table shows a geographically reordered Table S8 with cells that contain the dominant number of individuals coloured grey to highlight trend. Cells (coloured in darker greys) are further marked as possible/probable (P) and quite definite translocations (T) because (a) they encompass adjacent bioregions between which migration is possible (and has clearly occurred across comparable distances in Northern Cape York and the southern Gulf Plains) but (b) they also encompass bioregions in which deliberate translocations are known to have occurred in the 1990s and early 2000s (Queensland Department of Environment and Science internal records).

The DAPC data showed individuals that span many bioregions within clusters. Clusters 2 and 4 are dominated by individuals from the Gulf Plains and North West Cape York, but include a few individuals from Coastal Plains – CA, a single individual from North East Cape York, and individuals from Princess Charlotte Bay and the Fitzroy in cluster 4 (Table 3). Cluster 5 is dominated by individuals from Princess Charlotte Bay and North East Cape York along with a substantial set from North West Cape York and Coastal Plains – CA. Likewise, cluster 1 shows disjunction between North West Cape York and the Coastal Plains – CA region and to the south between Coastal Plains – CA and the Fitzroy. Cluster 8 shows marked disjunction between the Coastal Plains – APrR and Princess Charlotte Bay and Coastal Plains – CA. The kin‐pair data showed similar disjunction between North West Cape York and Coastal Plains – CA despite a high number of pair comparisons in Princess Charlotte Bay. Further possible translocations were investigated using historical records and location information between Princess Charlotte Bay and Coastal Plains – CA bioregions with expected migration from Princess Charlotte Bay to northern areas of Coastal Plains – CA. These seemingly anomalous results from the DAPC and kinship analyses corresponded very closely with known sites of translocations. The absence of geographically intermediate results (DAPC and kinship) along the coast was used as further evidence for translocation rather than migration.

In total there were 38 possibly translocated individuals (Table 3). Rates of translocations (percentage of total 1,176 samples taken) were calculated for each bioregion containing likely translocations and for the pre‐2000 and post‐2000 period when translocations stopped being used in the management program (Table S13). Rates of translocation were highest in the post‐2000 Fitzroy and likely attributable to farm escapees. Rates were also higher in the Coastal Plains – CA bioregion and in particular the pre‐2000 period. Further analysis showed that a nest of 8 juveniles was removed as part of a pre‐2000 translocation set in the Coastal Plains – CA. Rates were more consistent with post‐2000 values when only one individual from this nest set was used (Table S13).

To assess the impact that these individuals could have on the results, we recomputed the genetic diversity statistics, fixation indices and the performed again the correlation analysis between *F*_ST_ and coastal distance with these individuals removed. We further retabulated the kin observations with these individuals removed. These analyses were expected to be the most influenced by the removal of these individuals.

Overall, the diversity statistics showed similar results to those from the analysis with the full set of individuals (Table S14). Small changes were observed for the Coastal Plain – CA bioregion, which is expected given 33 individuals were removed from this bioregion. The greatest changes were observed for the Fitzroy bioregion with a substantial increase in monomorphic loci, a drop in allelic richness from 1.41 to 1.32, and *F*_IS_ decreasing from −0.001 to −0.141. Similarly, for the *F*_ST_ analyses the greatest changes were for the Fitzroy with an increase in *F*_ST_ for all bioregion comparisons (Figure S29). The relationship was similar for the correlation of *F*_ST_ with coastal distance and the Mantel test correlation close to that reported previously at approximately 0.8. The Coastal Plains – APrR / Fitzroy comparison remained an outlier (Figure S29).

The kinship summaries showed substantially reduced across‐bioregion kin pairs (Table S15 and Figure S30). Across‐bioregion kin pairs were no longer observed between the Coastal Plains – CA and the North West Cape York and Princess Charlotte Bay bioregions. Similarly, for the Coastal Plains – APrR bioregion shared kin were no longer observed in the Coastal Plains – CA and Princess Charlotte Bay bioregions. The greatest coastal distance between a reported first and second order kin pair was reduced from 1900 to 547 km. This long‐distance kin pair consisted of individuals located in Bloomfield and Townsville within the Coastal Plains – CA bioregion.

## 4 DISCUSSION

This study has performed a comprehensive investigation of the genetic variation of Queensland's saltwater crocodile population. Importantly, the study contains samples from nearly the entire 4500 km coastal range of the species and gives insights into the similarities and differences across climatically and physiographically diverse regions.

### 4.1 Substantial structure

The study shows unequivocally that it would be misleading to characterise saltwater crocodiles in Queensland as a single population or a single management unit for conservation purposes. There is clear evidence of substantial population structure across the species' range, with each bioregion showing some differentiation from neighbouring bioregions and the degree of differentiation, based on fixation and genetic diversity indices, increasing with geographical distance. The PCA scatterplot visually implied five distinct populations and the STRUCTURE and DAPC analyses showed corroborating evidence that five populations partition well the genetic variance in the data set. As the number of assumed population clusters (*K*) increased in the STRUCTURE analysis there was an initial partitioning of the eastern and western populations. As *K* increased further, north–south differentiation emerged within these east–west groups. The five populations include the Gulf Plains, North West Cape York (includes Torres Strait), North East Cape York and Princess Charlotte Bay, Coastal Plains – CA and Fitzroy, and the Coastal Plains – APrR.

The *F*_ST_, DAPC and kinship analyses show evidence of additional sub‐structure, which has not been fully resolved in this dataset. For example, comparisons between bioregions within the five broad populations showed *F*_ST_ values greater than zero. DAPC clustering showed a BIC‐minimum eight‐population configuration providing additional evidence for substructure within these broad populations.

### 4.2 Interpretation is more difficult because animals have been translocated

The PCA, DAPC, and STRUCTURE analyses showed evidence of a small number of migrant or translocated individuals (or their close descendants) that were sampled in the Coastal Plains – CA but genetically clustered to the Gulf Plains – NFD, North West Cape York, Princess Charlotte Bay, and Coastal Plains – APrR bioregions. Furthermore, a smaller number of crocodiles sampled in North West Cape York, Princess Charlotte Bay, and Fitzroy were also genetically clustered in other bioregions. The most plausible explanation is that these individuals have arisen from deliberate translocations as part of the Queensland Government's management program (Brien et al., 2017). Evidence in support of this conclusion comes from several observations.

The Queensland Government's management program has involved transporting problem crocodiles from some remote areas to farms and zoos on the east coast between Cairns and Rockhampton (Brien et al., 2017). Known transfers have included animals from the southern Gulf Plains, Weipa, and Princess Charlotte Bay. Escape of wild‐captured crocodiles from farms and zoos is known to have been quite common, and most of the individuals found on the Coastal Plains that appear to derive from remote areas were found in waterways not far distant from crocodile farms and zoos. Also, between 1995 and 1999 some problem crocodiles were relocated from the east coast south of Cooktown to remote areas, including Princess Charlotte Bay and North West Cape York (Brien et al., 2017).

The DAPC data and kinship analyses provide additional support for translocation over natural migration as the likeliest explanation. It was therefore prudent to investigate the results with these animals treated as the product of recent translocations rather than historical migration. In total there were 38 possible and likely translocated individuals determined. Eight of these individuals were hatchling siblings (validated by kinship analysis) captured in November 1999 and are taken to be progeny from a single animal translocated earlier. Thus, the actual number of translocations identified can be reduced to 31 (approximately 3% of the whole dataset). The total translocation sampling rate may be inflated due to tissue sampling being more likely for translocated individuals as samples may be taken during translocation.

Removal of the 38 possible and likely translocated individuals showed similar results for diversity statistics and *F*_ST_ to the larger analysis. The exception was the Fitzroy which showed evidence for lower allelic richness and inbreeding. This was expected given the two very likely translocated individuals removed from the Fitzroy were from the Gulf Plains and North West Cape York, which showed substantial genetic divergence from the Fitzroy. When translocated individuals were removed, the DAPC clustering further separates three remote clusters and the Coastal Plains – CA cluster into sub‐clusters which show either no linkages to other bioregions or distinct evidence of genetic exchange. Importantly, it also separates the southern Coastal Plains cluster into distinct Fitzroy and Coastal Plains – APrR sub‐clusters. The results of between‐ bioregion close kin also reduced dramatically with the longest distance kin pair observed at a coastal distance of 547 km in a single bioregion – down from approximately 1900 km in the expanded analysis. The biggest set of possible translocations involved 14 individuals (after accounting for the 8 hatchling siblings) from the Coastal Plains – CA region clustered (DAPC) with individuals from Princess Charlotte Bay under DAPC. Natural migration from Princess Charlotte Bay is quite possible and animals migrating south would encounter sequentially small centres of human population at Cooktown, Bloomfield, Daintree and Port Douglas where the likelihood of being reported as problem animals and trapped would be highest. Seven of the 14 possible translocations were sampled at Cooktown, Bloomfield or Port Douglas, well away from crocodile farms and zoos. The other seven were from areas further south and are more likely to be farm/zoo escapees or their progeny. Thus, the data are ambiguous and do not allow firm conclusions about whether migration into the Coastal Plains – CA bioregion is a significant issue.

Increasingly translocations are used as a tool for conserving biodiversity, particularly for managing threatened and keystone species, to maintain biodiversity and ecosystem function under the combined pressures of habitat fragmentation and climate change (Weeks et al., 2011). Assessment of conservation relocations is rarely carried out with outcomes often unknown, and causes of failures rarely understood (Fischer & Lindenmayer, 2000). In our study, the translocation assessment was coincident with the broader analyses and highlighted that translocation can mask migration and loss of genetic diversity (e.g., Fitzroy population). For future management, focussed translocation could be a viable conservation action for restoring genetic variability in the southern Queensland populations, with ongoing genetic monitoring required to assess its impact.

### 4.3 Limited dispersal and site fidelity – comparisons with Northern Territory, Australia

After excluding translocations, the DAPC clustering results suggest that genetic exchanges have historically been quite constrained geographically to within one or two adjacent bioregions. More distant exchanges are better explained by management translocations than any natural movement. This finding is supported by the kinship analysis which shows that most kin pairs (all‐scenario SVM classification) were observed within bioregions and 91% of them were <50 km apart. This suggests very limited long‐distance dispersal and a tendency for reproductive philopatry. This contrasts greatly with findings of Fukuda et al. (2022) who reported that *C. porosus* populations in the Northern Territory are linked by extensive dispersal, with 42% of sampled crocodiles dispersing from their estimated natal area with a most common dispersal distance of 150–200 km and some dispersing up to 600–700 km. This is a striking difference that suggests some important contrasts between the two study populations that could be explained by very consistent dispersal drivers and processes with altered constraints on factors such as climate, habitat, and population density.

There is evidence of substantial population substructure across the species' Queensland range, with each bioregion showing some differentiation from neighbouring bioregions and strong differentiation over large geographical distance based on fixation and genetic diversity indices. Recent population genetics analysis of *C. porosus* in the Northern Territory, showed very comparable differentiation between populations across similar geographical distances to the Queensland population (Fukuda et al., 2019). Fixation indices for freshwater crocodiles (*Crocodylus johnstoni*) from the Kimberley, Western Australia (Cao et al., 2020) showed greater differentiation over smaller geographic distances than observed for *C. porosus* in our study, as might be expected from the very limited capacity of *C. johnstoni* to exploit coastal waters to move between catchments and their tendency to greater site fidelity (Tucker et al., 1997). *C. porosus*, in contrast, has well‐documented long‐distance movement capabilities including in coastal waters (Campbell et al., 2013; Fukuda et al., 2019; Read et al., 2007).

In the Northern Territory, Fukuda et al. (2022) concluded that dispersal of *C. porosus* was greatest from high density populations at or near capacity with access to extensive high‐quality breeding habitat. In Queensland, the bioregion most comparable with the prime habitat in the Northern Territory is North West Cape York, which contains 40% of the total *C. porosus* population in Queensland and the largest area of high‐quality nesting habitat (Taplin, 1987; Taplin et al., 2020). Nests in Port Musgrave produce some hundreds of hatchlings annually, yet population counts have been essentially stable numerically since the late 1980s (Taplin et al., 2020). We might expect this bioregion to be an important source of dispersing juveniles and subadults populating adjacent bioregions and low‐density river systems further afield in the southern Gulf. DAPC data suggests that has historically been the case to a modest extent, but kinship data shows little evidence of it in contemporary timeframes, with just one (2nd‐order) kin‐pair identified in some 42,000 kin‐pair comparisons within the Gulf Plains.

Future work to compare landscape genetics results from the Queensland population with those reported in Fukuda et al. (2022) could further help to disentangle how the environmental differences between the two States influence connectivity and dispersal. This would require the characterisation the Queensland landscape features relevant to crocodile dispersal, which will be complex. Queensland has large regions similar in physiography to the Northern Territory, with extensive plains subject to periodic flooding and seasonally diffuse boundaries between catchments but also large areas with very narrow coastal plains, steeper topography, and river systems well‐separated by coastal hills and ranges. Queensland's crocodiles also inhabit a very wide range of climates which, at their southern extremities, are likely to influence the timing and extent of seasonal movements including through energetic constraints at low temperature. There are also some potentially important barriers to movement and dispersal linked to large tidal movements and prevailing currents that may be influential in isolating the Coastal Plains – APrR and Fitzroy subpopulations from each other and from more northerly subpopulations (Taplin et al., 2021, Appendix 10).

### 4.4 Limited dispersal and site fidelity – environmental and density dependent factors

The environmental factors leading to the marked differences between the Northern Territory are obscure. There are no obvious physical barriers to coastal dispersal of crocodiles from North West Cape York to the southern Gulf and one large individual has been satellite‐tracked making excursions from Port Musgrave to the Norman River and back (Campbell et al., 2010). Read et al. (2007) and Campbell et al. (2010) also reported adult male crocodiles moving around the cape between North East Cape York and North West Cape York. While the Torres Strait, with its complex and turbulent tidal regime, might be expected to prove a barrier to dispersal from North West Cape York to the east, DAPC results suggests there has been exchange between the high‐density populations of North West Cape York and the medium‐density populations of Princess Charlotte Bay, while kin‐pair data show modest exchanges with North East Cape York but none with Princess Charlotte Bay.

Some part of the difference between Northern Territory and Queensland results may best be explained by the different trajectories of the two populations after they were protected in the early 1970s. The Northern Territory population was protected to some extent by very extensive freshwater swamplands that were difficult to hunt and which consequently protected a significant remnant population of breeding‐size adults while the broader population was driven to commercial extinction (Webb et al., 1984). The Queensland population, in contrast, is largely confined to riverine habitats where crocodiles were more vulnerable to hunting pressures and often driven to extremely low densities. Despite 11 years of protection, spotlight surveys of 424 km of five southern Gulf rivers (the Albert, Leichhardt, Bynoe and Smithburne Rivers and Duck Creek) in 1985 revealed counts of only 48 non‐hatchlings (NH) – an average of just one animal per 9 km of waterway (Taplin, 1988). The Norman River had a slightly higher density of 0.33 NH/km (61 NH across 183 km). Counts in 1984–86 of 261 km of coastal streams in the Coastal Plain – CA bioregion found only 52 non‐hatchlings. Spotlight densities in the five Gulf rivers were quite uniformly low (range 0.09–0.19 NH/km, median 0.12 NH/km) but were more variable and highly skewed on the east coast (range 0–2.67 NH/km, median 0.05 NH/km or 1 NH per 20 km). No crocodiles were sighted in 9 of the 20 Coastal Plains – CA waterways surveyed in the 1980s (Taplin, 1988).

Since 1971 the Northern Territory population has recovered rapidly from hunting and has grown to high average density (>5.26 non‐hatchlings per km) and high absolute numbers (100,000 or more) (Fukuda et al., 2020). Queensland's population, on the other hand, has shown overall a much slower rate of increase and has reached only 1.65 non‐hatchlings per km compared with 5.26 across surveyed rivers in the Northern Territory (Taplin et al., 2020). Given abundant evidence of density‐dependent processes in *C. porosus* populations (summarised in Fukuda et al. (2020)), it is plausible the Queensland population has not reached sufficiently high densities across a broad enough area to trigger the levels of long‐range dispersal that would be detectable in the kinship analyses. If that is the case, then it will be important to consider both the long‐ and short‐term components of genetic relationships in management plans. Continuing pressure on local populations through management interventions may prevent them ever reaching levels where ‘natural’ exchanges with adjoining populations occur and they will need to be considered as essentially isolated/localised populations rather than dynamic components of a broader Queensland‐wide population.

### 4.5 Parent‐offspring pairs

Individuals in parent‐offspring pairs were predominantly found within 10 km of each other (six exceptions likely arising from translocations). However, only a very small proportion of the total kin was POPs (≈0.3%). A contributing factor to few POPs being found is that only 10% of the sampled population was over 2.4 m in TL (approximate initial breeding size for females). Smaller individuals can contribute to POPs but are required to have a birth cohort gap between individuals in the pair greater than the age at sexual maturity, which did occur in our study. Despite the proportion of larger C. porosus capable of breeding (>2 m) increasing over time in Queensland (17% in 1984–1989 to 27% in 2016–2019), population is still dominated by immature animals (Read et al., 2004; Taplin et al., 2020).

### 4.6 Full sibling pairs – mate fidelity

We observed a substantial number of full‐sibling pairs, which could arise for a myriad of potentially interacting reasons including sampling, mate‐fidelity, and large clutch sizes with limited juvenile dispersal among others. A large proportion of FSPs could arise if sampling effort was focused where close relatives aggregate, for example, the sampling of eggs from nests – this was not the case in this study. There are anecdotal reports supporting mate fidelity in places such as the Daintree, Queensland, where mating is primarily reported between the dominant male and several females, with intruding males regularly rejected by the females (D. White 20 years of personal observation). However, Lewis et al. (2013) reported strong evidence of multiple paternity within clutches from wild (Northern Territory: 69%) and captive (Queensland: 38%) *C. porosus* clutches and concluded that a promiscuous mating system characterised the species. If mate fidelity was the main driver of the FSP observations, then we would expect pairs to be of different age classes. Reliable age information would be required to resolve this, or approximate inference made with size or distances between kin. Further data and investigation are required to disentangle the relative contribution of sampling design, large clutch sizes, mating system, and limited juvenile dispersal to the rate of full siblings observed in the population.

### 4.7 Proserpine and Fitzroy – small, isolated, and low genetic diversity

The Proserpine and Fitzroy Rivers were each found to be populated by highly related individuals, with the most extreme numbers of kin relative to the comparisons performed (68% and 69% respectively). One of the highest counts of FSPs was found in the Proserpine River, which has a very high density of very large adult crocodiles (1.3 per km) in just 22 km of waterway (Taplin et al., 2020; Taplin et al., 2021). Furthermore, despite their geographical proximity these systems showed no evidence of being connected across any of the analyses.

The historical, environmental, and biological reasons for the low genetic diversity and seeming isolation of these populations are not clear, but some possibly relevant factors can be identified. The Proserpine River was historically bounded by extensive swamplands on the Goorganga Plains which would have provided much favourable habitat and nesting sites but has largely been replaced by intensive pastoral development (Taplin et al., 2021). Crocodiles appear to have been largely confined to just 20–30 km of inhabitable streams and nesting is confined to marginal sites under fringing forest and woodland. The high count of FSPs here may be attributable to the scarcity of good nesting sites and successful recruitment deriving from just a handful of parents controlling favourable sites.

The apparent isolation of both the Proserpine and Fitzroy River sub‐populations may be attributable, in part, to peculiarities of the environment in the southern extremities of the crocodile's range. The Fitzroy River is the most southerly known breeding location for saltwater crocodiles in Australia. It is a large, deep‐water system separated from the much smaller Proserpine system by a region of very high tidal range and fast tidal currents centred on the large shallow embayment of Shoalwater Bay and Broad Sound. Both systems are flanked largely by small, very shallow waterways interspersed with long stretches of coastline that make for poor crocodile habitat. Low‐tide helicopter survey in 2021 and analysis of the tidal regime showed that many of these systems, extending over 1300 km of coastline and including the major embayments, reduce on low tides to mud and sandbanks and shallow gutters, making them highly unfavourable for crocodiles (Taplin et al., 2021). Only 35 crocodiles were sighted in 490 km of streams across 32 coastal systems outside the Proserpine and Fitzroy Rivers, for an average density of only 0.07 non‐hatchlings per km (≈1/70 of the density in the Proserpine River). Twenty‐three systems had no crocodiles and no tracks or traces visible. It seems highly likely that this adverse climatic and physiographic environment is contributing to the genetic isolation observed.

The evidence for low genetic diversity in the Fitzroy should be interpreted cautiously because the number of individuals sampled was small and the results could be a sampling artifact. In particular, a potential contributing factor to the spatial patterns of diversity could be an ascertainment bias associated with the isolation‐by‐distance setting, where SNPs with alleles that are common in the populations on the edge of the sampling, or in populations not present in the loci selection phase, are more likely to be filtered out. This bias may in part be mitigated by the DArTCap data containing approximately 50% new loci not requested from the discovery phase. There is some genetic evidence linking the Fitzroy historically with the Coastal Plains Cooktown‐Ayr bioregion but, given very large numbers of crocodiles have been transferred since the early 1980s to a crocodile farm near Rockhampton, the contribution of translocation versus natural migration to genetic variation is difficult to estimate. The results of this study support the idea that along with sub‐optimal ambient temperatures, localised tidal conditions and marginal habitat may also inhibit dispersal and expansion of *C. porosus* down the south‐eastern coast of Queensland (Read et al., 2004; Taplin, 1987; Taplin et al., 2020; Taplin et al., 2021).

### 4.8 Close kin and kinship analyses

Our results demonstrate that saltwater crocodiles in Queensland show limited reproductive dispersal with strong reproductive philopatry in both sexes. This generates highly differentiated populations within bioregions and local estuarine systems. Limited reproductive dispersal has implications for kinship analyses because it increases the potential for individuals to mate with close relatives. Kinship analyses rely on clear discrimination of kinship statistic classes. We observed no clear separation of classes for the saltwater crocodile population, which may be due to (but not limited just to) the number of SNPs available for kinship statistic calculations. The overlap of classes may also be attributed to inbreeding creating intermediate kinship classes that span the canonical categories (e.g., POPs, HSPs). The second hypothesis has implications for future analyses, where even if we could increase the number of SNPs to the scale of Conomos et al. (2016) then the separation of POPs, FSPs and HSPs from each other and less‐related pairs would still be challenging.

Reconstructing the historical demography of a species and making inferences about genetic population structure and kinship are essential to establishing better conservation priorities and management policies for wild species of high importance. This analysis provides a baseline for potential population monitoring of abundance using close‐kin mark‐recapture (CKMR) (Bravington et al., 2017; Bravington, Skaug, & Anderson, 2016; Hillary et al., 2018) but would require reliable age and sex information, advances in spatially varying sample probabilities as in Conn et al. (2020), and extensions of CKMR theory to accommodate large clutches and limited dispersal. At a minimum, accurate juvenile age information is required with multiple juvenile cohorts sampled spanning multiple birthing seasons. Given appropriate sampling, potential between‐bioregion movements could also be inferred for different birth cohorts. Cross‐cohort HSPs provide sex‐specific information about their parents' movements between breeding events. They have the potential to reveal very recent barriers to gene flow and quantify contemporary migration rates between populations (Feutry et al., 2017, 2020). Furthermore, recent research has used the spatial distribution of close‐kin to assess whether a population is currently totally philopatric, panmictic or exhibits sex‐linked reproductive connectivity and dispersal (Patterson et al., 2022). Potentially, epigenetic age from the biopsy used for this study could be performed but would require an initial validation data set with known age (Husby, 2022; Mayne et al., 2021). Future analysis of mitochondrial DNA combined with epigenetic and subsequent kinship analyses has the potential to reveal sex‐specific structure at a finer scale. This would be a substantial advance because aging techniques for crocodiles have proved very problematic due to the remobilisation of calcium in bones that obscures inference from growth ring aging methods.

### 4.9 Future sampling

Genetic sampling of the Burdekin River catchment would likely give additional insights into the effects of agricultural development on the Queensland population. This river extends over 200 km inland and a small breeding population of saltwater crocodiles has persisted at low density for some decades – concentrated between the Burdekin Dam Wall at 167 km inland and Clare Weir at 58 km (Taplin et al., 2021; Taplin & Pople, 1987). The population appears to have become largely isolated from the coast by a series of weirs and dams constructed between the 1950s and the late 1980s. It likely persisted in this inhospitable habitat despite hunting because the river is very wide, strewn with extensive sand and rock bars and is barely navigable in even a small boat over long reaches. A modest population increase in recent decades (Taplin et al., 2021) has likely been driven by very occasional successful nesting in a small population of mature animals. Genetic testing seems likely to reveal a highly inbred population with large proportions of close‐kin pairs, similar to the Fitzroy River. Insights into the genetic history of this population would add to our understanding of long‐ and short‐term influences on these rather isolated aggregations. The population is of particular biological and evolutionary interest as it offers opportunities to study salt gland function in individuals that have never been exposed to salt water.

### 4.10 Management implications

The aim of the Queensland saltwater crocodile management program is to balance the need to minimise human–crocodile conflict while not negatively impacting the conservation of the species in the state (Brien et al., 2017). The current management program attempts to reduce the number of crocodiles in and around areas where there are large human populations, while at the same time preserving crocodile numbers in more sparsely populated remote areas and protected habitat along the coastline south of Cooktown (Taplin et al., 2020).

Public discourse on crocodiles and crocodile management in Queensland tends to characterise the population as a single entity and to view its post‐protection recovery and growth as more or less uniform across the State and having much in common with the somewhat spectacular recovery seen in the Northern Territory. That view has been challenged by the extensive analysis of survey results across 40 years in (Taplin et al., 2020), which showed great differences in population density, growth trajectories and rate of population increase across Queensland's diverse geography. This study adds unprecedented detail to that state‐wide picture by highlighting the limited interconnectedness of major regional concentrations of crocodiles, many of which are separated by large expanses of marginal or poor‐quality habitat.

Queensland's saltwater crocodile population is best considered, for wildlife management purposes, to be comprised of six sub‐populations centred in the Gulf Plains, North West Cape York, Princess Charlotte Bay, the eastern Coastal Plains between Cooktown and Ayr, the Proserpine River, and the Fitzroy River. This regionalisation recognises the five sub‐populations that dominated the genetic analysis but separates out also the geographically disjunct Fitzroy River population. Each of these sub‐populations has some connectedness with adjacent populations, but that connectedness is much greater between the Gulf Plains and North West Cape York bioregion than it is between the eastern coastal plains sub‐populations. Indeed, the Proserpine River presents as surprisingly disjunct from the Coastal Plains – CA and Fitzroy River sub‐populations – perhaps resulting from inhospitable habitat and unfavourable tidal regimes peculiar to that part of Queensland (Taplin et al., 2020; Taplin et al., 2021). These are important findings that need to be incorporated into future management regimes, not least because they suggest that the effects of management actions will be far more localised than we have thought previously and there will be less potential for adverse effects on local populations to be ameliorated by dispersal from distant sources of recruitment.

This is brought into particular focus by the ambiguous findings of this study in relation to Princess Charlotte Bay and its important sub‐population centred on Rinyirru‐Lakefield National Park. The crocodile population there has increased considerably since the first formal surveys of the late 1980s (Taplin et al., 2020) and is a potentially important source of dispersing juveniles and sub‐adults available to populate adjacent bioregions. The DAPC cluster analysis summarised in Table 3 shows some interchange with North West Cape York and, to the south, with the Coastal Plains – CA bioregion. But we know that for some years in the late 1990s considerable numbers of crocodiles were moved between these three bioregions as part of Queensland's problem crocodile management effort. Princess Charlotte Bay is separated from the main centres of crocodile population in the North West Cape York (at Albatross Bay and Port Musgrave) by some 900 km of mostly low‐quality habitat (Taplin & Pople, 1987) and the complex tidal regimes of the Torres Strait. Natural dispersal cannot be ruled out but, if present, is confounded with known translocations. The same is true of exchanges with the Coastal Plains – CA bioregion to the south, which is similarly separated from Princess Charlotte Bay by some 350 km of largely low‐quality habitat. While we have concluded that some of the links uncovered here between Coastal Plains – CA and Princess Charlotte Bay most likely derive from translocated individuals, the evidence cannot exclude the possibility of natural interchange. Given the importance of this issue to practical management actions in the Cairns‐Cooktown region there would be benefit in sampling DNA more intensively across this interchange region if it would help identify and quantify natural movements. The results of this study provide important insights into *C. porosus* dispersal and connectivity that can directly inform future management of the species in the state, especially along the northern east coast where human‐crocodile conflict is greatest.

## Supporting information


Appendix S1

## Data Availability

Results of the present study were generated using R version 4.1.2. Software to perform the STRUCTURE analyses were downloaded from https://web.stanford.edu/group/pritchardlab/structure_software/release_versions/v2.3.4. Raw and processed genotype data were permanently deposited at CSIRO's Data Access Portal at 10.25919/993z‐me55.
