# Corrigendum

**DOI:** 10.1111/eva.13562

**Published:** 2023-06-22

In Evolutionary Applications 16:4, for the article by Lloyd‐Jones et al. (2023) entitled “Implications of past and present genetic connectivity for management of the saltwater crocodile (*Crocodylus porosus*)” (pages 911–935), the authors would like to update the Acknowledgments section as follows:
