# Supplementary material for: Implications of past and present genetic connectivity for management of the saltwater crocodile (Crocodylus porosus)
Source: Evol Appl. 2023 Mar 31;16(4):911–35. doi: 10.1111/eva.13545 (PMC10130557; doi:10.1111/eva.13545)
Supplement: Supplementary file 1 — Appendix S1 [file EVA-16-911-s001.docx]

# Implications of past and present genetic connectivity for management of the saltwater crocodile (*Crocodylus porosus*) - Supplementary Materials

# Supplementary Figures


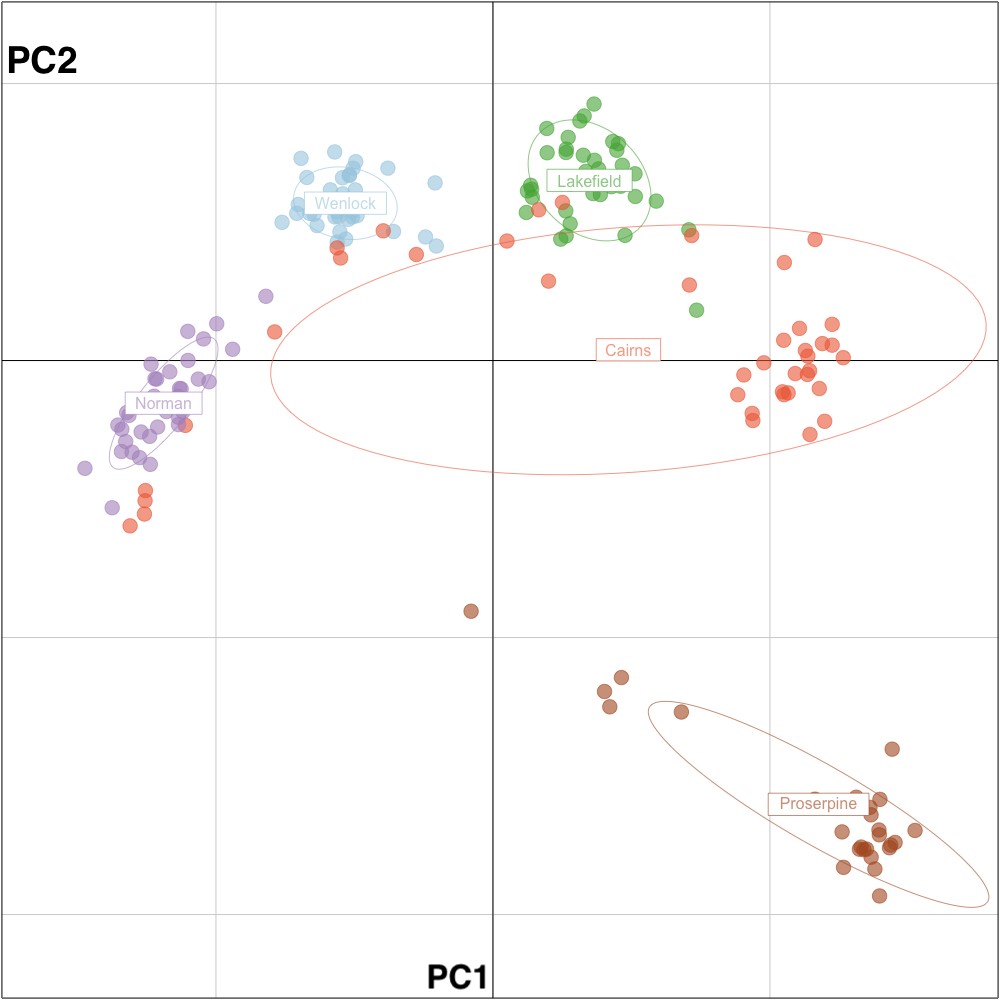


**Supplementary Figure 1 Scatterplot of first two principal components across individuals from the pilot saltwater crocodile genotype data**. The principal components analysis was performed using the post quality control (n = 177 and *p*_markers_ = 4, 799) data and with no filter on Hardy-Weinberg equilibrium. Points are coloured by the sampling location of the individual and labelled with the region in which they were sampled. The first two principal components are plotted with the ellipses drawn to contain most of the points in the region class.


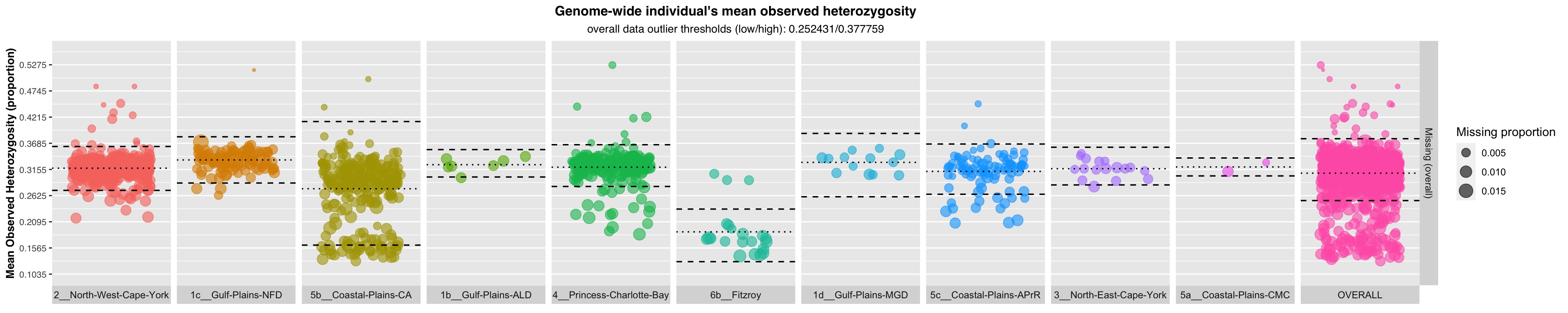
**Supplementary Figure 2 Summary of individual mean heterozygosity partitioned by bioregion**. Figure is produced for quality control inspection by the filter_rad quality control pipeline in the Radiator R package. Panel labels on the x-axis correspond to saltwater crocodile bioregions sampled and each point is the computed heterozygosity for an individual. Horizontal dashed lines represent within region outlier bars for heterozygosity. Bioregion abbreviations are Norman-Flinders drainage (NFD), Albert-Leichhardt drainage (ALD), Mitchell-Gilbert drainage (MGD), Cape Melville – Cooktown (CMC), Cooktown – Ayr (CA), Ayr – Proserpine – Rockhampton (APrR).

4


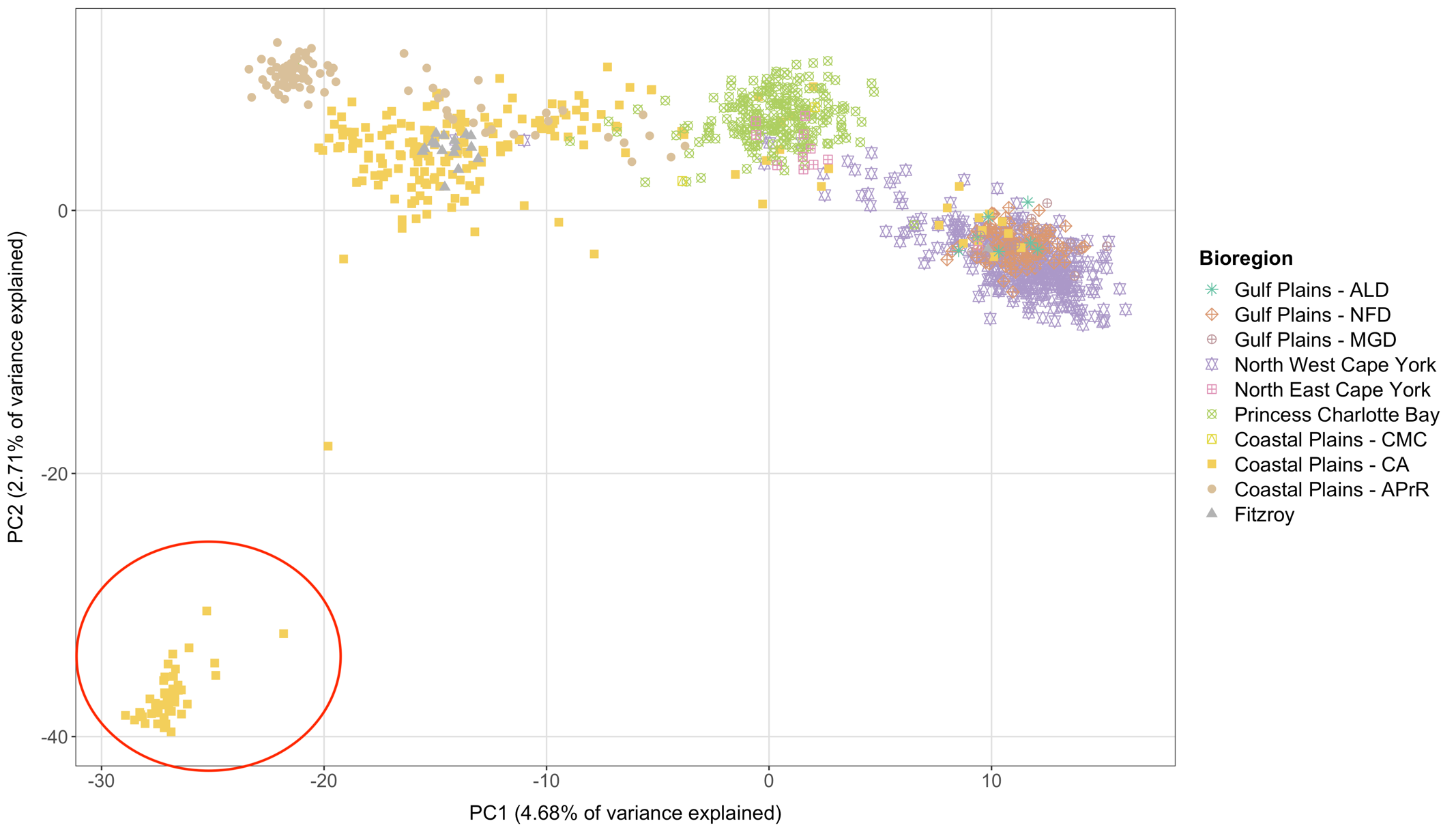


**Supplementary Figure 3 Principal component analysis scatter plot of PC 1 versus PC 2 including low-heterozygosity outliers (red circle - bottom left)**. Bioregion abbreviations are Albert-Leichhardt drainage (ALD), Norman-Flinders drainage (NFD), Mitchell-Gilbert drainage (MGD), Cape Melville – Cooktown (CMC), Cooktown – Ayr (CA), Ayr – Proserpine – Rockhampton (APrR).


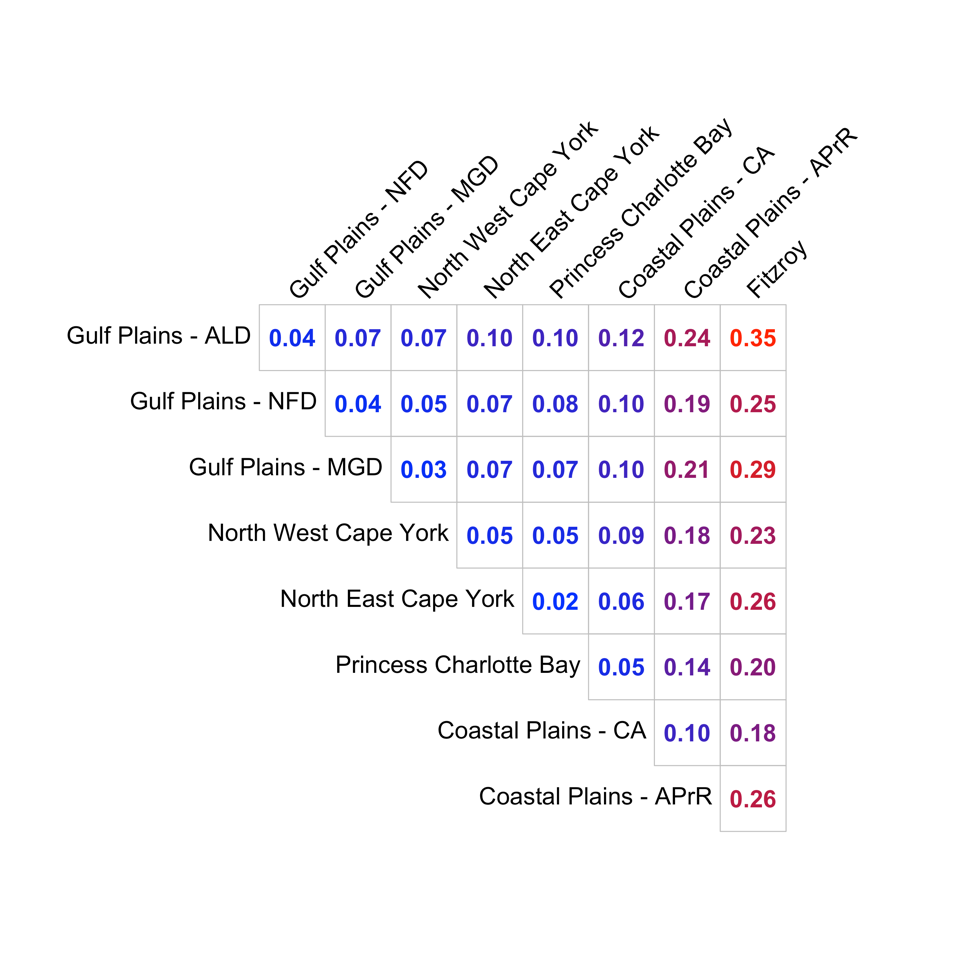


**Supplementary Figure 4 Pairwise FST values between all bioregions calculated from 959 crocodiles and 4,312 SNPs filtered using the Radiator pipeline with no MAC or HWE quality control filtering.** All entries in are significant with bootstrap (replicates = 1,000) p-values less than the FDR adjusted threshold of 0.05. Row and columns are ordered by position along the Queensland coast from west to east. Bioregion abbreviations are Norman-Flinders drainage (NFD), Albert-Leichhardt drainage (ALD), Mitchell-Gilbert drainage (MGD), Cape Melville – Cooktown (CMC), Cooktown – Ayr (CA), Ayr – Proserpine – Rockhampton (APrR).


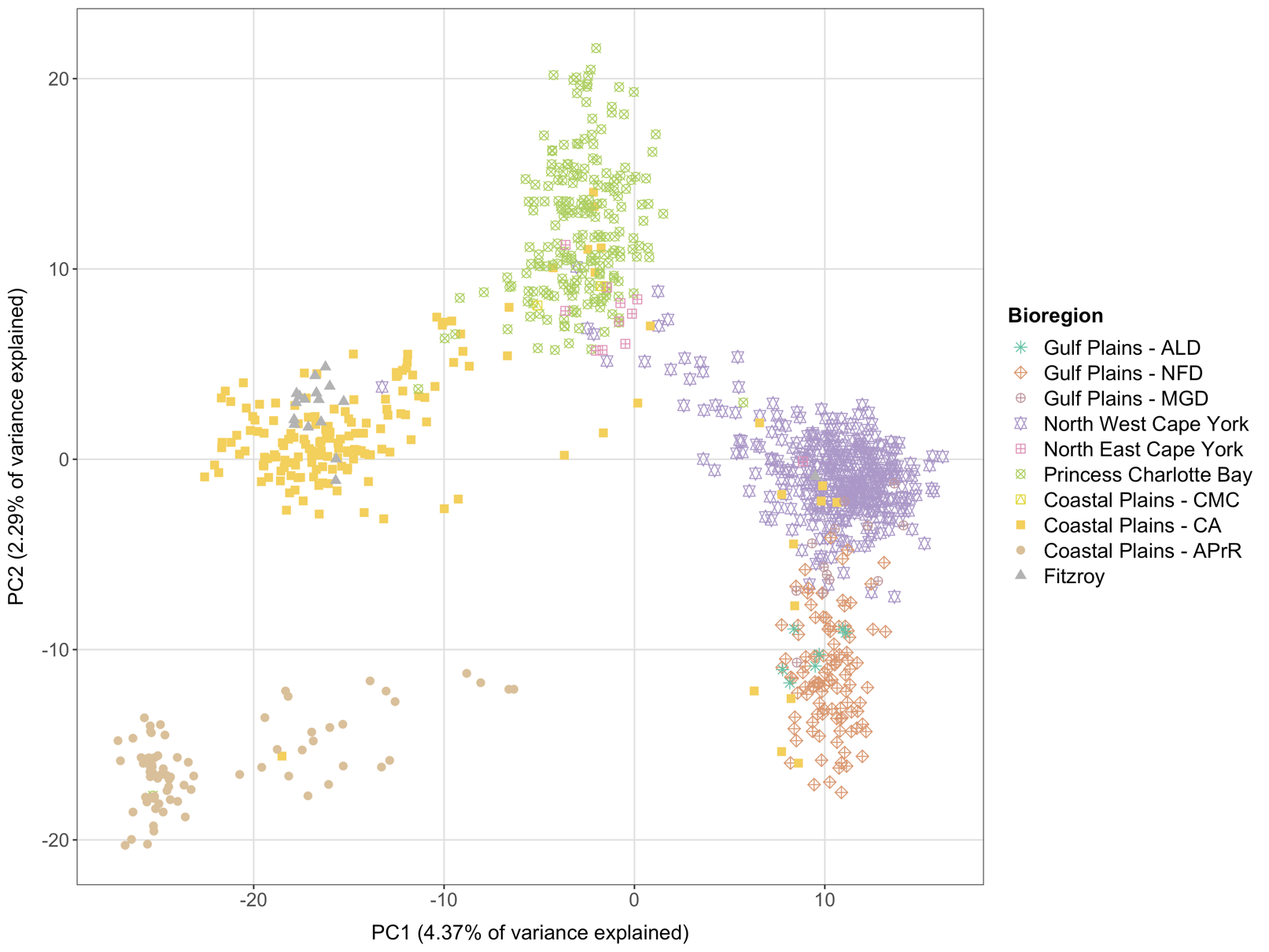


**Supplementary Figure 5 Principal component analysis (PCA) analysis summary plot.** PC 1 (4.4% of the variance) largely differentiates the individuals in eastern populations from those in the western populations. PC 2 (2.3% of the variance) differentiates mostly between the south to the north although North West Cape York are sampled at a higher latitude than the Princess Charlotte Bay samples. Migrant or translocated individuals are apparent, especially in the Coastal Plains - CA region. Likely admixed individuals are also present among the major populations. Bioregion abbreviations are Norman-Flinders drainage (NFD), Albert-Leichhardt drainage (ALD), Mitchell Gilbert drainage (MGD), Cape Melville – Cooktown (CMC), Cooktown – Ayr (CA), Ayr – Proserpine – Rockhampton (APrR).


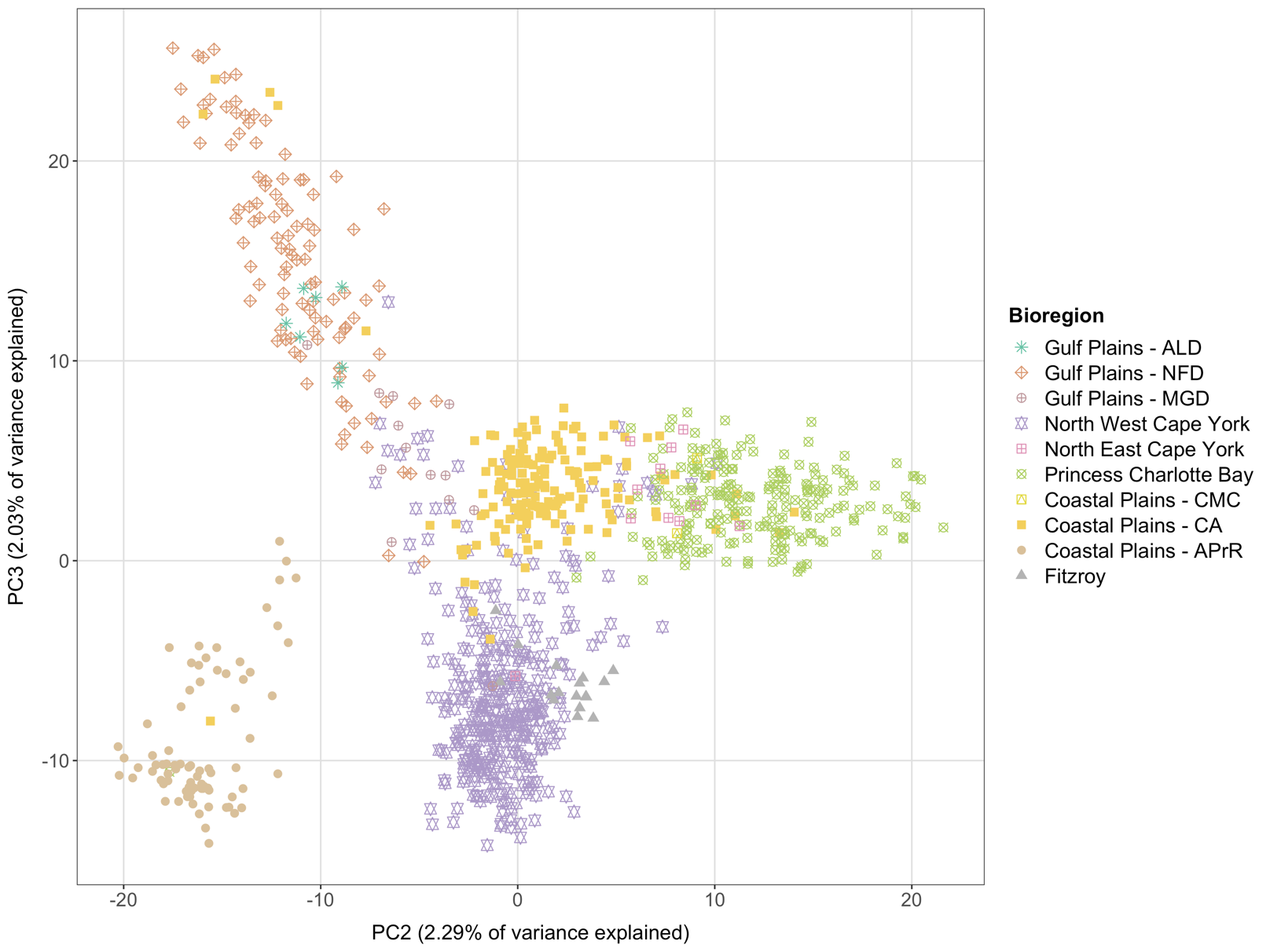


**Supplementary Figure 6 Principal component analysis (PCA) analysis summary plot for PC 2 versus PC 3.** PC 3 (2.03% of the variance) largely differentiates the North West Cape York from the Gulf Plains. The Coastal Plains - APrR is also separated from the Gulf Plains along PC3. Bioregion abbreviations are Norman-Flinders drainage (NFD), Albert-Leichhardt drainage (ALD), Mitchell Gilbert drainage (MGD), Cape Melville – Cooktown (CMC), Cooktown – Ayr (CA), Ayr – Proserpine – Rockhampton (APrR).


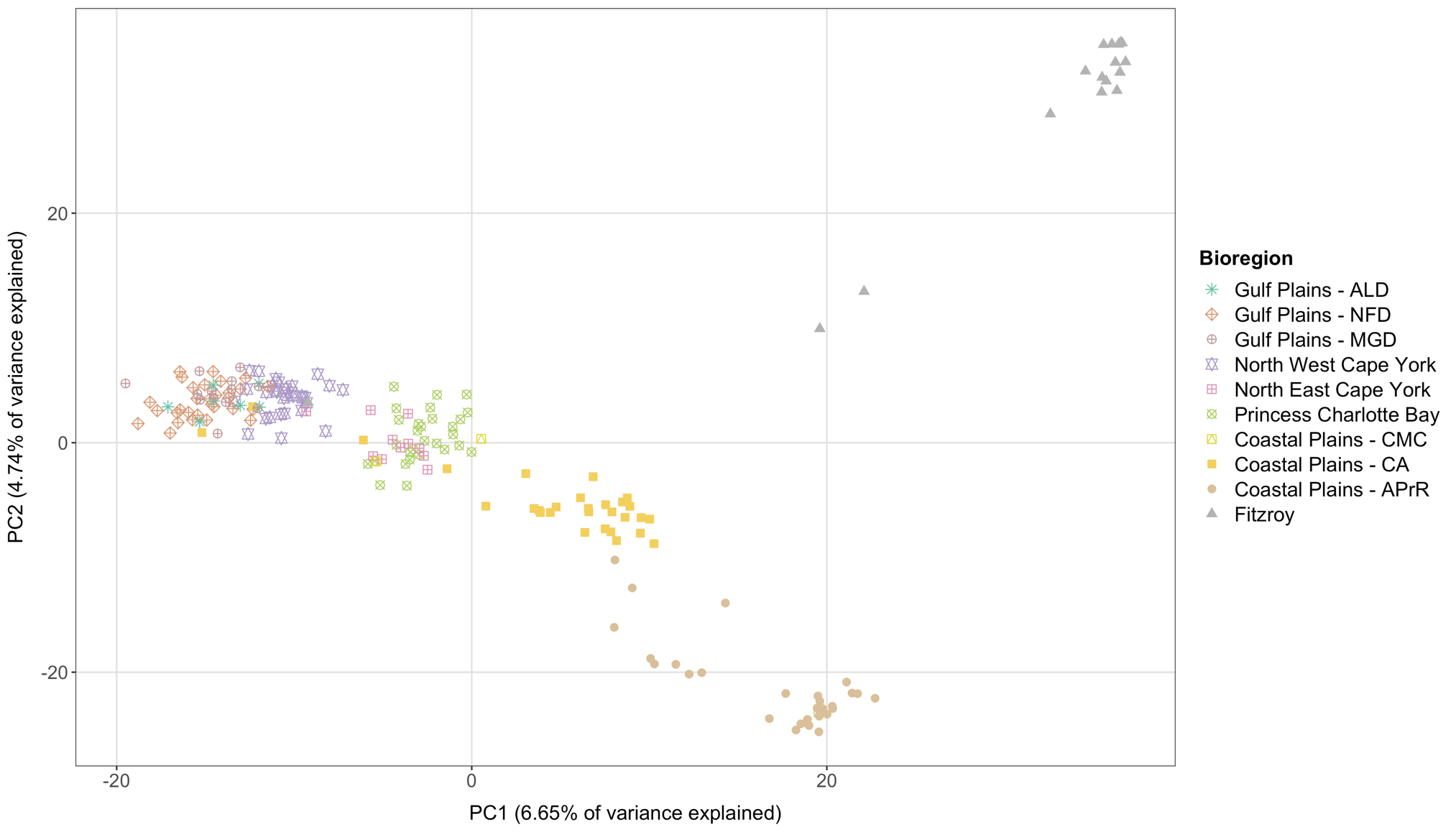


**Supplementary Figure 7 Principal component analysis (PCA) analysis summary plot from subsampling of large populations to 30 individuals.**  Bioregion abbreviations are Norman-Flinders drainage (NFD), Albert-Leichhardt drainage (ALD), Mitchell-Gilbert drainage (MGD), Cape Melville – Cooktown (CMC), Cooktown – Ayr(CA), Ayr – Proserpine – Rockhampton (AprR).


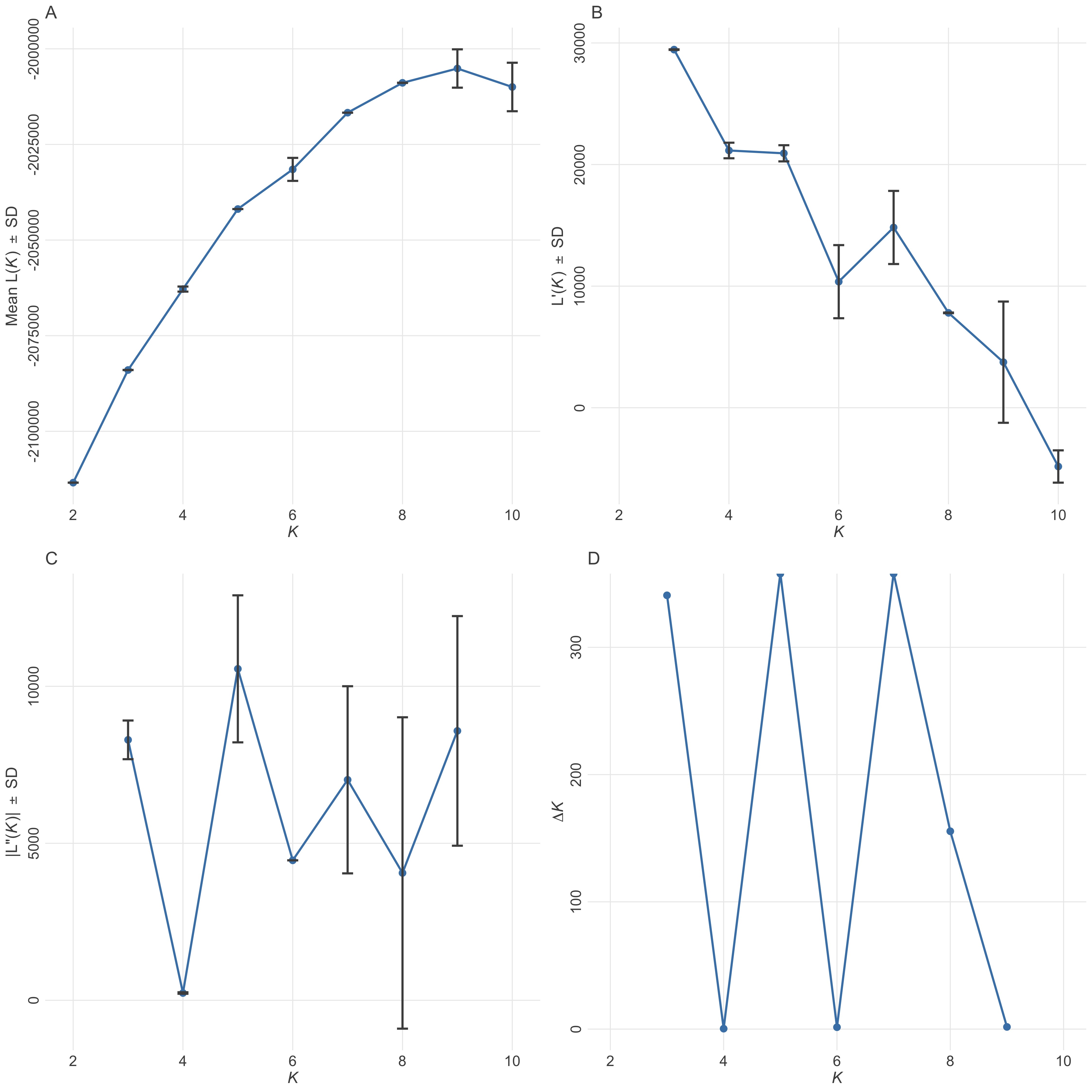


**Supplementary Figure 8 Evanno method results using STRUCTURE runs with 10,000 burn-in and 20,000 following iterations for inference.** The plot shows the Evanno analysis results with (A) estimated log probability of data of runs over increasing values of K, (B) first derivative, (C) second derivative and (D) ∆*K* over values of K.


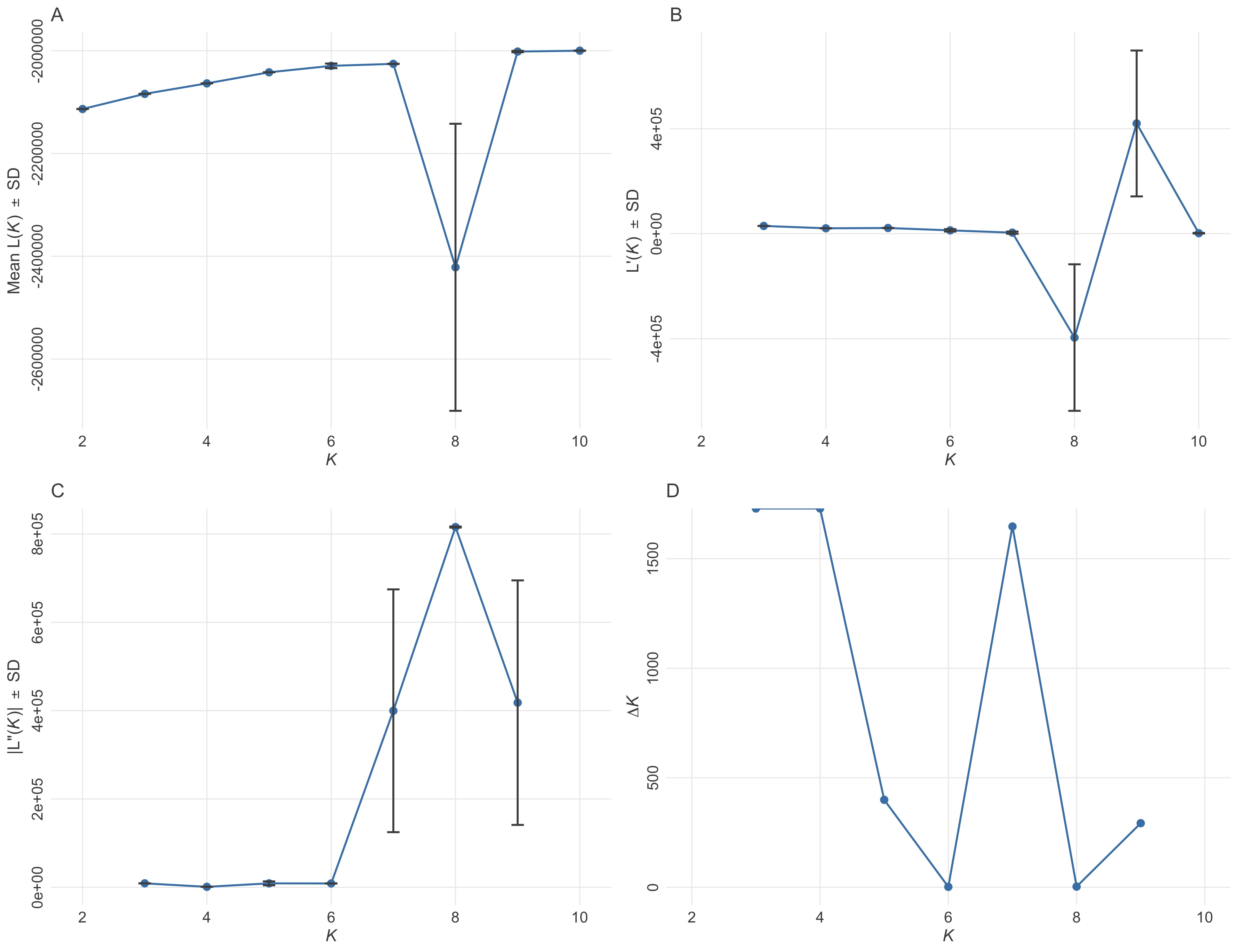


**Supplementary Figure 9 Evanno method results using STRUCTURE runs with 20,000 burn-in and 80,000 following iterations for inference.** High variance for *K* = 8 has distorted plot and thus another plot was generated in Supplementary Figure 10. The plot shows a typical Evanno analysis with (A) estimated log probability of data of runs over increasing values of K, (B) first derivative, (C) second derivative and (D) ∆K over values of K.


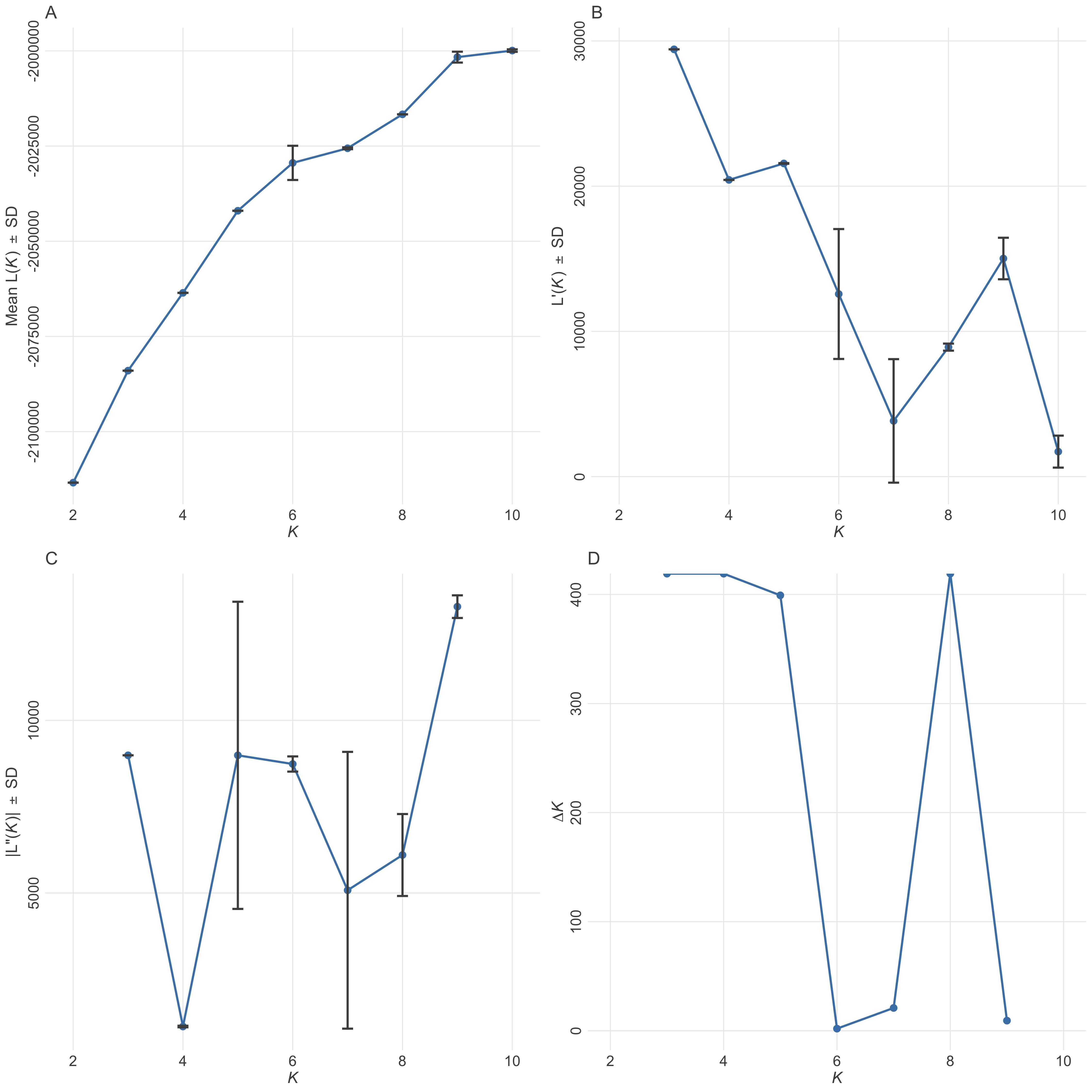


**Supplementary Figure 10 Evanno method results using STRUCTURE runs with 20,000 burn-in and 80,000 following iterations for inference.** High variance log probability of data values removed (7 of 10 runs) for *K* = 8 for comparison with other STRUCTURE results. The plot shows the Evanno analysis results with (A) estimated log probability of data of runs over increasing values of K, (B) first derivative, (C) second derivative and (D) ∆K over values of K.


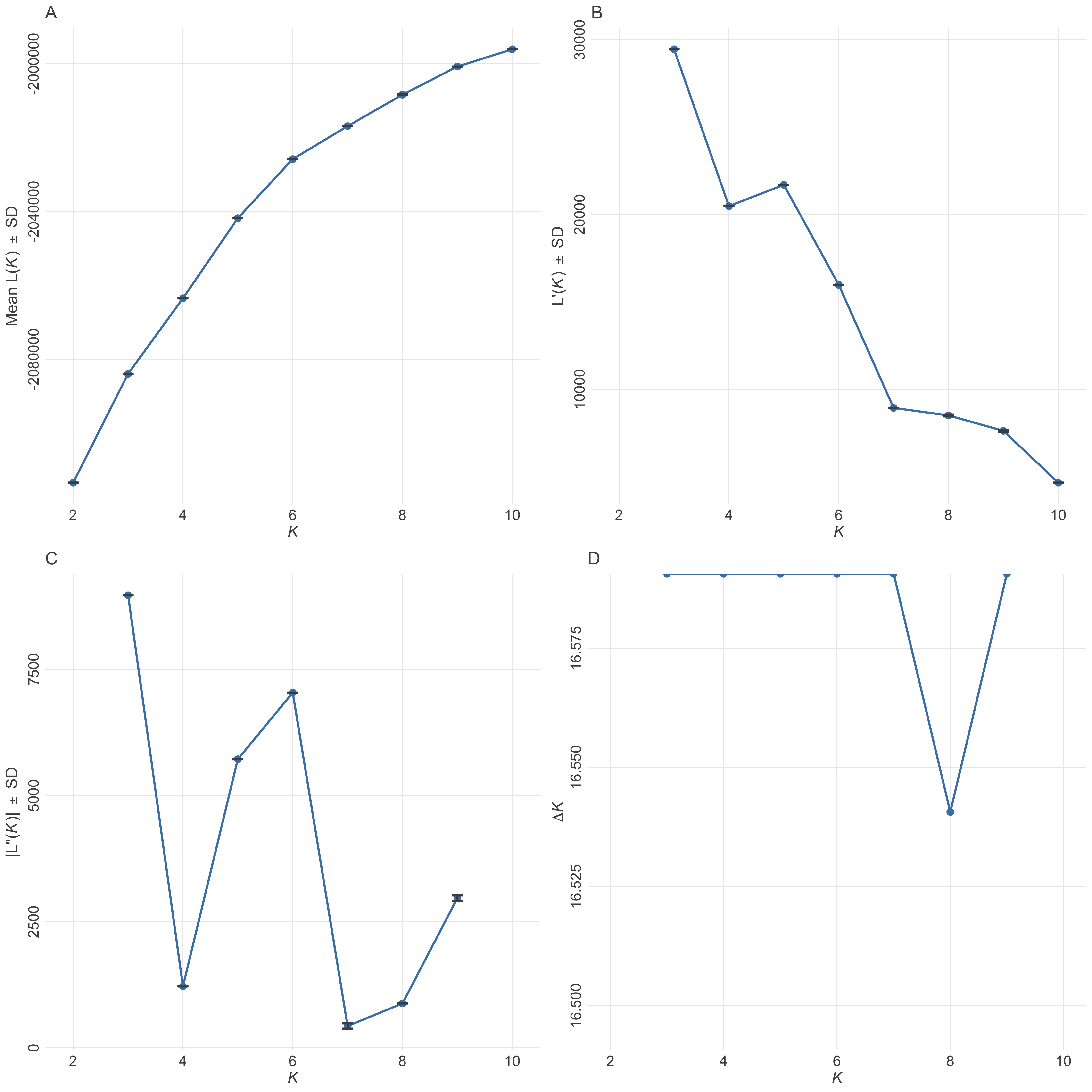


**Supplementary Figure 11 Evanno method results using STRUCTURE runs with LOCPRIOR option and location corresponding to sampled bioregions.** The plot shows the Evanno analysis results with (A) estimated log probability of data of runs over increasing values of K, (B) first derivative, (C) second derivative and (D) ∆K over values of K.


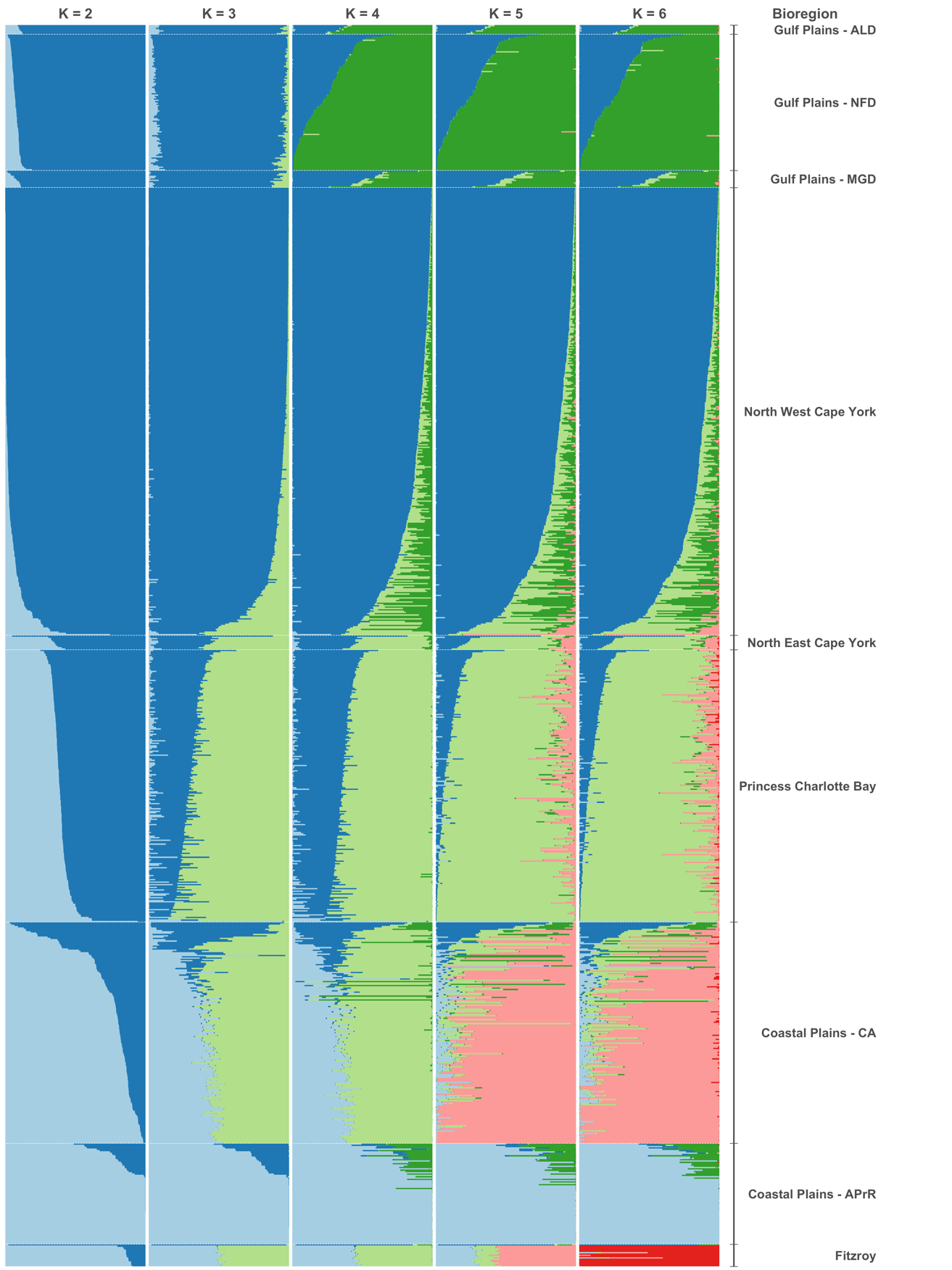


**Supplementary Figure 12 Admixture plots generated from STRUCTURE clustering analyses with LOCPRIOR option and location corresponding to sampled bioregions.** Horizontal coloured lines represent an individual sample from the bioregion indicated on the left panel. Results are based on checked convergence from five replicate runs for each K. For each K, individuals are ordered within bioregion by the major ancestry proportion. Bioregion abbreviations are Albert-Leichhardt drainage (ALD), Norman-Flinders drainage (NFD), Mitchell-Gilbert drainage (MGD), Cape Melville – Cooktown (CMC), Cooktown – Ayr (CA), Ayr – Proserpine – Rockhampton (AprR).


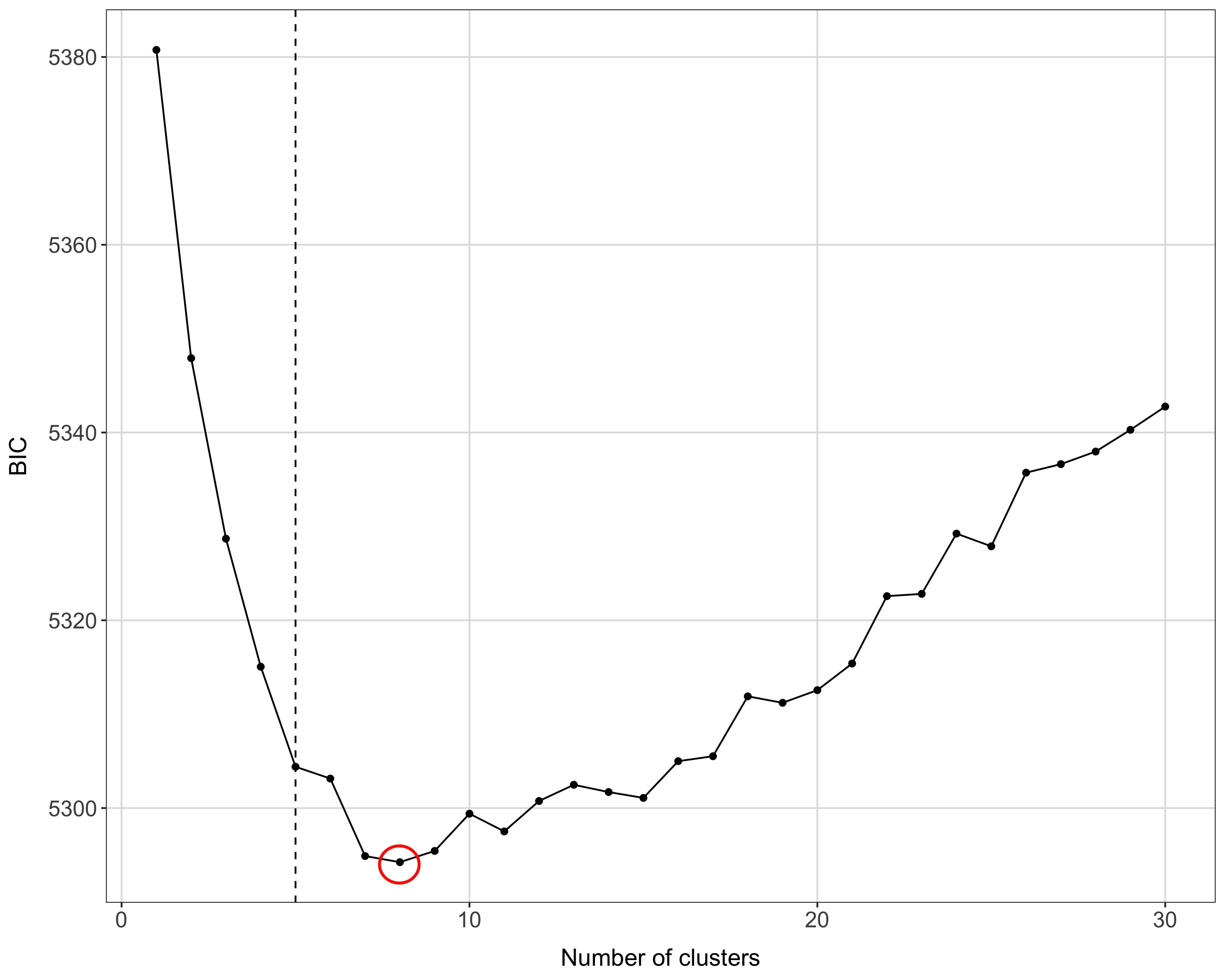


**Supplementary Figure 13 DAPC Bayesian information criterion plot for investigating the numbers of clusters in k-means algorithm.** X-axis corresponds to the number of clusters investigated and y-axis the Bayesian information criterion from the clustering algorithm for each cluster. Vertical dashed line at *K* = 5 corresponds to the first elbow in the curve. Red circle shows minimum BIC at *K* = 8.


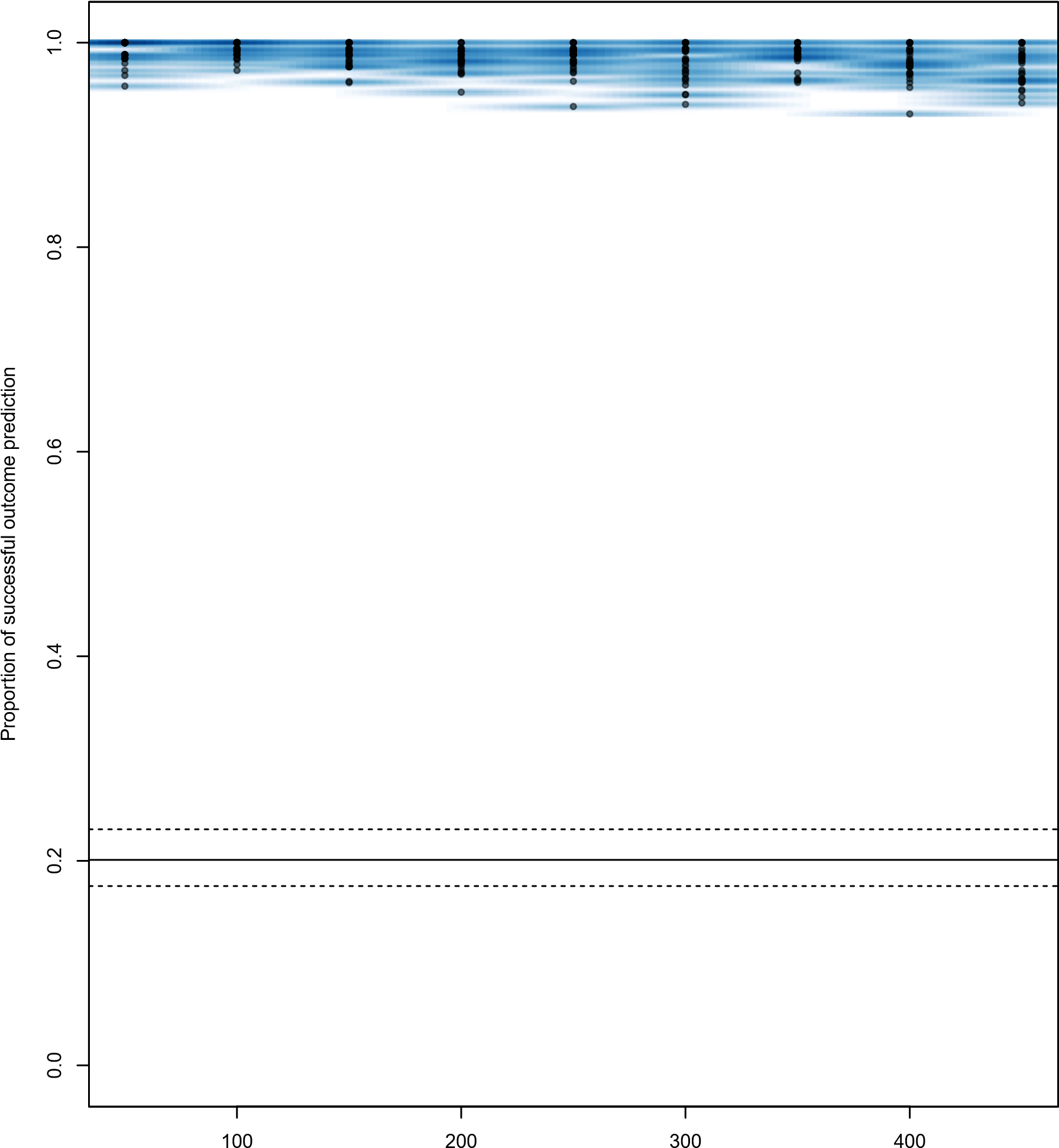


Number of PCA axes retained

**Supplementary Figure 14 Scatterplot of the DAPC cross-validation using five groups to choose the numbers of PCs for discriminant analysis.** The number of PCs retained in each DAPC varies along the x-axis, and the proportion of successful outcome prediction varies along the y-axis. Individual replicates appear as points, and the density of those points in different regions of the plot is displayed in blue. Optimal number of PCs based on mean squared error is 100 for this number of clusters.


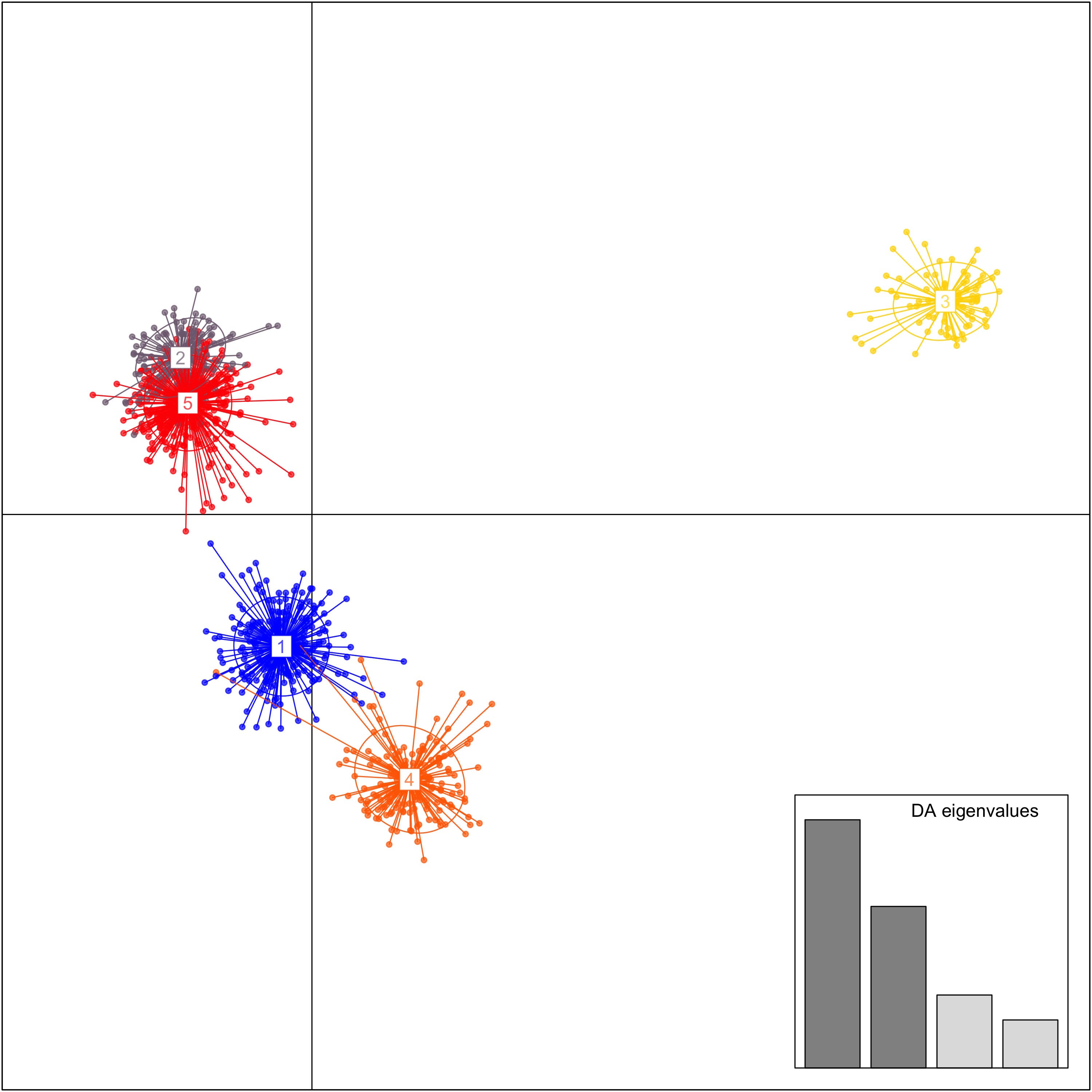


**Supplementary Figure 15 Scatterplots of the DAPC with k-means clustering analysis of saltwater crocodile genetic data.** This scatterplot shows the first two principal components of the DAPC analysis. Groups are shown by different colours and inertia ellipses, while dots represent individuals. Clusters 1 – 5 are dominated by Princess Charlotte Bay (1), Gulf Plains - NFD, ALD and MGD (2), Coastal Plains - APrR (3), Coastal Plains - CA and Fitzroy (4), North West Cape York (5) respectively. A detailed breakdown of individual’s bioregion and assigned cluster is shown in Supplementary Table 7. Inset shows scree plot of eigenvalues.


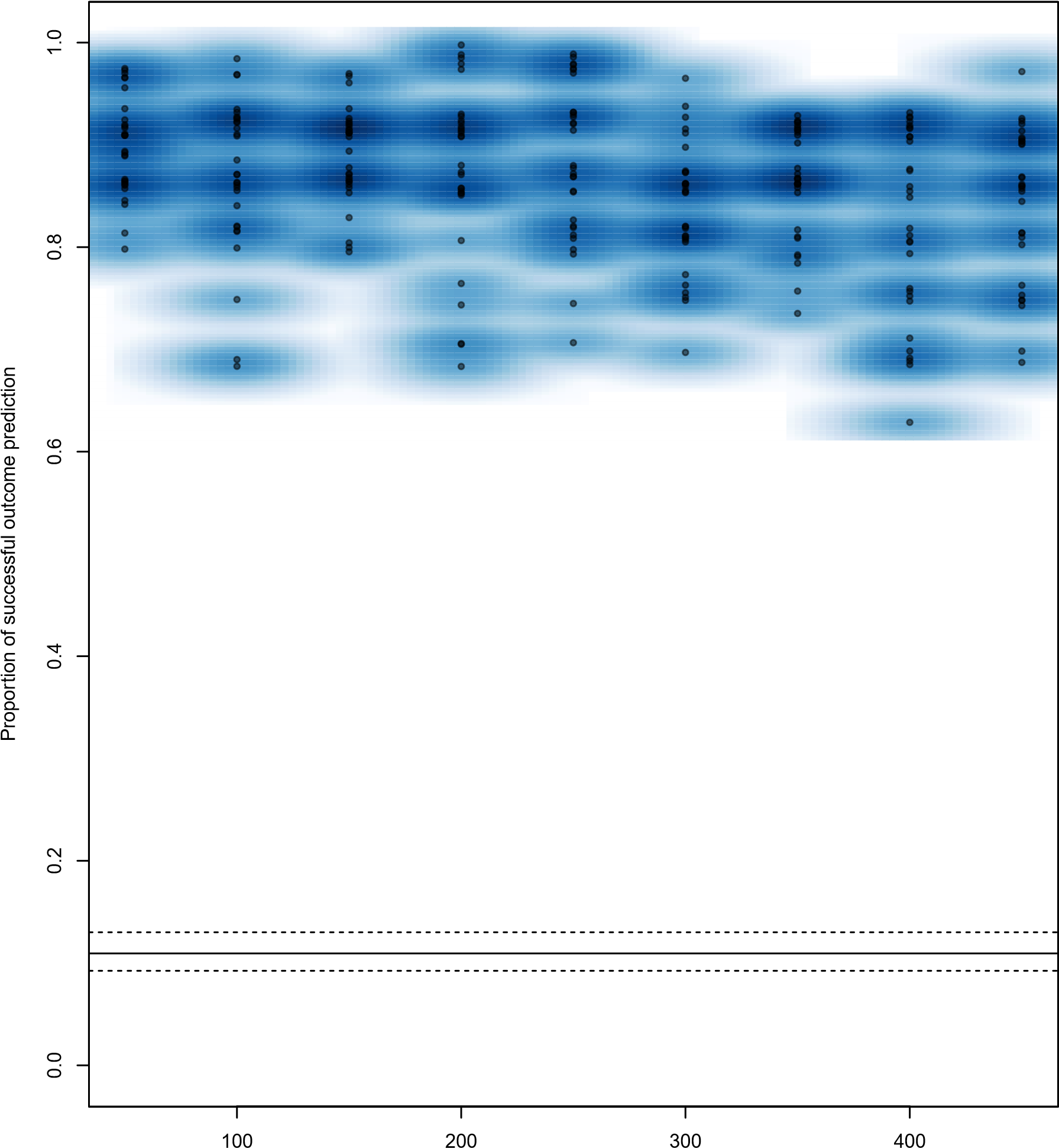


Number of PCA axes retained

**Supplementary Figure 16 Scatterplot of the DAPC cross-validation using bioregions as groups to choose the numbers of PCs for discriminant analysis.** The number of PCs retained in each DAPC varies along the x-axis, and the proportion of successful outcome prediction varies along the y-axis. Individual replicates appear as points, and the density of those points in different regions of the plot is displayed in blue. The optimal number of PCs based on mean squared error is 50 for the analysis using bioregions as clusters.


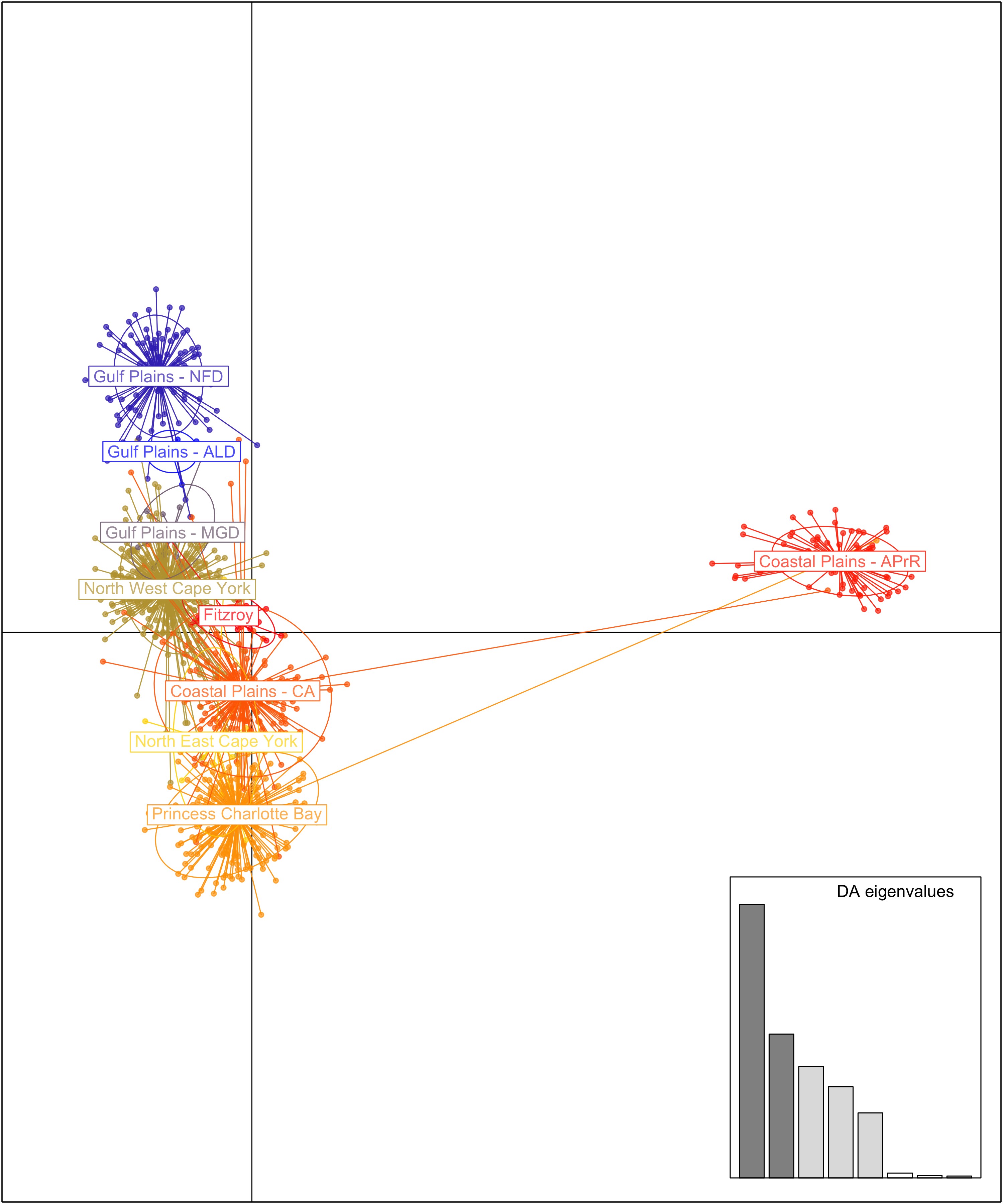


**Supplementary Figure 17 DAPC clustering scatterplot with bioregions used as the clusters**. Optimal number of PCs used for the analysis was based on mean squared error from cross-validation. 50 PCs was optimal for this number of clusters. Inset shows scree plot of eigenvalues.


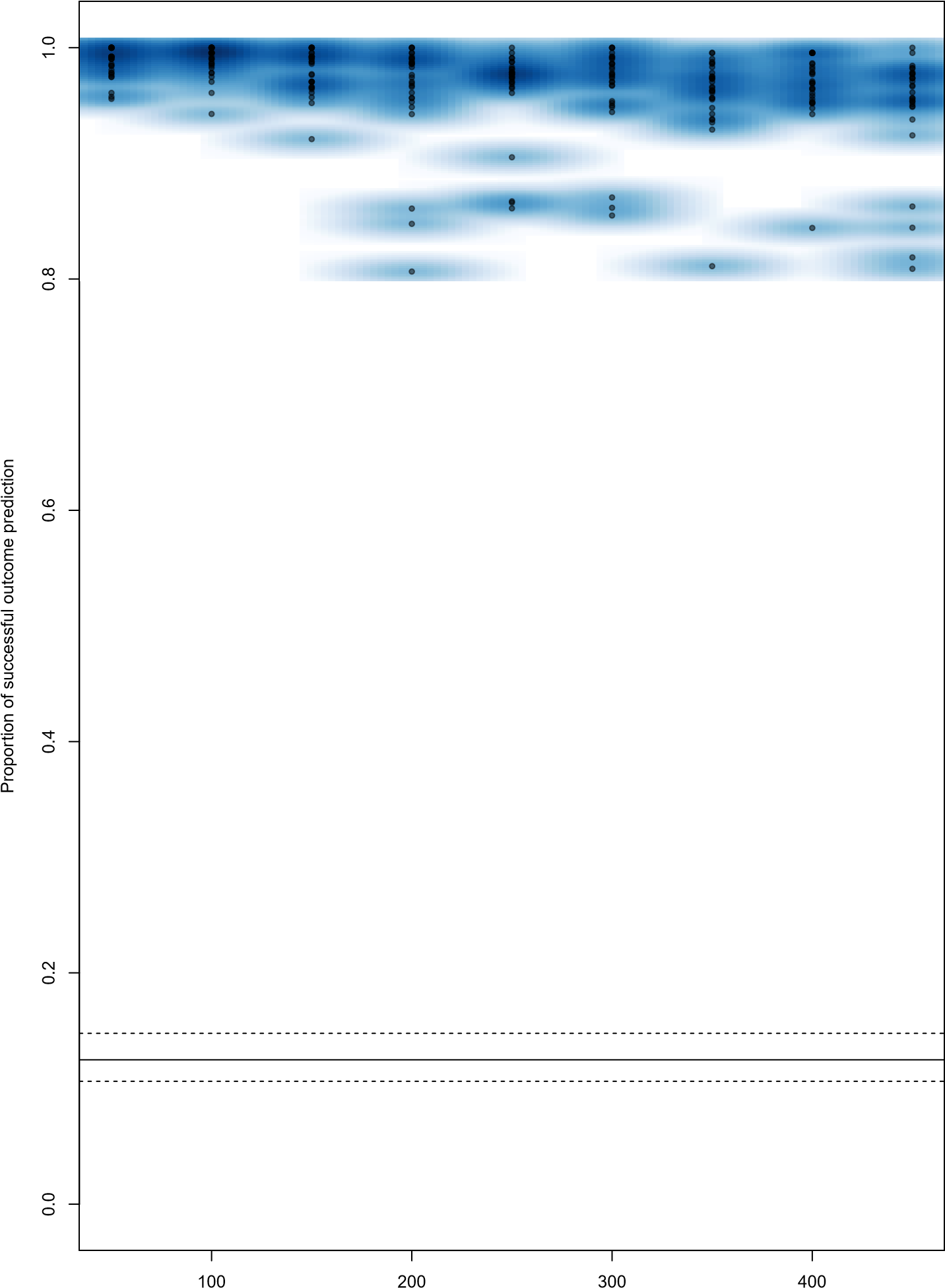


Number of PCA axes retained

**Supplementary Figure 18 Scatterplot of the DAPC cross-validation using 8 groups to choose the numbers of PCs for discriminant analysis.** The number of PCs retained in each DAPC varies along the x-axis, and the proportion of successful outcome prediction varies along the y-axis. Individual replicates appear as points, and the density of those points in different regions of the plot is displayed in blue. Optimal number of PCs based on mean squared error is 100 for this number of clusters.


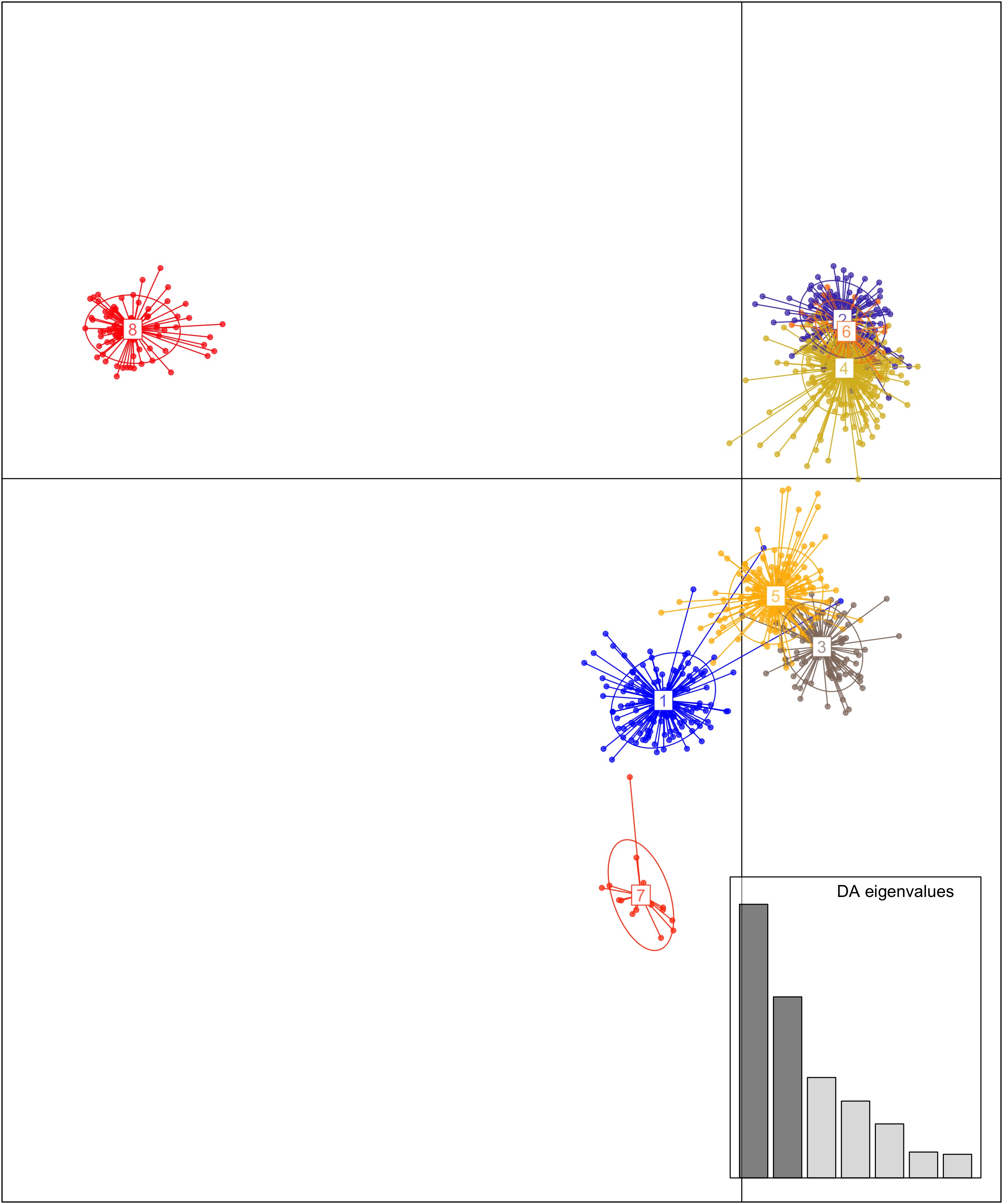


**Supplementary Figure 19 Scatterplot of the DAPC with k-means clustering analysis of saltwater crocodile genetic data using 8 clusters from the minimum BIC.** This scatterplot shows the first two discriminant functions of the DAPC analysis. Groups are shown by different colours and inertia ellipses, while dots represent individuals. The correspondence between clusters 1 – 8 and sampled bioregions are shown in Supplementary Table 8. Inset shows the scree plot of eigenvalues.


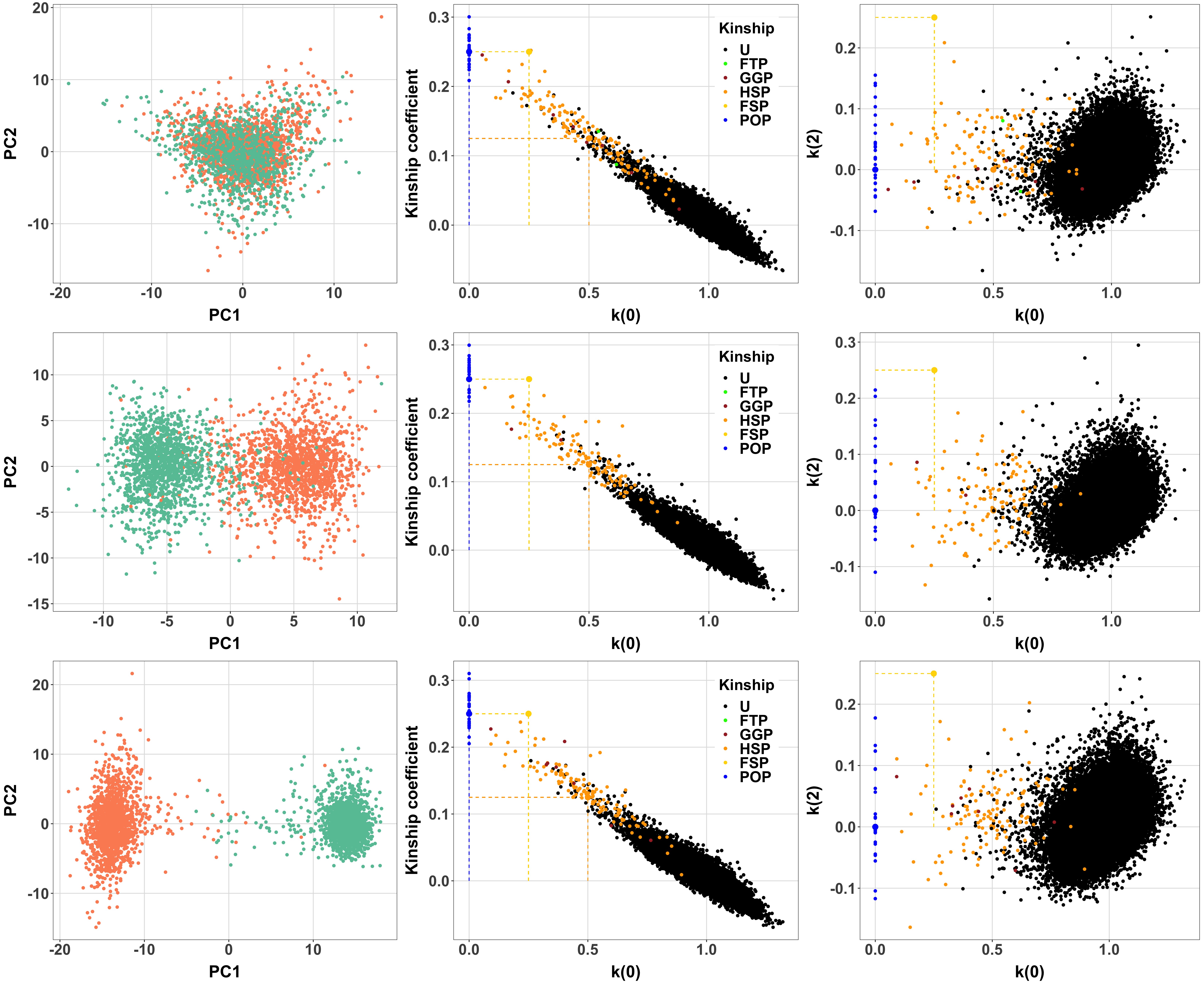


**Supplementary Figure 20 PCA and kinship statistics from forward simulation with single offspring PCA and PC-Relate kinship analyses results.** Panels in the left hand column visualise the first two principal components from the PCA analysis of all data (≈ 3,000 individuals and 2,000 SNPs) from the last generation at 1%, 0.1% and 0.01% migration per generation. The middle column shows results for the kinship coefficient and probability of sharing 0 alleles IBD (*k*(0)) estimated using PC-Relate from a random sample of 500 individuals across the two subpopulations. The righthand column shows results for the probability of sharing 2 alleles IBD (*k*(2)) versus the probability of sharing 0 alleles IBD (*k*(0)) estimated using PC-Relate from a random sample of 500 individuals across the two subpopulations.


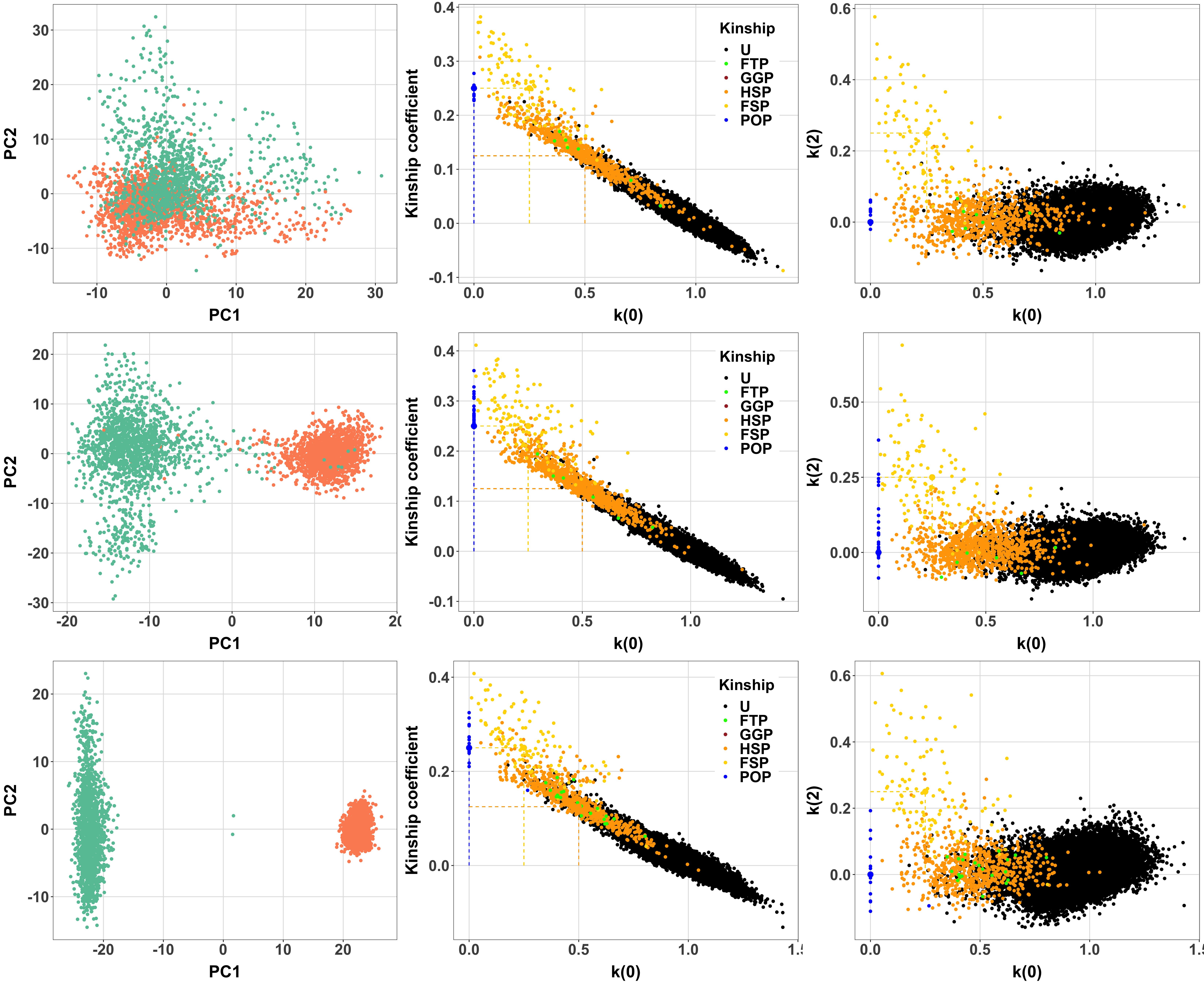


**Supplementary Figure 21 PCA and kinship statistics from forward simulation with large litters PCA and PC-Relate kinship analyses results.** Panels in the left-hand column visualise the first two principal components from the PCA analysis of all data (≈ 3,000 individuals and 2,000 SNPs) from the last generation at 1%, 0.1% and 0.01% migration per generation. The middle column shows results for the kinship coefficient and probability of sharing 0 alleles IBD (*k*(0)) estimated using PC-Relate from a random sample of 500 individuals across the two subpopulations. The right-hand column shows results for the probability of sharing 2 alleles IBD (*k*(2)) versus the probability of sharing 0 alleles IBD (*k*(0)) estimated using PC-Relate from a random sample of 500 individuals across the two subpopulations.


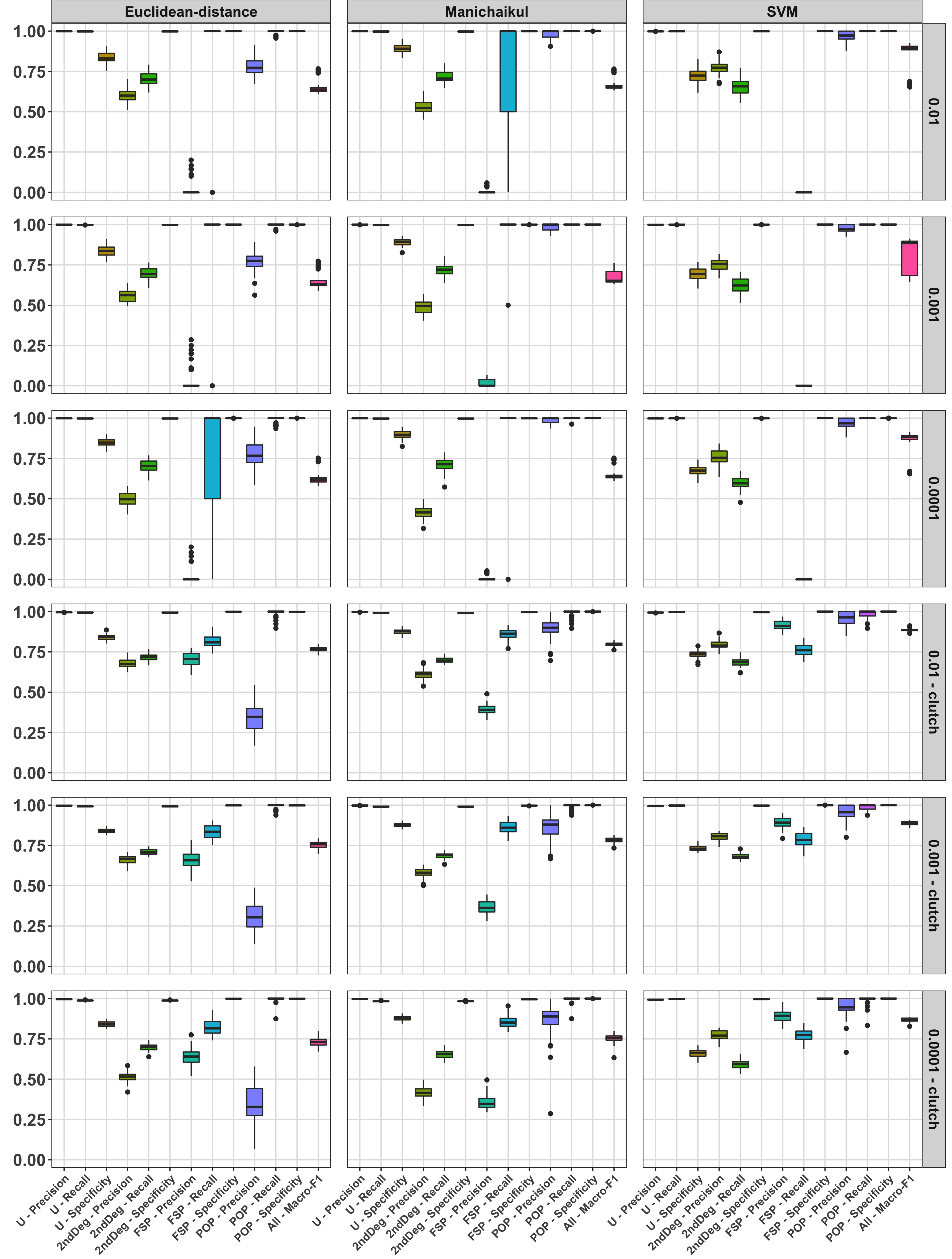


**Supplementary Figure 22 Performance of clustering methods used to determine kinship categories from forward simulation.** Results depict 45 replicate samples of 300 individuals for each simulation (rows). The Euclidean-distance clustering assigns each point by computing the distance to the expectation point (kin, k(0), k(2)) for each kinship category. The SVM is trained on a data set that consists of results from the first five replicates (total of 50 replicates was performed) and used to predict the final 45 data sets for each simulation scenario. Manichaikul corresponds to the criteria given in (Manichaikul *et al.*, 2010) (as in (Conomos *et al.*, 2016)), which for a pair of individuals is classified to have a *d*th degree relationship if their estimated kinship coefficient (*_φ_*) is in the interval (2^−(^*^d^*^+3/2)^,2^−(^*^d^*^+1/2)^). To distinguish parent-offspring from full sibling relationships, where pairs with a *k*(0) estimate less than 2^−(9/2)^ ≈ 0.044 are classified as parent-offspring. The performance measures include precision23 *TP*/(*TP*+*FP*), recall *TP*/(*TP*+*FN*), specificity *TN*/(*TN*+*FP*), and *F*1macro = 2(*P*macro*R*macro)/(*P*macro+*R*macro), where *TP* is true positive, *FP* is false positive, *FN* is false negative and *P*_macro_ and *R*_macro_ are the macro precision and recall.


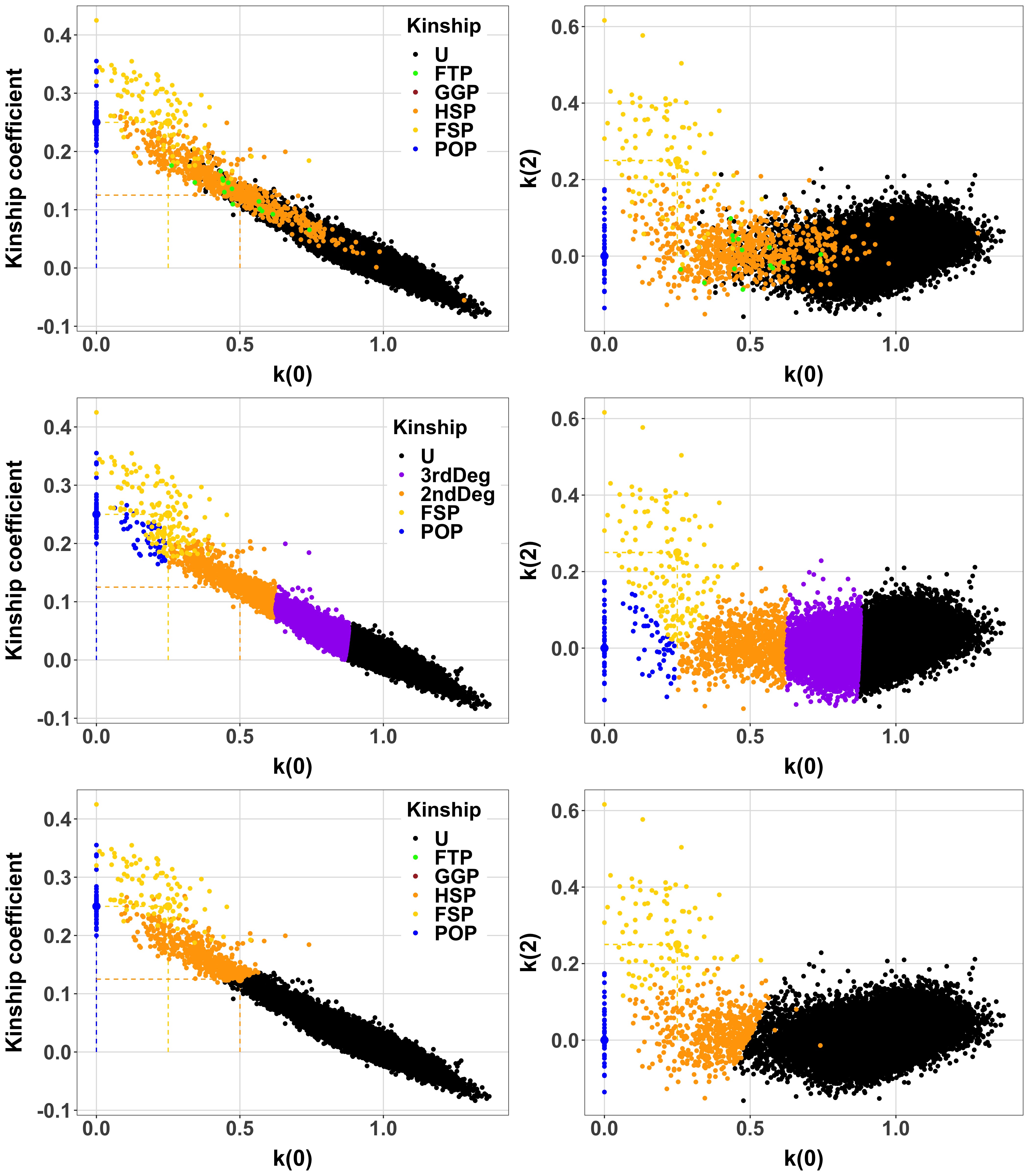


**Supplementary Figure 23 Example clustering results from one replicate of the 0.0001 - clutch forward simulation scenario.** Top row shows the true classification of the pairs of individuals. Middle row shows the classification using the Euclidean distance classification and the final row the classification using the SVM. The Euclidean distance classification used a point for 3rd-degree relatives (colour purple) to further separate the 2nd-degree relatives from the unrelated or unclassified (U) pairs. FTP corresponds to full-thiatic pairs (Aunt/Uncle-Niece/Nephew) and grandparent - grandchild pairs.


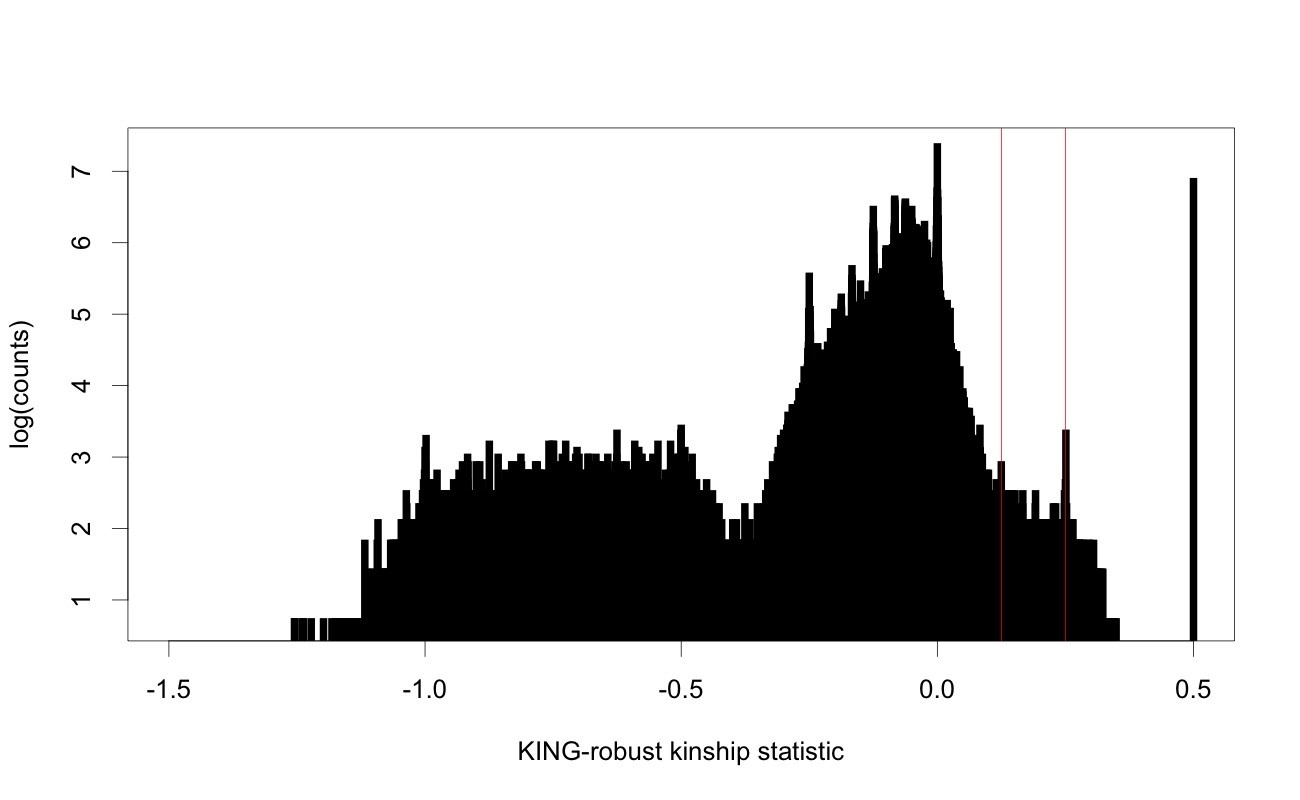
**Supplementary Figure 24 Histogram of all pairwise KING-robust kinship coefficients estimates for *C. porosus* before PC-Relate correction.** KING-robust estimates are used to differentiate related from unrelated individuals in the PC-AiR component of the PC-Relate analysis. Vertical red lines are at the 0.125 and 0.25 expectations for 2nd and 1st-degree relatives. The y-scale has been logged to allow for a better comparison of kinship coefficient regions.


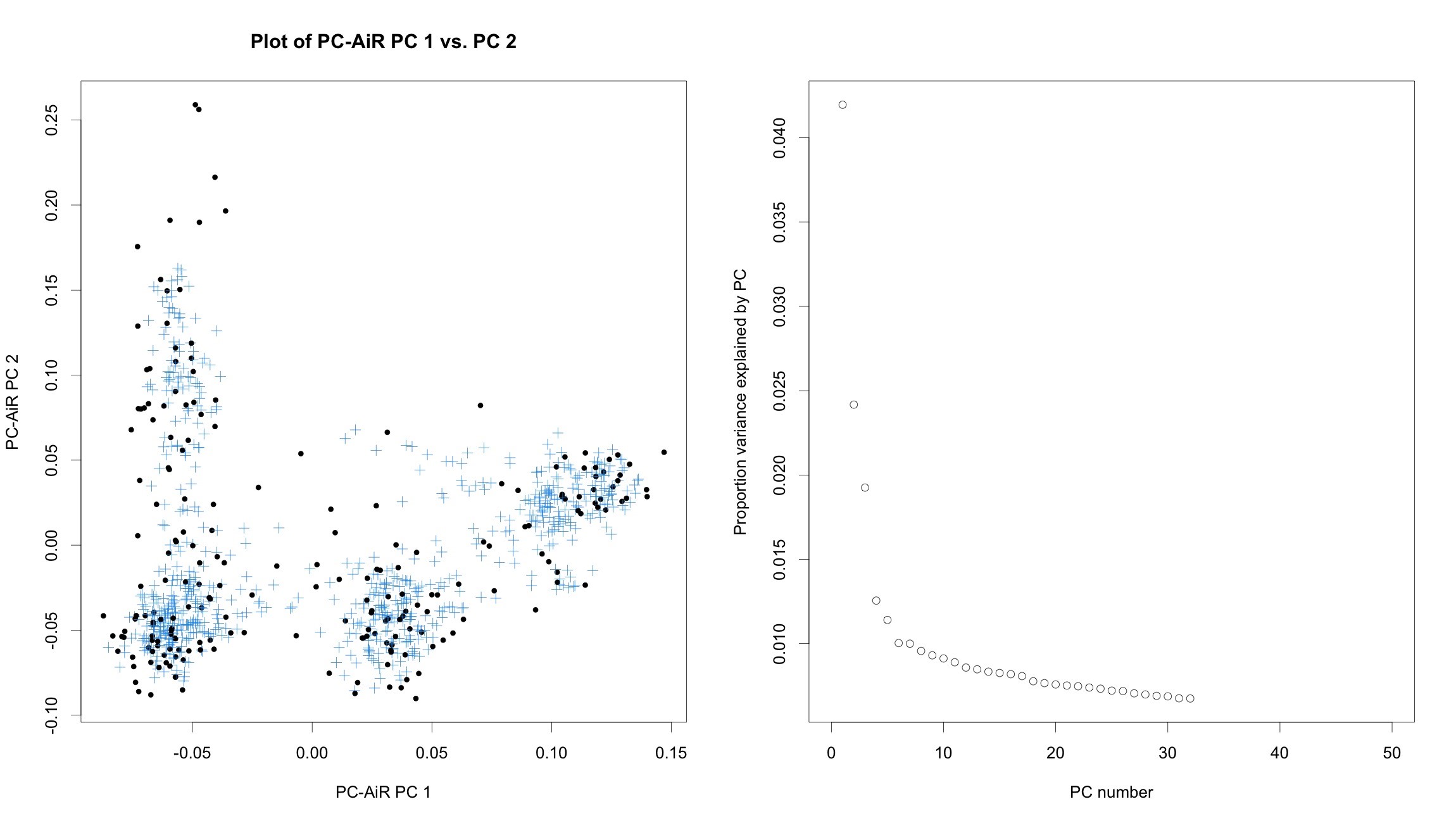
**Supplementary Figure 25 PC-AiR analysis summary for saltwater crocodile population.** PC-AiR partitions the sample of individuals into an ancestry representative 'unrelated subset’ (black dots) and a 'related set’ (blue crosses) and performs standard PCA on the 'unrelated subset’ and predicts PC values for the 'related subset’.


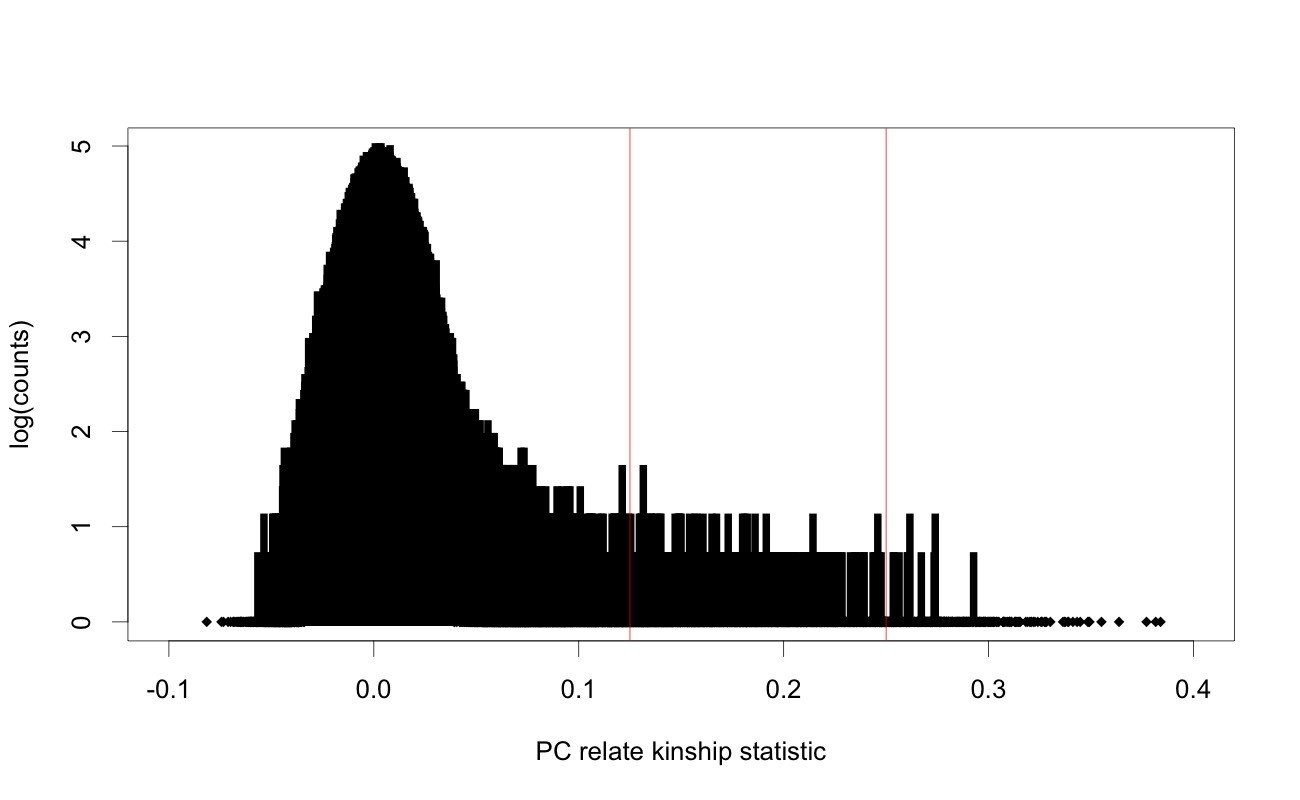
**Supplementary Figure 26 PC-Relate kinship statistics post correction for structure.** Presented for comparison with Supp. Figure 24 and shows the influence of genetic structure on the KING-robust estimates and the correction that PC-Relate achieved. Vertical red lines are at the 0.125 and 0.25 expectations for 2^nd^ and 1^st^-degree relatives. The y-scale has been logged to allow for a better comparison of kinship coefficient regions.


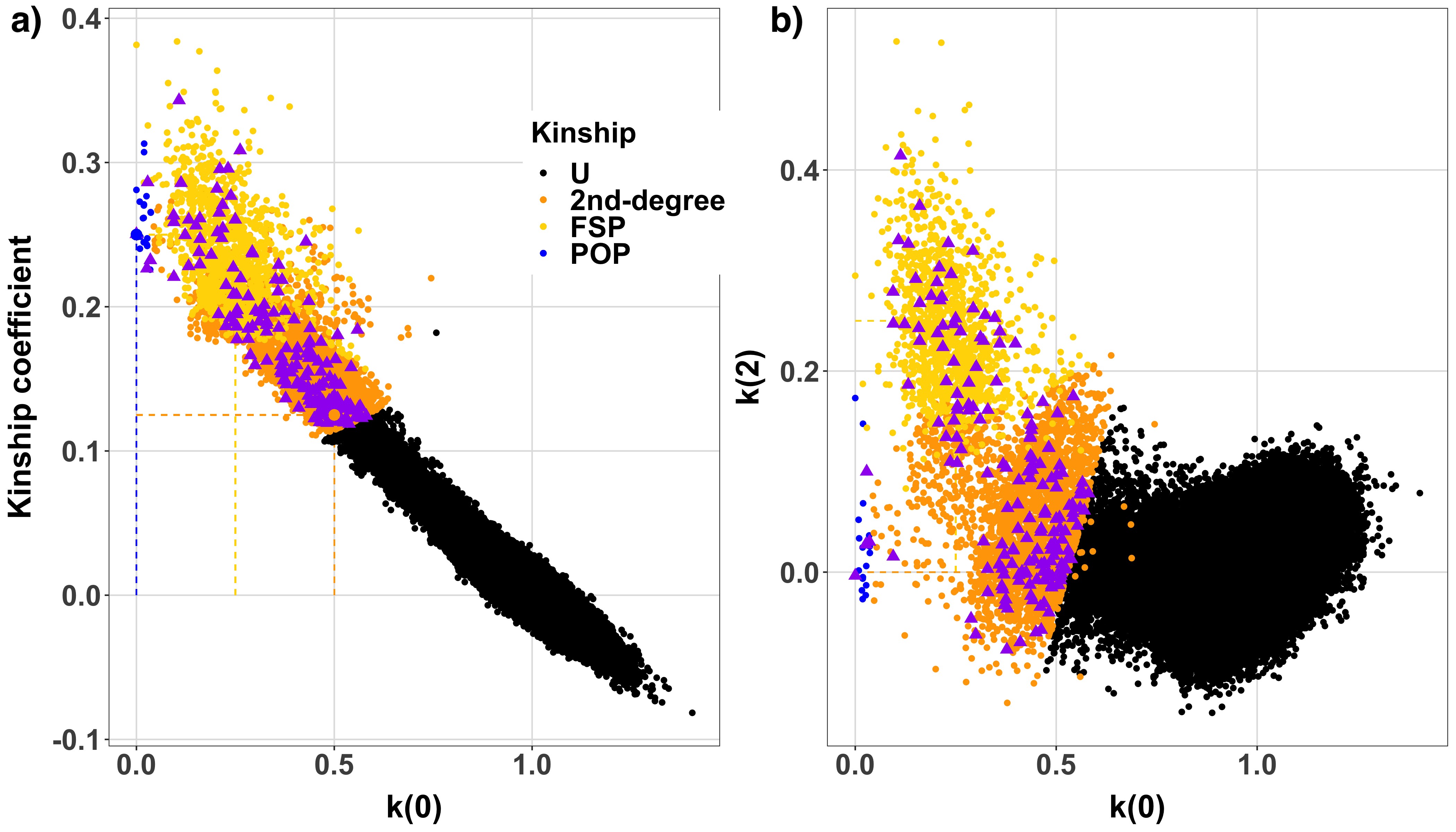


**Supplementary Figure 27 Scatterplots of the kinship coefficients and statistics from PC-Relate analysis of saltwater crocodile population with across bioregion kin marked.** Panels a) and b) show the kinship coefficient, *k*(0) and *k*(2) distribution estimate for all pairs from the PC-Relate analysis and the classification into parent-offspring pairs (POP), full-sibling pairs (FSP) and 2nd-degree relatives including potentially halfsibling, grandparent/grandchild and full-thiatic pairs. The purple triangles show the pairs that are across bioregion and classified as POPs, FSPs or 2nd-degree relatives. The intention of the plot is to assess the distribution of the across bioregion kin to ensure they are not all at the threshold of kin types. Dashed lines show the expected values for each of the statistics. The bottom figure displays the scatterplot of the kinship coefficient versus the geographic distance between each pair and the classification for each pair.


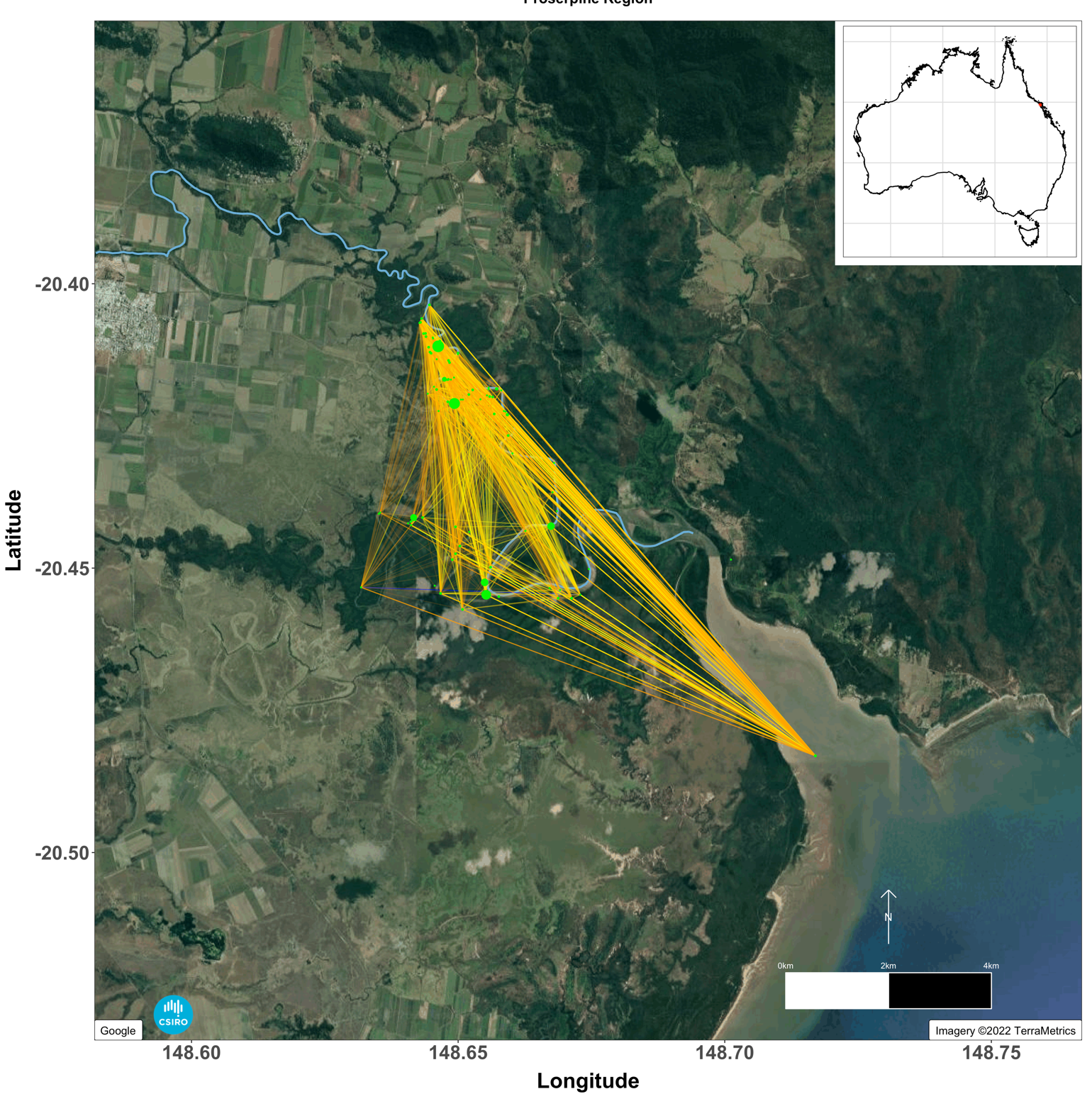


**Supplementary Figure 28 Summary of kinship connections between individuals sampled in the Proserpine River.** The Proserpine River (blue) is the main contributor in terms of total samples (112/113) to the Coastal Plains - APrR bioregion. Each green dot depicts a location where an individual crocodile was sampled with the size of the dot indicating the relative size of the individual sampled i.e., larger dots imply larger crocodiles. The line joining each dot indicates that the pair is classified as related, and the colour of the line indicates the relationship type. Blue are POPs, yellow FSPs, and orange HSPs. The red inset box shows the region of Australia plotted.


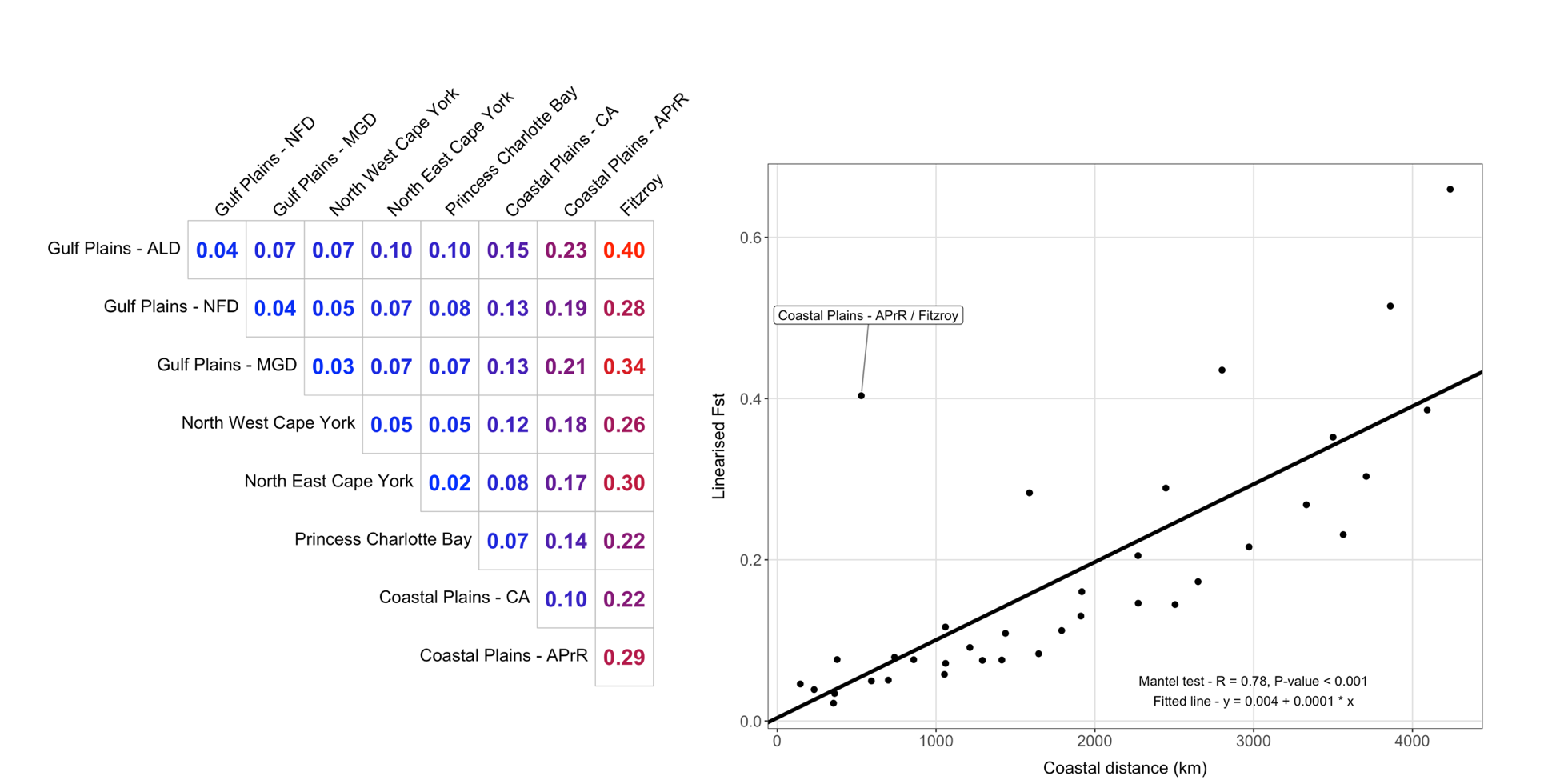


**Supplementary Figure 29 Pairwise FST values and correlation of genetic and geographic distance between all bioregions calculated from 910 crocodiles with possible translocated individuals removed.** All entries in a) are significant with bootstrap (replicates = 1,000) p-values less than the FDR adjusted threshold of 0.05. Row and columns in panel a) are ordered by position along the Queensland coast from west to east. Bioregion abbreviations are Albert-Leichhardt drainage (ALD), Norman-Flinders drainage (NFD), Mitchell-Gilbert drainage (MGD), Cape Melville – Cooktown (CMC), Cooktown – Ayr (CA), Ayr – Proserpine – Rockhampton (APrR). Panel b) shows the positive correlation between coastal distance and Slatkin's linearized F*_ST_*. The black line indicates the fitted regression line with the coefficients detailed in the figure. The Mantel correlation coefficient and its associated significance are also shown. The point comparing the Coastal Plains - APrR and Fitzroy bioregions is highlighted.


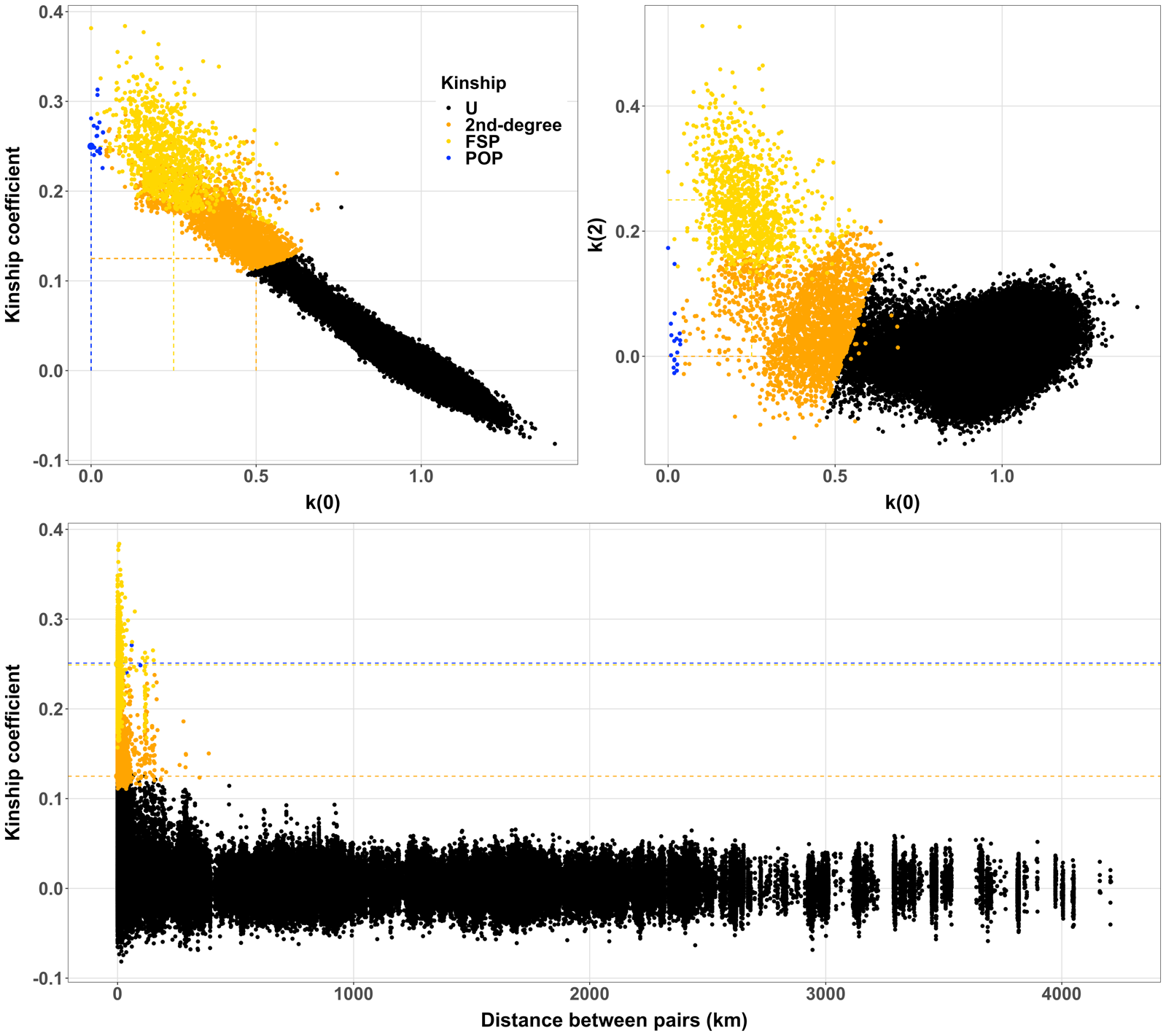


**Supplementary Figure 30 Scatterplots of the kinship coefficients and statistics from PC-Relate analysis of saltwater crocodile population with possible translocations removed.** Panels a) and b) show the kinship coefficient, k(0) and k(2) distribution estimate for all pairs from the PC-Relate analysis and the classification into parent-offspring (POP), full-sibling (FSP) and 2nd-degree relatives including potentially half-sibling, grandparent/grandchild and full-thiatic pairs. Dashed lines show the expected values for each of the statistics. Panel c) displays the scatterplot of the kinship coefficient versus the geographic distance be- tween each pair along with the classification for each pair. The geographic distance is the distance along the coastline between pairs in different bioregions. For pairs within a bioregion, the distance is the straight-line distance between their sampling locations. The horizontal lines show the expectation with the POP and FSP lines slightly jitter of 0.25 so both can be shown.

## Supplementary Tables

SWC bioregion Main sampled region Number of samples

| Fitzroy  Coastal Plains - APrR | Fitzroy River | 23 |
| --- | --- | --- |
|  | Proserpine | 105 |
|  | Sarina | 1 |
| Coastal Plains - CA | Cooktown | 2 |
|  | Bloomfield | 5 |
|  | Cairns region | 133 |
|  | Innisfail | 39 |
|  | Cardwell | 4 |
|  | Mission Beach | 3 |
|  | Kurramine | 1 |
|  | Townsville | 4 |
|  | Whitsunday | 3 |
|  | Edmund Kennedy | 63 |
| Coastal Plains - CMC  Princess Charlotte Bay | Lizard Island | 2 |
| Lakefield | | 239 |
| North-East Cape York | |  |
| Lockart/ Claudie | | 11 |
| Jackey Jackey Ck | | 1 |
| North-West Cape York  Pompuraaw | |  |
| Pompuraaw | | 139 |
| Wenlock River | | 84 |
| Tentpole Creek | | 163 |
| Weipa | | 4 |
| Torres Strait | | 15 |
| Gulf Plains - MGD | |  |
| Staaten River  Gulf Plains - NFD | | 13 |
| Norman River | | 64 |
| Walkers Creek | | 45 |
| Jenny Lind River | | 2 |
| Gulf Plains - ALD Albert River | | 8 |
| Total | | 1176 |

**Supplementary Table 1 Summary of numbers of saltwater crocodiles sampled across bioregions.** Grey rows correspond to the regions that were subsampled and used in the DArTSeq pilot analysis. For the pilot analysis 41, 39, 38, 31 and 39 (n = 188) samples were taken from Cairns, Lakefield, Norman River, Proserpine, and Wenlock respectively.

Size class Feet Metres

| 1 | 1-2 | 0.30 – 0.61 |
| --- | --- | --- |
| 2 | 2-3 | 0.62 – 0.91 |
| 3 | 3-4 | 0.92 – 1.22 |
| 4 | 4-5 | 1.23 – 1.52 |
| 5 | 5-6 | 1.53 – 1.83 |
| 6 | 6-7 | 1.84 – 2.13 |
| 7 | 7-8 | 2.14 – 2.44 |
| 8 | 8-9 | 2.45 – 2.74 |
| 9 | 9-10 | 2.75 – 3.05 |
| 10 | 10-11 | 3.06 – 3.35 |
| 11 | 11-12 | 3.36 – 3.66 |
| 12 | 12-13 | 3.67 – 3.96 |
| 13 | 13-14 | 3.97 – 4.27 |
| 14 | 14-15 | 4.28 – 4.57 |
| 15 | 15-16 | 4.58 – 4.88 |
| 16 | >16 | >4.89 |

**Supplementary Table 2 Summary of saltwater crocodile size-class categories and their mapping to length.** Size classes are available for most of the individuals present in the data set as meta data.

Gulf Plains

Cape York

Size Class

Eastern Coastal Plains North South North-east North-west Princess Charlotte Bay Torres Strait Total

1

8

1

23

5

150

57

413

169

2

0

216

4

63

29

13

58

49

3

21

13

7

37

0

93

15

0

4

4

51

15

8

1

0

15

8

5

2

18

4

0

56

3

23

6

6

2

0

0

18

32

5

6

1

7

6

0

5

0

2

24

37

0

8

14

0

3

12

0

3

3

35

9

4

0

1

15

1

0

2

23

10

0

1

0

0

8

0

9

0

11

0

0

0

1

0

11

10

0

12

6

0

1

0

0

1

8

0

13

4

0

1

0

0

0

0

5

14

0

0

0

2

0

0

2

4

15

0

0

0

0

2

0

0

2

16

0

0

1

0

1

0

0

2

**Supplementary Table 3 Summary of sampled saltwater crocodiles in each size class categorised by bioregion.** Not all individuals had size class information and thus the grand total is 997 (subset of 1,176) individuals over this table.

33

| FILTERS | PARAMETERS | VALUES | BEFORE | AFTER | Removed |
| --- | --- | --- | --- | --- | --- |
| DArT reproducibility | filter.reproducibility | 0.014 | 1297 / 10 / 6125 / 6760 | 1297 / 10 / 6125 / 6760 | 0 / 0 / 0 / 0 |
| Monomorphic markers | filter.monomorphic |  | 1297 / 10 / 6125 / 6760 | 1297 / 10 / 6123 / 6758 | 0 / 0 / 2 / 2 |
| Individuals based on missingness (outlier stats) | filter.individuals.missing | 0.0947015 | 1297 / 10 / 6123 / 6758 | 1189 / 10 / 6123 / 6758 | 108 / 0 / 0 / 0 |
| Monomorphic markers | filter.monomorphic |  | 1189 / 10 / 6123 / 6758 | 1189 / 10 / 5096 / 5416 | 0 / 0 / 1027 / 1342 |
| MAC | filter.mac | 5 | 1189 / 10 / 5096 / 5416 | 1189 / 10 / 3924 / 4094 | 0 / 0 / 1172 / 1322 |
| Coverage min / max | filter.coverage | 10 / 200 | 1189 / 10 / 3924 / 4094 | 1189 / 10 / 3185 / 3327 | 0 / 0 / 739 / 767 |
| Genotyping | filter.genotyping | 0.3 | 1189 / 10 / 3185 / 3327 | 1189 / 10 / 3115 / 3232 | 0 / 0 / 70 / 95 |
| SNPs position on the read | filter.snp.position.read | all | 1189 / 10 / 3115 / 3232 | 1189 / 10 / 3115 / 3232 | 0 / 0 / 0 / 0 |
| Markers snp number | filter.snp.number | 1 | 1189 / 10 / 3115 / 3232 | 1189 / 10 / 3000 / 3000 | 0 / 0 / 115 / 232 |
| Detect mixed genomes | ind.heterozygosity.threshold (min/max) | 0 0.37 | 1189 / 10 / 3000 / 3000 | 1165 / 10 / 3000 / 3000 | 24 / 0 / 0 / 0 |
| Filter monomorphic markers | filter.monomorphic |  | 1165 / 10 / 3000 / 3000 | 1165 / 10 / 3000 / 3000 | 0 / 0 / 0 / 0 |
| detect duplicate genomes | dup.threshold | 0.125 | 1165 / 10 / 3000 / 3000 | 1040 / 10 / 3000 / 3000 | 125 / 0 / 0 / 0 |
| Filter monomorphic markers | filter.monomorphic |  | 1040 / 10 / 3000 / 3000 | 1040 / 10 / 3000 / 3000 | 0 / 0 / 0 / 0 |
| Filter HWE | hw.pop.threshold/ midp.threshold | 3/0.001 | 1040 / 10 / 3000 / 3000 | 1040 / 10 / 2958 / 2958 | 0 / 0 / 42 / 42 |

**Supplementary Table 4 Summary of filtering steps and loci removed from Radiator quality control of DArTCap 'counts format' data set.** The steps are a subset of those detailed in the Radiator R package for the filter_rad() function. The outlier statistic for reproducibility corresponds to the final variant set having scores > 0.9. The before, after and removed columns show the number of individuals/strata/loci/variants before and after the filters were applied and the numbers removed to facilitate comparison. The MAC corresponds to an approximate MAF filter of 0.002. A further 91 individuals were removed based on low heterozygosity after this filtering process, as within population filtering cannot be performed in the filter_rad() pipeline. A further individual was removed based on location provenance leaving 948 individuals for the main analyses.

| FILTERS | PARAMETERS | VALUES | BEFORE | AFTER | Removed |
| --- | --- | --- | --- | --- | --- |
| DArT reproducibility | filter.reproducibility | 0.95 | 1297 / 10 / 6125 / 6760 | 1297 / 10 / 5866 / 6439 | 0 / 0 / 259 / 321 |
| Monomorphic markers | filter.monomorphic |  | 1297 / 10 / 5866 / 6439 | 1297 / 10 / 5864 / 6437 | 0 / 0 / 2 / 2 |
| Individuals based on missingness (outlier stats) | filter.individuals.missing | 0.0955 | 1297 / 10 / 5864 / 6437 | 1199 / 10 / 5864 / 6437 | 98 / 0 / 0 / 0 |
| Monomorphic markers | filter.monomorphic |  | 1199 / 10 / 5864 / 6437 | 1199 / 10 / 4821 / 5102 | 0 / 0 / 1043 / 1335 |
| MAC | filter.mac | 1 | 1199 / 10 / 4821 / 5102 | 1199 / 10 / 4821 / 5102 | 0 / 0 / 0 / 0 |
| Genotyping | filter.genotyping | 0.3 | 1199 / 10 / 4821 / 5102 | 1199 / 10 / 4594 / 4828 | 0 / 0 / 227 / 274 |
| SNPs position on the read | filter.snp.position.read | all | 1199 / 10 / 4594 / 4828 | 1199 / 10 / 4594 / 4828 | 0 / 0 / 0 / 0 |
| Markers snp number | filter.snp.number | 1 | 1199 / 10 / 4594 / 4828 | 1199 / 10 / 4367 / 4367 | 0 / 0 / 227 / 461 |
| Detect mixed genomes | ind.heterozygosity.threshold (min/max) | 0 0.28 | 1199 / 10 / 4367 / 4367 | 1180 / 10 / 4367 / 4367 | 19 / 0 / 0 / 0 |
| Filter monomorphic markers | filter.monomorphic |  | 1180 / 10 / 4367 / 4367 | 1180 / 10 / 4348 / 4348 | 0 / 0 / 19 / 19 |
| detect duplicate genomes | dup.threshold | 0.125 | 1180 / 10 / 4348 / 4348 | 1052 / 10 / 4348 / 4348 | 128 / 0 / 0 / 0 |
| Filter monomorphic markers | filter.monomorphic |  | 1052 / 10 / 4348 / 4348 | 1052 / 10 / 4314 / 4314 | 0 / 0 / 34 / 34 |
| Filter HWE | hw.pop.threshold/ midp.threshold | 8/0.01 | 1052 / 10 / 4314 / 4314 | 1052 / 10 / 4313 / 4313 | 0 / 0 / 1 / 1 |

**Supplementary Table 5 Summary of filtering steps and loci removed from Radiator quality control of DArTCap 'counts format' data set with light filtering on MAC and HWE.** The steps are a subset of those detailed in the Radiator R package for the filter_rad() function. The outlier statistic for reproducibility corresponds to the final variant set having scores > 0.9. The before, after and removed columns show the number of individuals/strata/loci/variants before and after the filters were applied and the numbers removed to facilitate comparison. The MAC corresponds to an approximate MAF filter of 0.002. A further 91 individuals were removed based on low heterozygosity after this filtering process, as within population filtering cannot be performed in the filter_rad() pipeline. A further individual was removed based on location provenance leaving 948 individuals for the main analyses.

| ALD | | NFD | MGD | North West | North East | Charlotte Bay CA | | APrR | Fitzroy |
| --- | --- | --- | --- | --- | --- | --- | --- | --- | --- |
| *N* 7 | | 105 | 13 | 346 | 11 | 208 | 175 | 77 | 17 |
| *M*_0_ 1744 | | 1021 | 1459 | 712 | 1472 | 841 | 884 | 1542 | 1986 |
| MAF *<* 1% 1744 | | 1233 | 1459 | 1209 | 1472 | 1249 | 1194 | 1850 | 1986 |
| *A_r_* | 1.410 | 1.490 | 1.450 | 1.490 | 1.460 | 1.490 | 1.480 | 1.380 | 1.290 |
| *H_O_* | 0.172 | 0.179 | 0.177 | 0.173 | 0.175 | 0.179 | 0.169 | 0.173 | 0.108 |
| *uH_E_* | 0.169 | 0.181 | 0.176 | 0.179 | 0.181 | 0.182 | 0.179 | 0.146 | 0.106 |
| *F_IS_*  *F_IS_* - CI | -0.091 0.017 -0.041 0.047 -0.019 0.026 0.067 -0.123 0.016  (-0.256, -0.090) (0.001, 0.016) (-0.115, -0.047) (0.037, 0.050) (-0.115, -0.019) (0.016, 0.028) (0.052, 0.071) (-0.154, -0.117) (-0.176, 0.049) | | | | | | | | |

Gulf Plains Cape York Princess- Coastal Plains

**Supplementary Table 6 Genetic diversity indices by bioregion generated from 959 crocodiles filtered using the Radiator pipeline with no MAC or HWE quality control filtering.** Genetic diversity indices for each of the nine bioregion populations, comprising 959 saltwater crocodiles and 4,312 SNPs. Reported are the number of individuals within each bioregion (N), number of monomorphic loci (M0), number of loci with minor allele frequency (MAF) less than 1%, allelic richness (Ar), observed heterozygosity (HO), unbiased expected heterozygosity (uHE), and inbreeding coefficient FIS and 95% bootstrap (200 replicates) confidence intervals (CI). Bioregion abbreviations are Norman-Flinders drainage (NFD), Albert-Leichhardt drainage (ALD), Mitchell-Gilbert drainage (MGD), Cooktown – Ayr (CA), Ayr – Proserpine – Rockhampton (APrR).

DAPC clusters

Bioregions 1 2 3 4 5

| Gulf Plains - ALD | 0 | 7 0 | | 0 | 0 |
| --- | --- | --- | --- | --- | --- |
| Gulf Plains - NFD | 0 | 101 0 | | 0 | 3 |
| Gulf Plains - MGD 0 | | 7 | 0 | 0 | 6 |
| North West Cape York 11 | | 8 | 0 | 1 | 322 |
| North East Cape York 10 | | 0 | 0 | 0 | 1 |
| Princess Charlotte Bay 205 0 | | | 1 | 1 | 1 |
| Coastal Plains - CA 10 5 Coastal Plains - APrR 0 0 | | | 1  77 | 147 6  0 0 | |
| Fitzroy 0 | | 0 | 0 16 1 | | |

**Supplementary Table 7 Summary of number of individuals in each bioregion classified into the five DAPC clusters.** Classification to a group is performed by allocating individuals with a posterior group probability greater than 0.5 to that group. Nearly all posterior probabilities were ≈ 1 for group assignment for each individual.

DAPC clusters

Bioregions 1 2 3 4 5 6 7 8

| Gulf Plains - ALD 0 7 0 0 0 0 0 | 0 |
| --- | --- |
| Gulf Plains - NFD 0 101 0 3 0 0 0 | 0 |
| Gulf Plains - MGD 0 6 0 7 0 0 0 | 0 |
| North West Cape York 1 8 0 263 16 54 0 | 0 |
| North East Cape York 0 0 0 1 10 0 0 | 0 |
| Princess Charlotte Bay 0 0 89 1 117 0 0 | 1 |
| Coastal Plains - CA 136 5 3 6 18 0 0 | 1 |
| Coastal Plains - APrR 0 0 0 0 0 0 0 | 77 |
| Fitzroy 1 0 0 1 0 0 15 0 | |

**Supplementary Table 8 Summary of number of individuals in each bioregion classified into the 8 (minimum BIC) DAPC clusters.** Classification to a group is performed by allocating individuals with a posterior group probability greater than 0.5 to that group. Nearly all posterior probabilities were ≈ 1 for group assignment for each individual.

POP FSP 2nd-degree

| Sim. scenario | *φ* | *k*(0) | *k*(2) | *φ* | *k*(0) | *k*(2) | *φ* | *k*(0) | *k*(2) |
| --- | --- | --- | --- | --- | --- | --- | --- | --- | --- |
| 1% | 0.259 | 0.000 | 0.038 | – | – | – | 0.134 | 0.487 | 0.022 |
| 0.1% | 0.257 | 0.000 | 0.035 | – | – | – | 0.134 | 0.489 | 0.023 |
| 0.01% | 0.258 | 0.000 | 0.041 | – | – | – | 0.136 | 0.483 | 0.027 |
| 1% - litter | 0.254 | 0.002 | 0.015 | 0.253 | 0.242 | 0.223 | 0.135 | 0.482 | 0.013 |
| 0.1% - litter | 0.258 | 0.001 | 0.014 | 0.256 | 0.238 | 0.233 | 0.136 | 0.477 | 0.016 |
| 0.01% - litter | 0.264 | 0.001 | 0.022 | 0.260 | 0.250 | 0.242 | 0.139 | 0.477 | 0.019 |

**Supplementary Table 9 Comparison of expected kinship coefficient** (*φ*)**,** *k*(0) **and** *k*(2) **estimates by relationship type from PC-Relate across simulation scenarios.** The values presented in the table for each of the estimators is the mean of the estimated kinship statistics from the simulation scenarios over the 50 replicates.

POP FSP 2nd-degree

| Sim. scenario | *φ* | *k*(0) | *k*(2) | *φ* | *k*(0) | *k*(2) | *φ* | *k*(0) | *k*(2) |
| --- | --- | --- | --- | --- | --- | --- | --- | --- | --- |
| 1% | 0.021 | 0.000 | 0.068 | – | – | – | 0.041 | 0.164 | 0.053 |
| 0.1% | 0.021 | 0.000 | 0.064 | – | – | – | 0.041 | 0.165 | 0.055 |
| 0.01% | 0.022 | 0.000 | 0.074 | – | – | – | 0.042 | 0.166 | 0.058 |
| 1% - litter | 0.023 | 0.010 | 0.055 | 0.056 | 0.135 | 0.117 | 0.043 | 0.164 | 0.045 |
| 0.1% - litter | 0.023 | 0.003 | 0.059 | 0.054 | 0.134 | 0.117 | 0.044 | 0.164 | 0.050 |
| 0.01% - litter | 0.026 | 0.002 | 0.069 | 0.057 | 0.142 | 0.125 | 0.047 | 0.168 | 0.056 |

**Supplementary Table 10 Comparison of standard deviation kinship coefficient** (*φ*)**,** *k*(0) **and** *k*(2) **estimates by relationship type from PC-Relate across simulation scenarios.** The values presented in the table for each of the estimators is the average standard deviation of the estimated kinship statistics from the simulation scenarios over the 50 replicates.

|  | Gulf Plains | | | | Cape York | | Princess- | Coastal Plains | | | |
| --- | --- | --- | --- | --- | --- | --- | --- | --- | --- | --- | --- |
|  | ALD | | NFD | MGD | North West | North East | Charlotte Bay | CA | APrR | | Fitzroy |
| Gulf Plains - ALD | | $\frac{(0, 1, 8)}{21}$ | $\frac{(0, 0, 0)}{728}$ | $\frac{(0, 0, 0)}{91}$ | $\frac{(0, 0, 0)}{2394}$ | $\frac{(0, 0, 0)}{77}$ | $\frac{(0, 0, 0)}{1456}$ | $\frac{(0, 0, 0)}{1183}$ | $\frac{(0, 0, 0)}{539}$ | | $\frac{(0, 0, 0)}{119}$ |
| Gulf Plains - NFD | |  | $\frac{(0, 167, 245)}{5356}$ | $\frac{(0, 2, 3)}{1352}$ | $\frac{(0, 0,2)}{35568}$ | $\frac{(0, 0, 0)}{1144}$ | $\frac{(0, 0, 0)}{21632}$ | $\frac{(0, 0, 0)}{17576}$ | $\frac{(0, 0, 0)}{8008}$ | | $\frac{(0, 0, 0)}{1768}$ |
| Gulf Plains - MGD | |  |  | $\frac{(0, 4, 11)}{78}$ | $\frac{(0, 0, 0)}{4446}$ | $\frac{(0, 0, 0)}{143}$ | $\frac{(0, 0, 0)}{2704}$ | $\frac{(0, 0, 0)}{2197}$ | $\frac{(0, 0, 0)}{1001}$ | | $\frac{(0, 0, 0)}{221}$ |
| North West Cape York | |  |  |  | $\frac{(10, 234, 862)}{58311}$ | $\frac{(0, 2, 5)}{3762}$ | $\frac{(0, 0, 2)}{71136}$ | $\frac{(2, 1, 21)}{57798}$ | $\frac{(0, 0, 0)}{26334}$ | | $\frac{(0, 0, 0)}{5814}$ |
| North East Cape York | |  |  |  |  | $\frac{(0, 0, 2)}{55}$ | $\frac{(0, 0, 2)}{2288}$ | $\frac{(0, 0, 0)}{1859}$ | $\frac{(0, 0, 0)}{847}$ | | $\frac{(0, 0, 0)}{187}$ |
| Princess Charlotte Bay | |  |  |  |  |  | $\frac{(2, 129, 505)}{21528}$ | $\frac{(0, 7, 34)}{35152}$ | $\frac{(1, 41, 29)}{16016}$ | | $\frac{(0, 0, 0)}{3536}$ |
| Coastal Plains - CA | |  |  |  |  |  |  | $\frac{(7, 168, 523)}{14196}$ | $\frac{(1 , 6, 65)}{13013}$ | | $\frac{(0, 0, 0)}{2873}$ |
| Coastal Plains - APrR | |  |  |  |  |  |  |  | $\frac{(3, 1020, 1392)}{2926}$ | | $\frac{(0, 0, 0)}{1309}$ |
| Fitzroy | |  |  |  |  |  |  |  |  |  | $\frac{(0, 79, 179)}{136}$ |

**Supplementary Table 11 Summary of reported PC-Relate kin-pairs classified using the criteria of Manichaikul et al., 2010 versus number of pairwise comparisons between and within bioregions.** Each numerator in the cell has a vector of three numbers, which corresponds to the number of POPs, FSPs and 2nd-degree relatives detected within or between bioregions respectively. The denominator corresponds to the number of comparisons performed to detect these number of kin. Grey cells indicate those where kin were detected. Bioregion abbreviations are Albert-Leichhardt drainage (ALD), Norman-Flinders drainage (NFD), Mitchell-Gilbert drainage (MGD), Cooktown – Ayr (CA), Ayr – Proserpine – Rockhampton (APrR). Individuals (N = 2) from the Coastal Plains - CMC bioregion are integrated into the Princess Charlotte Bay bioregion as the results were uninformative.

37

| **I1 I2 *φ k*(0) *k*(2)** | | | **Euclidean SVM BIO1 BIO2 I1 Sex I2 Sex I1 CL** | | | | | | **I2 CL** | **Distance 1 – 2 I1 Date I2 Date** | | | **I1 LC I2 LC ∆ Days** | | |
| --- | --- | --- | --- | --- | --- | --- | --- | --- | --- | --- | --- | --- | --- | --- | --- |
| IND1123 IND1148 0.31 0.020 0.068 | | | POP | POP CPCA CPCA M | | | - | Babinda Ck | Russell Rvr | 9.00 | 29/10/20 6/5/21 | | 15 | 7 | 189 |
| IND1123 IND1144 0.31 0.0190 0.150 | | | POP | POP CPCA CPCA M | | | - | Babinda Ck | Russell Rvr | 5.30 | 29/10/20 6/5/21 | | 15 | 7 | 189 |
| IND3 IND194 0.29 0.0280 0.100 | | | POP | POP PCB CPAPrR M | | | - | Old Faithful, Lakefield | Proserpine Rvr | 750 | 1/8/18 24/10/19 14 | | | 2 | 449 |
| IND178 IND74 0.28 0.000 0.170 | | | POP | POP CPAPrR CPAPrR - | | | M | Proserpine Rvr | Proserpine Rvr | 0.98 | 24/10/19 20/2/19 1 | | | 14 | 246 |
| IND1224 IND1227 0.28 0.0250 0.028 | | | POP | POP NWCY | NWCY | M | F | Mungkan Rocks | Mungkan | 1.00 | 6/11/11 11/10/13 - | | | - | 705 |
| IND244 IND472 0.27 0.0083 0.052 | | | POP | POP NWCY | NWCY | - | F | Tentpole Ck | Tent Pole Ck | 16.0 | 1/11/19 11/11/98 1 | | | - | 7660 |
| IND1123 IND60 0.27 0.0190 -0.005 POP | | | | POP CPCA | CPCA | M | M | Babinda Ck | Barron Rvr | 60.0 | 29/10/20 16/11/18 15 | | | 6 | 713 |
| IND1123 IND1147 0.27 0.0190 -0.0064 POP | | | | POP CPCA | CPCA | M | - | Babinda Ck | Russell Rvr | 8.30 | 29/10/20 6/5/21 15 | | | 2 | 189 |
| IND782 IND825 | | 0.27 0.0360 0.0260 | POP | POP PCB | PCB | F | F | Seven Mile Waterhole | Lakefield NP | 0.00 | 25/10/05 15/09/97 4 | | | 2 | 2962 |
| IND245 IND333 | | 0.27 0.0360 0.0190 | POP | POP NWCY | NWCY | - | - | Tentpole Ck | Tentpole Ck | 6.70 | 1/11/19 | 2/11/19 6 | | 1 | 1 |
| IND295 IND312 | | 0.26 0.0180 0.0250 | POP | POP NWCY | NWCY | - | - | Tentpole Ck | Tentpole Ck | 0.19 | 1/11/19 | 2/11/19 8 | | 1 | 1 |
| IND349 IND472 | | 0.26 0.0170 -0.0180 | POP | POP NWCY | NWCY | - | F | Tentpole Ck | Tentpole Ck | 8.00 | 2/11/19 | 11/11/98 1 | | - | 7661 |
| IND6 IND436 | | 0.25 0.0000 -0.0035 | POP | POP CPCA | CPAPrR F | | F | Boar Ck, Tully | Proserpine Rvr | 400 | 1/8/18 | 6/08/98 7 | | 2 | 7300 |
| IND1153 IND693 | | 0.25 0.0097 0.0340 | POP | POP CPCA | CPCA - | | M | Russell Rvr | Edmund Kennedy NP 97.0 | | 6/5/21 | 19/11/98 3 | | 2 | 8204 |
| IND245 | IND331 | 0.25 0.0270 0.0061 | POP | POP NWCY | NWCY - | | - | Tentpole Ck | Tentpole Ck | 6.90 | 1/11/19 | 2/11/19 6 | | 1 | 1 |
| IND295 | IND336 | 0.24 0.0180 -0.027 | POP | POP NWCY | NWCY - | | - | Tentpole Ck | Tentpole Ck | 2.30 | 1/11/19 | 2/11/19 8 | | 1 | 1 |
| IND263 | IND314 | 0.24 0.0260 -0.023 | POP | POP NWCY | NWCY - | | - | Tentpole Ck | Tentpole Ck | 2.50 | 1/11/19 | 2/11/19 1 | | 7 | 1 |
| IND291 | IND295 | 0.24 0.0270 -0.013 | POP | POP NWCY | NWCY - | | - | Tentpole Ck | Tentpole Ck | 0.47 | 1/11/19 | 1/11/19 1 | | 8 | 0 |
| IND175 | IND451 | 0.24 0.0088 0.0016 | POP | POP CPAPrR CPAPrR - | | | F | Proserpine Rvr | Proserpine Rvr | 39.0 | 24/10/19 26/08/98 1 | | | 1 | 7729 |
| IND607 | IND621 | 0.23 0.0350 0.0300 | POP | POP NWCY CPCA M | | | M | Captive bred, Edward Rvr Cairns | | 500 | 15/2/13 | 4/06/99 - | | 1 | 5005 |
| IND607 | IND763 | 0.23 0.0260 0.0270 | POP | POP NWCY CPCA M | | | M | Captive bred, Edward Rvr Cairns | | 500 | 15/2/13 | 13/10/99 - | | 4 | 4874 |
| IND231 | IND314 | 0.23 0.0350 0.0360 | POP | POP NWCY NWCY - | | | - | Tentpole Ck Tentpole Ck | | 2.40 | 1/11/19 | 2/11/19 1 | | 7 | 1 |

38

**Supplementary Table 12 Summary of parent-offspring pairs detected in PC-Relate kinship analysis of Queensland saltwater crocodile population.** Each row represents metadata from each POP. From left to right the metadata include the individual identifiers, PC-Relate kinship and sharing probabilities for the pair, the kinship classification by the Euclidean and SVM classifiers, the bioregions of each individual, the sex of each individual, the capture location within bioregion, the distance between the latitude longitude coordinates for each pair in kilometres, the sampling date of each individual, the length class of each individual and the number of days between sampling dates for each pair. The bioregions abbreviations are Coastal Plains - Cooktown – Ayr (CPCA), North-West Cape York (NWCY), Princess Charlotte Bay (PCB), and Coastal Plains - Ayr – Proserpine – Rockhampton (CPAPrR).

| **Bioregion** | **Total Samples**  **(Pre 2000/Post 2000)** | | **1990 - 2000s (P/T)** | | **2014 - 2021 (P/T)** | **% 1990 - 2000s (P/T)** | | | | **% 2014 - 2021**  **(P/T)** |
| --- | --- | --- | --- | --- | --- | --- | --- | --- | --- | --- |
| North West Cape York | | 200/206 | 0/1 | 0/0 | | | 0/0.49 | | 0/0 | |
| Princess Charlotte Bay | | 82/157 | 0/0 | 1/1 | | | 0/0 | | | 0.64/0.64 |
| Coastal Plains - CA | | 107/149 | 12(5)/3 | 10/8 | | | 11.2(4.6)/2.8 | 6.7/5.3 | | |
| Fitzroy | | 7/16 | 0/0 | 0/2 | | | 0/0 | | 0/12.5 | |

**Supplementary Table 13 Summary of counts and rates of possible and likely translocated individuals partitioned by period before and after translocation stopped (after 1999) being used in the Queensland crocodile management program.** The total samples column is the number of samples from the total set of 1,176 samples taken for the genetic analyses in the pre and post 2000 periods from the bioregions in which possible (P) and likely (T) translocated individuals were recorded. The following two columns detail the number of possible and likely translocations for each period partition by possible or likely. The final two columns show the percentage of total samples for that period that were translocated. The red numbers correspond to the number of possible translocations if only one individual is retained from a single nest translocated event of which all individuals were siblings (validated with the kinship analyses).

| ALD | | NFD | MGD | North-West | North-East | Charlotte Bay CA | | APrR | Fitzroy |
| --- | --- | --- | --- | --- | --- | --- | --- | --- | --- |
| *N* 7 | | 104 | 13 | 341 | 11 | 206 | 136 | 77 | 15 |
| *M*_0_ 550 | | 75 | 312 | 16 | 332 | 43 | 77 | 483 | 1304 |
| MAF *<* 1% 550 | | 128 | 312 | 104 | 332 | 136 | 198 | 651 | 1304 |
| *A_r_* | 1.570 | 1.660 | 1.630 | 1.660 | 1.620 | 1.660 | 1.630 | 1.520 | 1.320 |
| *H_O_* | 0.242 | 0.249 | 0.247 | 0.240 | 0.243 | 0.248 | 0.231 | 0.240 | 0.141 |
| *uH_E_* | 0.233 | 0.247 | 0.242 | 0.244 | 0.247 | 0.249 | 0.237 | 0.200 | 0.121 |
| *F_IS_*  *F_IS_* - CI | -0.110 -0.006 -0.058 0.020 -0.033 0.004 0.027 -0.147 -0.141  (-0.293, -0.109) (-0.019, -0.003) (-0.126, -0.056) (0.012, 0.024) (-0.131, -0.034) (-0.006, 0.008) (0.012, 0.032) (-0.181, -0.134) (-0.263, -0.140) | | | | | | | | |

Gulf Plains Cape York Princess- Coastal Plains

**Supplementary Table 14 Genetic diversity indices by bioregion generated from 910 crocodiles with probable translocations removed.** Genetic diversity indices for each of the nine bioregion populations, comprising 910 saltwater crocodiles and 2,958 SNPs. Reported are the number of individuals within each bioregion (N), number of monomorphic loci (M0), number of loci with minor allele frequency (MAF) less than 1%, allelic richness (Ar), observed heterozygosity (HO), unbiased expected heterozygosity (uHE), and inbreeding coefficient FIS and 95% bootstrap (200 replicates) confidence intervals (CI). Bioregion abbreviations are Albert-Leichhardt drainage (ALD), Norman-Flinders drainage (NFD), Mitchell-Gilbert drainage (MGD), Cooktown – Ayr (CA), Ayr – Proserpine – Rockhampton (APrR).

|  | Gulf Plains | | | | Cape York | | Princess- | Coastal Plains | | | |
| --- | --- | --- | --- | --- | --- | --- | --- | --- | --- | --- | --- |
|  | ALD | | NFD | MGD | North West | North East | Charlotte Bay | CA | APrR | | Fitzroy |
| Gulf Plains - ALD | | $\frac{(0, 1, 3)}{21}$ | $\frac{(0, 0, 0)}{728}$ | $\frac{(0, 0, 0)}{91}$ | $\frac{(0, 0, 0)}{2387}$ | $\frac{(0, 0, 0)}{77}$ | $\frac{(0, 0, 0)}{1442}$ | $\frac{(0, 0, 0)}{952}$ | $\frac{(0, 0, 0)}{539}$ | | $\frac{(0, 0, 0)}{105}$ |
| Gulf Plains - NFD | |  | $\frac{(0, 137, 144)}{5356}$ | $\frac{(0, 2, 2)}{1352}$ | $\frac{(0, 0,1)}{35464}$ | $\frac{(0, 0, 0)}{1144}$ | $\frac{(0, 0, 0)}{21424}$ | $\frac{(0, 0, 0)}{14144}$ | $\frac{(0, 0, 0)}{8008}$ | | $\frac{(0, 0, 0)}{1560}$ |
| Gulf Plains - MGD | |  |  | $\frac{(0, 4, 6)}{78}$ | $\frac{(0, 0, 0)}{4433}$ | $\frac{(0, 0, 0)}{143}$ | $\frac{(0, 0, 0)}{2678}$ | $\frac{(0, 0, 0)}{1768}$ | $\frac{(0, 0, 0)}{1001}$ | | $\frac{(0, 0, 0)}{195}$ |
| North West Cape York | |  |  |  | $\frac{(10, 176, 4682)}{57970}$ | $\frac{(0, 2, 2)}{3751}$ | $\frac{(0, 0, 0)}{70246}$ | $\frac{(0, 0, 0)}{46376}$ | $\frac{(0, 0, 0)}{26257}$ | | $\frac{(0, 0, 0)}{5115}$ |
| North East Cape York | |  |  |  |  | $\frac{(0, 0, 0)}{55}$ | $\frac{(0, 0, 1)}{2266}$ | $\frac{(0, 0, 0)}{1496}$ | $\frac{(0, 0, 0)}{847}$ | | $\frac{(0, 0, 0)}{165}$ |
| Princess Charlotte Bay | |  |  |  |  |  | $\frac{(1, 84, 282)}{21115}$ | $\frac{(0, 0, 0)}{28016}$ | $\frac{(0, 0, 0)}{15862}$ | | $\frac{(0, 0, 0)}{3090}$ |
| Coastal Plains - CA | |  |  |  |  |  |  | $\frac{(5, 81, 301)}{9180}$ | $\frac{(0 , 0, 0)}{10472}$ | | $\frac{(0, 0, 0)}{2040}$ |
| Coastal Plains - APrR | |  |  |  |  |  |  |  | $\frac{(2, 638, 1357)}{2926}$ | | $\frac{(0, 0, 0)}{1155}$ |
| Fitzroy | |  |  |  |  |  |  |  |  |  | $\frac{(0, 25, 68)}{105}$ |

**Supplementary Table 15 Summary of reported PC-Relate kin-pairs versus number of pairwise comparisons between and within bioregions with possible translocated individuals removed.** Each numerator in the cell has a vector of three numbers, which correspond to the number of POPs, FSPs and 2nd-degree relatives detected within or between bioregions respectively. The denominator corresponds to the number of comparisons performed to detect this number of kin. Grey cells indicate those where kin were detected. Bioregion abbreviations are Albert-Leichhardt drainage (ALD), Norman-Flinders drainage (NFD), Mitchell-Gilbert drainage (MGD), Cooktown – Ayr (CA), Ayr – Proserpine – Rockhampton (APrR). Individuals (N=2) from the Coastal Plains - CMC bioregion are integrated into the Princess Charlotte Bay bioregion as the results were uninformative.
